# Phase IB Study of Oral Selinexor in Combination with Rituximab and Platinum Chemotherapy in Patients with Relapsed/Refractory B-Cell Lymphoma—Final Analysis

**DOI:** 10.3390/cancers16152672

**Published:** 2024-07-26

**Authors:** Marie Maerevoet, Olivier Casasnovas, Guillaume Cartron, Franck Morschhauser, Catherine Thieblemont, Kamal Bouabdallah, Pierre Feugier, Vanessa Szablewski, Stephanie Becker, Herve Tilly

**Affiliations:** 1 Hopital Universitaire de Bruxelles, Institut Jules Bordet, Université Libre de Bruxelles, 1070 Brussels, Belgium; 2 Central Hospital University (CHU) Dijon Bourgogne, 21000 Dijon, France; olivier.casasnovas@chu-dijon.fr; 3 Central Hospital University (CHU) Montpellier, Hôpital Saint Eloi, 34295 Montpellier, France; g-cartron@chu-montpellier.fr; 4 Centre Hospitalier Régional University (CHRU) de Lille, Hôpital Claude Huriez, 59000 Lille, France; franck.morschhauser@chru-lille.fr (F.M.); vszmed@hotmail.fr (V.S.); 5 Hôpital Saint Louis AP-HP, 75010 Paris, France; catherine.thieblemont@aphp.fr; 6 Hematology and Cell Therapy Department, University Hospital of Bordeaux, 33000 Bordeaux, France; krimo.bouabdallah@chu-bordeaux.fr; 7 Centre Hospitalier Régional University (CHRU) Nancy, 54511 Vandœuvre-lès-Nancy, France; p.feugier@chu-nancy.fr; 8 Nuclear Medicine Department and QuantiF-LITIS Laboratory (EA 4108-FR CNRS 3638), Centre Henri Becquerel, 76038 Rouen, France; stephanie.becker@chb.unicancer.fr; 9 Hematology Department and U1245, Centre Henri Becquerel, 76038 Rouen, France; herve.tilly@chb.unicancer.fr

**Keywords:** selinexor, diffuse large B-cell lymphoma, relapsed, RGDP

## Abstract

**Simple Summary:**

The chemotherapy combination rituximab, gemcitabine, and dexamethasone (R-GDP), followed by high-dose chemotherapy and autologous stem cell transplantation, is one of the standards of care for relapsed or refractory B-cell non-Hodgkin lymphoma (R/R NHL). Complete metabolic response before transplantation is the most important prognosis factor for a long duration of remission. Selinexor is an oral, selective inhibitor of the nuclear export compound (XPO1). For heavily pretreated patients with DLBCL, the single drug selinexor has previously shown an overall response rate of 29%. In this study, we evaluated selinexor in combination with RGDP for patients with R/R B-cell lymphoma. The results from our phase I clinical study indicate that weekly selinexor plus RGDP has a generally tolerable safety profile and durable efficacy in R/R B-NHL.

**Abstract:**

Purpose: Selinexor is an oral selective inhibitor of exportine-1 (XPO1) with efficacy as a single agent in heavily pretreated diffuse large B-cell lymphoma (DLBCL). We conducted a study investigating the combination of selinexor with rituximab and platinum-based chemotherapy in B-cell lymphoma. Patients and methods: We conducted a phase 1b, dose-escalation, and expansion trial, which enrolled patients with relapsed or refractory B-cell non-Hodgkin lymphoma. Patients received oral selinexor according to a 3 + 3 design in combination with rituximab and dexamethasone, high-dose cytarabine, oxaliplatine (DHAOX) or gemcitabine, dexamethasone, and cisplatin (GDP) chemotherapy. Results: A total of 39 patients were enrolled, 27 during the escalation phase and 12 during the expansion phase. Most patients had diffuse large B-cell lymphoma (DLBCL; 77%). Group R-DHAOX was prematurely closed to inclusion due to a recommendation from the French drug agency, independent of this trial. A recommended phase 2 dose (RP2D) of selinexor in association with R-GPD was established at 40 mg on days 1, 8, and 15 of each 21-day cycle. In a population of 18 patients treated at this dose of selinexor, the most frequent grade 3–4 adverse events were hematological. With this regimen, seven obtained a complete metabolic response and five a partial response. The median PFS was 5.8 months. Conclusions: Among the patients with R/R B-cell lymphoma, selinexor at a weekly dose of 40 mg with R-GDP is feasible for outpatients, with a generally acceptable safety profile.

## 1. Introduction

Selinexor is a selective oral XPO1 inhibitor that induces the nuclear accumulation and activation of tumor suppressor proteins and reductions in Bcl2, Bclx, and C-Myc oncoprotein concentrations. For heavily pretreated patients with DLBCL, in the SADAL study (NCT02227251), the single drug selinexor showed an investigator-assessed overall response rate (ORR) of 29% [1]. In June 2020, the FDA granted accelerated approval to selinexor for adults with R/R DLBCL, not otherwise specified, including DLBCL arising from follicular lymphoma, after at least two lines of systemic therapy [2].

High-dose chemotherapy followed by autologous stem cell transplantation (ASCT) is a standard treatment for R/R DLBCL [3]. In comparison with R-DHAP (rituximab, dexamethosone, high-dose cytarabine, and cisplatin), treatment with R-GDP (rituximab, dexamethasone, gemcitabine, and cisplatin) is associated with a noninferior response rate; similar transplantation rate, event-free survival, and overall survival; less toxicity and hospitalization; and superior quality of life [4]. R-DHAOX, a combination where oxaliplatine is the platinum component, is an efficient regimen for R/R B-cell lymphoma [5].

We hypothesized that selinexor could be combined with platinum immunochemotherapy regimens and could increase the quality of response.

We report here the results of a phase 1b study aiming at determining the recommended dose, the safety, and initial efficacy of selinexor in combination with platinum immunochemotherapy regimens.

## 2. Patients and Methods

### Study Design and Patients

The SELINDA trial was a multicentric, open label, phase 1b design study, investigating dose escalation of oral selinexor, in combination with rituximab and dexamethasone, high-dose cytarabine, oxaliplatine (R-DHAOX) or gemcitabine, dexamethasone, and cisplatin (R-GDP) immunochemotherapy in relapsed or refractory B-cell non-Hodgkin lymphoma (NHL) (NCT02741388). This study was conducted in two steps: a dose-escalation phase designed to determine the recommended phase 2 dose (RP2D) of selinexor, followed by an expansion phase at this dose. This study was conducted at 8 sites across France and Belgium.

The study protocol was approved by ethics committees at participating institutions in accordance with the International Conference on Harmonization guidelines, including Good.

Good clinical practices and the ethical principles originating from the Declaration of Helsinki were followed. Informed consent was obtained from all patients.

The primary objective was to determine the RP2D of selinexor when given in combination with R-DHAOX or R-GDP. Secondary objectives were to assess efficacy (response, duration of response (DOR), progression-free survival (PFS), and overall survival (OS)).

During the dose-escalation phase, a safety review committee (SRC) met after completion of each cohort of 3 patients enrolled at a given dose level. An independent data monitoring committee (IDMC) including two hematologists and one statistician reviewed accumulating safety data during the conduct of the dose-escalation phase and confirmed the RP2D. Eligible patients were aged between 18 years and 70 years, able to receive high-dose chemotherapy according to the investigator’s opinion; with any histologically confirmed type of B-cell lymphoma; having received prior therapy with at least one but no more than two lines of treatment; having at least one single node or tumor lesion > 1.5 cm; having Eastern Cooperative Oncology Group (ECOG) performance status ≤ 2; possessing adequate renal function defined as creatinine clearance ≥ 70 mL/min and hematological function defined as neutrophil count ≥ 1.0 × 10^9^/L, and platelet count ≥ 75 × 10^9^/L (unless due to bone marrow involvement).

Exclusion criteria are described in the protocol (Supplementary Data S1).

## 3. Treatments

The phase 1b part of this study included 2 parallel treatment arms, with 3 planned cycles of immunochemotherapy in combination with selinexor doses ranging initially from 40 mg to 80 mg on days 1, 3, 8, and 11 of each 21-day cycle. The choice of the immunochemotherapy regimen was left to the investigator’s decision before a patient’s inclusion. R-DHAOX consisted of IV rituximab 375 mg/m^2^ day 1; IV oxaliplatine 130 mg/m^2^, day 1; IV cytarabine 2 × 2 g/m^2^ day 2; IV or oral dexamethasone 40 mg days 1–4. R-GDP consisted of IV rituximab 375 mg/m^2^ day 1, IV cisplatin 75 mg/m^2^ day 1, IV gemcitabine 1000 mg/m^2^ days 1 and 8, IV or oral dexamethasone 40 mg days 1–4. In the protocol, G-CSF use and antibiotic prophylaxis for pneumocystis carinii and herpes zoster virus were recommended. Supportive care with 5HT3 was recommended from day 1 to 3 and additional antinausea/vomiting therapy such as neurokinin inhibitor, receptor agonist, or IPP could be administrated per institutional guidelines. Stem cell collection after cycle 2 or 3, followed by high-dose chemotherapy and stem cell transplantation, was optional. Selinexor dose escalation was conducted under a modified 3 + 3 design with at least 3 patients per arm enrolled in each cohort. The dose-limiting toxicity (DLT) assessment period began with the first dose of selinexor and ended immediately prior to the initiation of the second cycle. Determination of the maximum tolerated dose (MTD) is described in the protocol (Protocol, Supplementary Data S1).

## 4. Safety

Laboratory assessments and adverse event (AE) monitoring were used to assess safety. AEs were coded using the Common Terminology Criteria for Adverse Events (CTCAE) grading system v4.03. Only toxicities occurring during the DLT period were used for the purposes of defining MTD. However, toxicities that occurred in all cycles were considered in the overall decisions of the SRC and IDMC.

Due to the expected toxicity of standard immunochemotherapy regimens, only adverse events (AEs) of grade ≥ 3 (hematological and nonhematological toxicities) and all AEs of grade ≥ 2 for renal and neurologic toxicities, as well as all toxicities, regardless of the grade, resulting in a delay > 2 weeks of the initiation of a cycle or reduction in selinexor dose, were recorded. All AEs of grade ≥ 2 related or potentially related to selinexor such as diarrhea, anorexia/weight loss or nausea/vomiting and fatigue/asthenia also had to be reported. Hematological assessment was performed on days 1, 8, and 15 of each cycle.

## 5. Efficacy

Tumors were evaluated by the investigators according to a modified version of the Lugano 2014 criteria [6] performed at baseline, and in the 10 days after the end of cycle 3 or after the last drug administration, in case of permanent study discontinuation for any cause. Positron emission tomography was mandatory at screening and at the end of treatment (EOT). After the EOT, assessments were performed every 3 months during the first year, then every 6 months until the end of this study.

## 6. Statistical Analysis

No formal power calculations were performed to predetermine sample size. The escalation process of the sample size for the dose finding was based on a modified 3 + 3 design to guide RP2D. The total number of patients enrolled depended on the outcome of the actual dose. For the dos- escalation phase, a minimum of 6 and a maximum of 9 patients were enrolled to determine RP2D. In the expansion phase, 12 patients were to be enrolled at the RP2D. Analysis was performed by the Lymphoma Study Association Recherche Clinique (LYSARC).

## 7. Results

### 7.1. Patient Disposition

**Figure 1:**
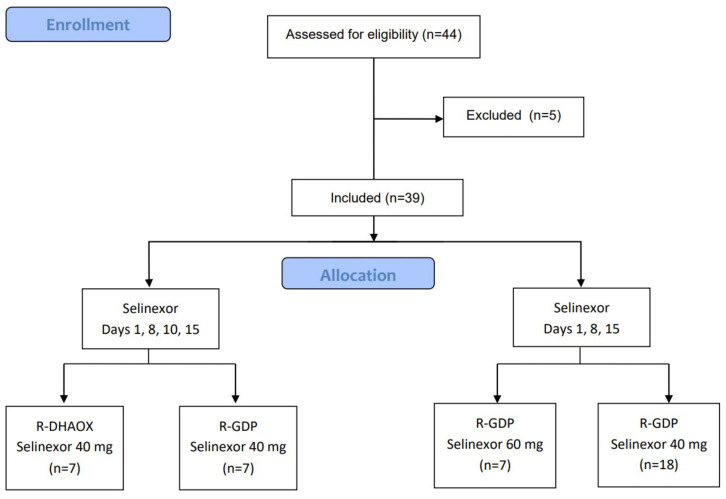
Consort diagram: SELINDA study.

The data cutoff was 8 February 2022. Forty-four (44) patients were enrolled in the study between 15 November 2016, and 14 January 2021. Five patients did not receive selinexor and were excluded for safety analysis. Overall, 39 patients received at least one dose of selinexor, 14 in the first dos- escalation phase 1b (7 treated with R-DHAOX and selinexor (days 1, 3, 8, and 10) at dose level 1 (40 mg) and 7 treated with R-GDP and selinexor (days 1, 3, 8, and 10) at dose level 1 (40 mg). In a second dose-escalation phase, at the weekly selinexor dose (days 1, 8, and 15), seven patients were treated with R-GDP at dose level 2 (60 mg), and six patients were treated at dose level 1 (40 mg). Twelve patients were treated in the expansion phase with R-GDP at the weekly RP2D (40 mg) dose of selinexor (Figure 1). As a result, 18 patients received treatment with R-GDP and the RPD2 dose of selinexor (Figure 1, consort diagram).

### 7.2. DLTs, Modification of Selinexor Dosing Schedule, and RP2D

In both immunochemotherapy groups, the initial starting dose of selinexor was 40 mg for days 1, 3, 8, and 10 for three cycles of 21 days. Group R-DHAOX was prematurely closed to inclusion due to a safety alert coming from the French “Agence Nationale de Sécurité du Médicament et des produits de santé” (ANSM) for potential risks of veino occlusive disease in patients treated with R-DHAOx followed by high-dose chemotherapy and ASCT in different studies for mantle cell lymphoma.

Although no DLT was described with the biweekly regimen of 40 mg selinexor and R-GDP, the incidence of cumulative cytopenias led the SCR to recommend a new weekly administration regimen of selinexor on days 1, 8, and 15. In the R-GDP group at weekly selinexor dose level 2 (60 mg), two patients experienced neutropenia grade 4 defined as DLT. No DLT was observed in the six patients treated weekly with 40 mg of selinexor. Therefore, the RP2D for weekly selinexor in combination with R-GDP was determined to be 40 mg on days 1, 8, and 15 of a 21-day cycle for three cycles. This dose was then used in the 12 patients in the expansion cohort.

We focused on the safety and efficacy of selinexor when administered at the RP2D in the 18 patients included in this cohort.

### 7.3. RP2D Cohort Patient Characteristics

The patients’ characteristics are shown in Table 1. The median age was 59.3 years (52–66). Most patients were men (88%), 15 had a diagnosis of diffuse large B-cell lymphoma (83%), and 3 had a diagnosis of indolent lymphoma. Most patients had an Ann Arbor Stage III–IV (78%), and a performance status < 2 (95%). Most (72%) patients received only one prior therapy; three DLBCL patients were refractory to the first-line treatment. Twelve patients relapsed within 12 months after the last chemotherapy treatment, six of them received one prior therapy, and six others, a second line. Fourteen patients (77.8%) received the three planned cycles of RGDP; four patients discontinued RGDP, two for toxicities, one for COVID infection, and one for progression during the cycle 1; thirteen patients (72%) received three cycles of selinexor; five patients discontinued treatment, three for toxicities (one nausea, one neutropenia, two thrombocytopenia), one for COVID infection, and one for progression during the cycle 1. Nine patients (50%) received a 100% dose intensity of selinexor.

### 7.4. Safety of RP2D Cohort

As shown in Table 2, the most common all-grade AEs were hematologic disorders and creatinine elevation. Serious AEs grade 3–4 were hematologic or infectious: two (11%) patients with thrombocytopenia, two (11, 1%) with neutropenia, and one (5.6%) with respiratory tract infection. Three (16.7%) patients required platelet transfusion and two (11.1%) red blood cell transfusion. Most patients (13 (72%)) received growth factors.

In the total, during the three cycles, four patients experienced a least one adverse event leading to selinexor treatment discontinuation: two for thrombocytopenia during cycle 1 and 2, respectively; one for neutropenia during cycle 2; and one for nausea during cycle 1. Only one patient had a least one adverse event leading to discontinue immunochemotherapy. Six patients (33%) experienced a grade 1 reversible elevation of creatinine. No neuropathy, VOD event, or adverse event leading to death was observed.

### 7.5. Efficacy in the RP2D Cohort

Of the 18 patients, 17 were evaluable for response. The PET-CT overall response rate (ORR) at the EOT by central review assessment was 70% (12/17 patients), complete metabolic response (CMR) 41% (7/17 patients), and partial metabolic response (PMR) 29% (5/17 patients). Patients with DLBCL had a CMR of 28.5% (4/14 patients) and a PMR of 35.7% (5/14 patients). All the three patients with indolent lymphoma achieved CMR.

**Figure 2:**
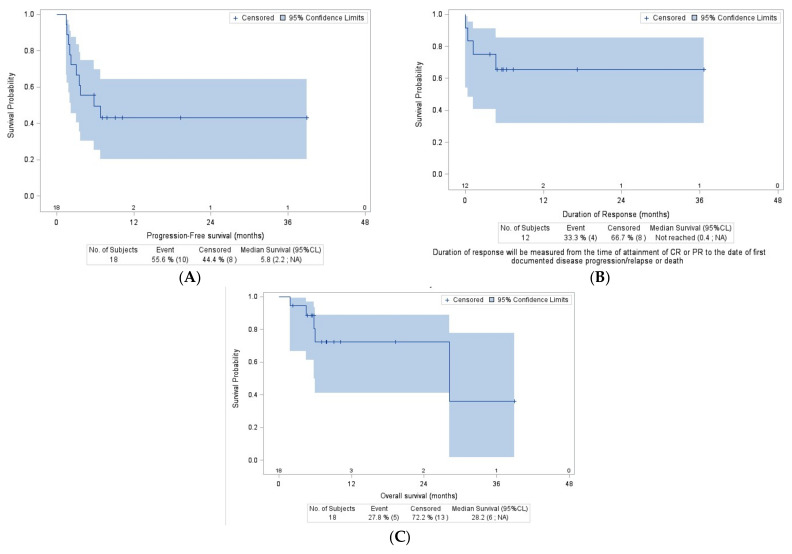
Outcomes of patients treated at the RP2D of selinexor and R-GDP. (**A**) Progression-free survival, RP2D cohort. (**B**) Duration of response, RP2D cohort. (**C**) Overall survival, RP2D cohort.

The median of the follow-up was 7.8 (1.9–38.9) months. The median time to progression from inclusion was 5.8 months, and the PFS at two years was 43% (95% CI, 20 to 64) (Figure 2A). The two-year response duration of the 12 responding patients in this cohort was 66% (95% CI, 32 to 86) (Figure 2B). The two-year overall survival of the R2PD cohort was 72% (95% CI, 41 to 88) (Figure 2C).

Per protocol, stem cell collection could be proposed after cycle 2 or 3, and high-dose chemotherapy and ASCT were optional. In this cohort, eight patients received a consolidation, four patients over the seven patients in complete remission underwent BEAM consolidation followed by ASCT, and four patients received a consolidation with platinum-based regimen.

## 8. Discussion

Salvage chemotherapy followed by high-dose chemotherapy and stem cell transplantation has been the standard of care for patients with R/R DLBCL [3]. For patients who had experienced relapse or were refractory to RCHOP, the ORCHARRD study compared ofatumumab versus rituximab salvage chemotherapy DHAP followed by autologous stem cell transplantation, showing that 2-year-PFS was 26% for rituximab plus chemotherapy [7]. In the NCIC-CTG LY.12 study, for patients with R/R aggressive lymphoma, in comparison with DHAP, treatment with GDP and ASCT was associated with a noninferior response rate, similar overall survival and transplantation rate, and less toxicity [4]. In this study, the most frequently serious adverse events, occurring in a least 5% of patients who received GDP at grade 3 or 4 NCICTC, were thrombo-embolic events (6%), fatigue (10%), nausea (4%), and febrile neutropenia (9%); 31% of patients required platelet transfusions.

The single agent selinexor was evaluated in the SADAL study in those heavily pretreated R/R DLBCL and demonstrated an overall response rate of 29% with a median duration of response of 9.3 months. Neutropenia, thrombocytopenia, and nausea grade 3 or 4 occurred, respectively, in 31%, 49%, and 6% of patients [1].

Several combinations have been studied to improve platinum-based immunochemotherapy regimens. There was no difference in efficacy found with DHAP plus ofatumumab as compared to rituximab and DHAP [7]. Ibrutinib plus R-DHAOX was associated with an increase in unacceptable toxicities [8]. A randomized phase 2 study of the combination of bortezomib and R- DHAP showed a complete remission rate of 33% in patients with R/R DLBCL but failed to improve the overall survival as compared to R-DHAP [9]. Recently, a combination of lenalidomide and R-GDP in a similar population showed an ORR of 60.2%, with 37.1% complete response and a median PFS of 9 months [10]. The results of a phase 2 combination study of a shorter course of venetoclax plus R-ICE including 66 patients with R/R DLBCL were recently reported—the most frequent grade ≥ 3 AEs were thrombocytopenia (70%), neutropenia (59%), and anemia (47%). In this study, the ORR was 81%, including a metabolic CR rate of 63%, median progression-free survival (PFS) was 25 months with a 2-year PFS estimate of 25%, and the median OS was 33 months [11].

We evaluated the safety and feasibility of the XPO1 inhibitor, selinexor, in combination with rituximab and RGDP chemotherapy in relapsed/refractory B-cell lymphoma. Overall, our findings indicated that the RP2D of selinexor in combination with R-GDP is a dose of 40 mg at days 1, 8, and 15 of a 21-day cycle, resulting in an overall metabolic response rate of 70% for B-cell lymphoma and 60% for the R/R DLBCL subgroup. Only four patients were offered stem cell harvesting followed by intensive treatment and ASCT. These were younger patients with DLBCL who responded to treatment. The grade 3–4 adverse events observed in the study were all reversible hematological—grade 3–4 neutropenia and thrombocytopenia occurred in 11% of patients; 16% of them received platelet transfusions. The toxicity observed in this study, hematological or infectious toxicity, did not appear to be more marked than that described in studies where R-GDP immune-chemotherapy was used alone [4]. Recently, Wang et al. presented a preliminary report of the efficacy of the combination of 40 mg weekly selinexor with RGDP or RICE for patients with 11R/R DLBCL with TP53 alterations [12,13]. Two patients of the first ten patients stopped treatment for gastrointestinal toxicities. In this study, the most common grade 3 or 4 adverse events were hematological toxicities, including neutropenia (63.6%) and thrombocytopenia (45.5%) [12]. Twenty patients receiving at least two cycles of treatment were evaluable; the CR rate was 25%—for the six patients in partial response and one patient in stable disease receiving CAR-T cell therapy, five achieved PR and two with CR [13].

The recent results from studies show that, in second-line treatment of DLBCL, CAR T-cell therapy can improve PFS compared to platinum-based immunochemotherapy followed by autologous transplantation [14], which will likely change the treatment landscape for relapsed/refractory patients. Some combinations of immunochemotherapy and targeted therapy, with improved efficacy and safety, will remain necessary to treat patients who cannot receive CAR T-cells or those who need a bridge therapy before CAR-T.

## 9. Conclusions

A weekly dose of 40 mg of selinexor in combination with R-GDP has a generally acceptable safety profile and response rate in patients with R/R B-cell lymphoma. The ongoing randomized phase 2/3 study evaluating R-GDP and selinexor (NCT04442022) will provide further information on the benefit–risk profile of this combination.

## Figures and Tables

**Table 1:** Baseline characteristic of 18 patients treated at RP2D. The 18 patients received selinexor at the weekly dose of 40 mg in combination with R-GDP.

Number of Patients	n = 18
Sex	
Female	2 (11.1%)
Male	16 (88.0%)
Age (range)	59.3 (52;66)
Performance status (ECOG)	
0–1	17 (95.4%)
2	1 (5.6%)
Ann Arbor stage	
I–II	4 (22.2%)
III–IV	14 (77.8%)
Bone marrow	
Not involved	1 (88.9%)
Involved	1 (5.6%)
Histology	
Diffuse large B-cell lymphoma	15 (83.3%)
Follicular lymphoma	2 (11.0%)
Marginal zone lymphoma	1 (5.5%)
Disease status	
Refractory	6 (33.3%)
Relapse	12 (66.6%)
Prior therapy	
1	13 (72.2%)
2	5 (27.8%)

**Table 2:** Adverse events in the 18 patients treated at RP2D.

Adverse Events n (%)	Pts. with a Least One AE 16 (89%)	Pts. with a Least One AE ≥ 3 2 (11.5%)
Neutropenia	10 (55.6%)	2 (11.5%)
Thrombocytopenia	8 (44.4%)	2 (11.5%)
Anemia	3 (16.7%)	0
Creatinine elevation	6 (33%)	0
Nausea	4 (22%)	0
Asthenia	4 (22.2%)	0
Respiratory tract infection	1 (5.6%)	1 (5.6%)
COVID 19	1 (5.6%)	0
Hepatic enzyme increase	1 (5.6%)	0
Pulmonary embolism	1 (5.6%)	0

## Data Availability

The data presented in this study are available on request from the first author (M.M.).

## Supplementary Materials

The following supporting information can be downloaded at: https://www.mdpi.com/article/10.3390/cancers16152672/s1, Data S1: Protocol.
