# Supplementary material for: Phase IB Study of Oral Selinexor in Combination with Rituximab and Platinum Chemotherapy in Patients with Relapsed/Refractory B-Cell Lymphoma—Final Analysis
Source: Cancers (Basel). 2024 Jul 26;16(15):2672. doi: 10.3390/cancers16152672 (PMC11311764; doi:10.3390/cancers16152672)
Supplement: Supplementary file 1 [file cancers-16-02672-s001.zip › cancers-3116742-supplementary.pdf]

CLINICAL STUDY PROTOCOL

SELINDA

(SELINexor-Dexamethasone-AraC/gemcitabine in B-cell lymphoma)

A Phase IB study of oral selinexor in adult patients with relapsed/refractory B-cell lymphoma receiving R-DHAOx or R-GDP

A STUDY SPONSORED BY:

LYSARC

LYSARC: THE LYMPHOMA ACADEMIC RESEARCH ORGANISATION

✉ : Centre Hospitalier Lyon Sud - Secteur Sainte Eugénie – Pavillon 6D

69495 Pierre Bénite Cedex– France

**COORDINATING INVESTIGATOR**<br>**CO- COORDINATING INVESTIGATOR**	Pr Hervé TILLY<br>Dr Marie MAEREVOET
**PROTOCOL WRITING COMMITTEE**	Pr Hervé TILLY, Dr Marie MAEREVOET, Florence BROUSSAIS, Christelle SEIGNE
**BIOMETRICS COORDINATOR**	Elodie GAT
**PATHOLOGICAL COORDINATOR**	Dr Vanessa SZABLEWSKI<br>Pr Christiane COPIE
**IMAGING COORDINATOR**	Dr Stéphanie BECKER
**COORDINATION CENTER**	LYSARC

**INCLUSION (SEE SECTION 10.2)**	http://study.lysarc.info
**SAE REPORTING (SEE SECTION 13.3)**<br>**AESI REPORTING (SEE SECTION 13.4)**	Fax: +33 (0) 3 59 11 01 86<br>Email: pharmacovigilance@lysarc.org

Version and date of Protocol: Final version V6.0 - 20 December 2019

EudraCT number: 2015-005612-15

CONFIDENTIALITY STATEMENT

The information contained in this document is the property of The Lymphoma Academic Research Organisation (LYSARC) and therefore is provided to you in confidence for review by you, your staff, an applicable Ethics Committee/Institutional Review and regulatory authorities. It is understood that the information will not be disclosed to others without prior written approval from the Lymphoma Academic Research Organisation (LYSARC), except to the extent necessary to obtain informed consent from those persons to whom the medication may be administered.

PROTOCOL APPROVAL & SIGNATURE PAGE

SELINDA

A PHASE IB STUDY OF ORAL SELINEXOR IN ADULT PATIENTS WITH RELAPSED/REFRACTORY B-CELL LYMPHOMA RECEIVING R-DHAOX OR R-GDP

LYSARC

Name: **Dr Pascal BILBAULT**<br>Title: General Manager	Date

COORDINATING INVESTIGATORS

Name: **Pr Hervé TILLY**<br>Title: Coordinating Investigator	Date
Name: **Dr Marie MAEREVOET**<br>Title: Co-Coordinating Investigator	Date

SYNOPSIS

**Study ID**<br><br>**EudraCT N°**	SELINDA (SELINexor-Dexamethasone-AraC/gemcitabine in B-cell lymphoma)<br><br>2015-005612-15
**Study title**	A Phase IB study of oral selinexor in adult patients with relapsed/refractory B-cell lymphoma receiving R-DHAOx or R-GDP
**Development phase**	Dose escalation phase IB with an exploratory expansion phase
**Investigational product**	Selinexor (KPT-330), selective inhibitor of nuclear export (SINE) compound
**Protocol Amendment version #6**	Final version – V6, December 20, 2019
**Protocol initial version**	Final version – V1.1 – June 13, 2016
**Sponsor**	LYSARC (The Lymphoma Academic Research Organisation)
**Coordinating investigator**<br><br>**Co-coordinating investigator**	Pr Hervé TILLY<br><br>Dr Marie MAEREVOET
**Sites**	8 centers during the escalation cohort phase; and during the expansion phase (including the centers of the dose escalation phase).
**Study Objectives and endpoints**	**<u>The primary objective</u>** of this study is to determine the Recommended Phase II Dose (RP2D) for selinexor when administered in adult patients with relapsed or refractory B-cell malignancies receiving R-DHAOX or R-GDP (rituximab + dexamethasone + gemcitabine + cisplatin), by assessing the maximum tolerated dose (MTD) observed during the dose escalation part of the study. Assessment of the MTD will be performed by the analysis of the dose-limiting toxicities (DLTs).<br><br>**<u>Secondary objectives and endpoints:</u>**<br>• Determine the occurrence of all grade ≥ 3 toxicities observed with the administration of selinexor in patients receiving R-DHAOx or R-GDP.<br>• Determine the occurrence of grade ≥ 2 renal toxicities and grade ≥ 2 neuropathy observed with the administration of selinexor in patients receiving R-DHAOx or R-GDP.<br>• To assess the preliminary efficacy of selinexor in patients receiving R-DHAOx or R-GDP as reflected by the overall response rate (ORR) after 3 cycles using Lugano classification criteria. Complete and partial response rates, progression free survival (PFS), response duration (DoR), time to next anti-lymphoma treatment (TTNLT) and overall survival (OS) will be also examined.

	**<u>Exploratory objectives and endpoints:</u>**<br>• For patients who proceed to Autologous Stem Cell Transplantation (ASCT), determine the feasibility of hematopoietic progenitor cell mobilization after selinexor administration with R-DHAOx or R-GDP (amount of peripheral CD34+ blood cells collected, rapidity and quality of engraftment). Collection and engraftment are optional and are not part of study treatment but data will be collected for exploratory purpose.<br>• In patients with DLBCL: response rate and PFS will be compared according to the GCB/non-GCB profile as assessed by immunohistochemistry according to Hans algorithm after central review at the LYSA-P.
**Study design**	This is an open-label, multicenter, dose escalation, phase IB study of selinexor administered in adult patients with relapsed/refractory B-cell malignancies receiving either R-DHAOx (Group A or R-GDP (Group B + Group B bis).<br><br>This dose escalation phase will be followed by an exploratory expansion phase in the same population with 12 patients enrolled in each group, who will receive selinexor at the RP2D.<br>The choice of the conventional immunotherapy regimen which was administered to each patient, R-DHAOx (Group A) or R-GDP (Group B + Group B bis), was left at the investigator's decision before patient's inclusion.<br><br>**<u>Part 1: Dose escalation phase</u>**<br>The "3+3" design was applied for dose escalation in groups A and B, and will be applied in group B Bis. The escalation will be performed in **:** Group B Bis as follows : R-GDP + oral Selinexor D1, D8 and D15 for 3 cycles (3- weeks cycles)<br><br>Group A and B were closed to inclusion after enrollment of 7 patients in each group due to new administration regimen of Selinexor.<br>Group A bis is closed to inclusion before any inclusion due to potential risks of veino occlusive disease with DHAOx treatment associated to ASCT procedure.<br>Different dose levels for selinexor administration will be examined sequentially in group B bis by the Safety review Committee (SRC): 3 doses of selinexor per 3-week cycle at **40 mg** flat (DL1), or **60 mg** flat (DL2) will be taken orally by the patient on D1, D8 and D15 of each cycle).<br>Dose escalation will begin at DL1 and will continue until the MTD is exceeded or until the highest dose level defined in the study (DL2) is reached.<br><br>**<u>Part 2: Expansion phase</u>**<br>The dose escalation phase will be followed by an exploratory expansion phase in group B bis depending on the decision of the Independent Data Monitoring Committee (IDMC) after review of safety data at the end of dose

	escalation part.<br>Patients enrolled in the expansion phase will receive selinexor at the RP2D defined by the IDMC, with the conventional regimen R-GDP.<br><br>For both parts of the study, there will be 3 periods:<br>• <u>Baseline period</u>: within 28 days before initiation of the study treatment (C1D1).<br>• <u>Treatment period</u> of 3 cycles (3-week cycles) of selinexor and R-DHAOx / R-GDP.<br>The occurrence of DLTs will be monitored during cycle 1 in the dose escalation phase only.<br>After 3 cycles of treatment, all patients will be evaluated for response (End of Treatment EoT evaluation) in the 10 days following the end of cycle 3. Patients who achieve adequate conditions allowing for ASCT may proceed to ASCT according to institutional protocols. **This EoT evaluation must always be done before the start of any alternate anti-lymphoma therapy or before the high-dose therapy (HDT) and ASCT procedure.**<br>Stem cell mobilization and collection may be proposed at the time of hematologic recovery after cycle 2 or cycle 3 according to institutional guidelines. Stem cell mobilization, collection and engraftment are optional and are not part of study treatment but data will be collected for exploratory purpose, if performed.<br>Permanent treatment discontinuation is defined as stopping treatment (immunochemotherapy and selinexor) before 3 cycles are completed for any reason. These patients must undergo permanent treatment discontinuation (PTD) evaluations within 4 weeks after the last drug administration.<br>If only selinexor administration is stopped due to toxicities, it will not be considered as permanent treatment discontinuation and the immunochemotherapy administration should continue.<br>• <u>Post-treatment follow-up period</u>: every 3 months during the first year, then every 6 months until the end of the study. During the follow-up period, disease status, adverse events according to adverse events reporting rules, survival status, and completion of high-dose chemotherapy (HDT)/ASCT or subsequent anti-lymphoma therapy will be collected.
**Duration of the study**	- Estimated date for First Patient In (FPI): September 2016.<br>- *Estimated recruitment period*:<br>• Escalation part: 2.5 years<br>• Expansion part: 1 year<br>- *Treatment period (both parts of study)*: Each patient will be treated during about 3 months (complete treatment: 3 cycles of 21 days followed by the EoT evaluation performed in the 10 days following the end of cycle 3)<br>- *Follow-up period*: Each patient will be followed for a minimum of 6 months after the EoT (or permanent treatment discontinuation, PTD) and until the

	end of study.<br>The study will end 6 months after the last patient has completed the EoT/PTD visit.<br>**The total duration of the study is thus estimated to be approximately 4 years (including patient's' follow-up).**
**Number of patients**	A minimum of 23 patients and a maximum of 39 patients will be enrolled in this study (total for the 2 parts of the study):<br>- Dose escalation part: 27 patients<br>o Group A: 7 patients were included<br>o Group B: 7 patients were included<br>o Group B bis:<br>▪ 6 patients were included at DL1<br>▪ 7 patients were included at DL2<br>- Expansion phase: 12 patients in group B bis
**Inclusion criteria**	**Patients must satisfy all of the following criteria to be enrolled in the study in group B bis:**<br>1. Patients with any type of relapsed or refractory B-cell lymphoma<br>2. Eligible to receive R-GDP regarding the investigator's opinion<br>3. Who received prior therapy with at least one but no more than two lines therapies for B-Cell Lymphoma<br>4. Patient must have measurable disease defined by at least one single node or tumor lesion > 1.5 cm<br>5. Aged between 18 years and 70 years (included) on date of consent signature<br>6. Eastern Cooperative Oncology Group (ECOG) performance status of 0, 1 or 2<br>7. With a life expectancy of ≥ 3 months<br>8. Having signed a written informed consent<br>9. Male patients (if sexually active with a woman of childbearing potential*) must agree to use a reliable method of birth control (see **section 8.4.2.2** of the protocol) during the study treatment and for at least 12 months after the last study drug administration. Male patients must agree to not donate sperm during the study treatment and for at least 12 months after the last study drug administration<br>10. Female patients of childbearing potential* must agree to use two reliable methods of birth control during study treatment and for 12 months after the last dose and have a negative serum human chorionic gonadotropin (hCG) pregnancy test within 3 days prior to C1D1. Reliable methods of contraception include intrauterine devices, hormonal contraceptives [contraceptive pills, implants, transdermal patches, hormonal vaginal devices or injections with prolonged release], abstinence or sterilization of the partner (see **section 8.4.2.2**).<br>* A female patient or woman is considered to have childbearing potential unless she meets at least one of the following criteria :

	→ Age > 50 years and naturally amenorrhoeic for > 1 year (amenorrhoea following cancer therapy does not rule out childbearing potential)<br>→ Previous bilateral salpingo-oophorectomy, or hysterectomy
**Exclusion criteria**	**Presence of any of the following will exclude a patient from enrollment in group B bis**:<br>1. Previous treatment with Selinexor<br>2. Known central nervous system or meningeal involvement by lymphoma<br>3. Contraindication to any drug contained in these regimen<br>4. Subjects with known Human Immunodeficiency Virus (HIV) positivity<br>5. Subjects with known active hepatitis B infection (positive Ag HBs) or positive serology to hepatitis B (Ag HBs or antibody anti-HBc or positive DNA PCR) or active hepatitis C infection (patients with positive HCV serology are eligible only if PCR is negative for known HCV RNA)<br>6. Subjects with any uncontrolled active systemic infection requiring intravenous (IV) antibiotics<br>7. Any of the following laboratory abnormalities within 14 days prior to first administration (C1D1) of study treatment:<br>a. Absolute neutrophil count (ANC) < 1,000 cells/mm3 (1.0 x 10^9/L)<br>b. Spontaneous (within 7 days of any platelet transfusion) platelet count < 100,000/mm3 (75 x 10^9/L if due to lymphoma)<br>c. Serum SGOT/AST or SGPT/ALT > 5.0 x upper limit of normal (ULN)<br>d. Serum total bilirubin > 2x Upper Limit of Normal (ULN), or > 5x ULN if due to Gilbert syndrom or lymphoma involvement<br>8. Creatinine clearance < 70 mL/min (for GDP)<br>9. Subjects with pre-existing ≥ Grade 2 neuropathy<br>10. Prior history of malignancies other than lymphoma (except for basal cell or squamous cell carcinoma of the skin or *in situ* carcinoma of the cervix or breast) unless the subject has been free of the disease for ≥ 3 years<br>11. Any life-threatening illness, serious active disease or co-morbid medical condition, laboratory abnormality, organ system dysfunction or psychiatric illness which, in the investigator's opinion, could compromise the patient's safety, interfere with the absorption or metabolism of selinexor, or put the study outcomes at undue risk, or that would prevent the subject from signing the informed consent form<br>12. Pregnant or breastfeeding women<br>13. Use of any standard or experimental anti-cancer drug therapy within 28 days of the initiation (Day 1) of study drug therapy (administration of glucocorticoïds should not exceed 1mg/kg/day in the 14 days prior to C1D1)<br>14. Patients with a circulating lymphocytes > 50,000/ mm3(50G/L)

Study medication and dose escalation rules	

Study medication and dose escalation rules

Eligible patients enrolled in the study will receive oral selinexor, together with a conventional immunochemotherapy regimen R-GDP (Group B bis).

During both escalation and expansion phases, ImmunochemotherapyR-GDP immunochemotherapy regimen will be administered for 3 cycles at 21 days interval, according to sites' local practice (their administration scheme, dose and route are provided in Table 1 for information purposes only).

Selinexor will be taken orally by the patient for 3 cycles (3-week cycles) Selinexor is to be taken with, or within 30 minutes of, solid food consumption together with at least 120 mL of fluids (water, milk, etc.).

Table 1: R-GDP and new Selinexor treatment schedule.

		Drug	Route	Dose	Dose Level	3-week cycle (21 days) Week 1 D 1	D 2	D 3	D 4	Week 2 D 8	D 10	Week 3 D 15
Group B bis	**R-GDP** 3 cycles at 21 days interval	Rituximab	IV	375 mg/m²	-	X						
		Dexamethasone	IV or PO	mg	-	40	40	40	40			
		Cisplatin	IV	75 mg/m²	-	X						
		Gemcitabine	IV	1000mg/m²	-	X				X		
	SELINEXOR	**SELINEXOR** **<u>Group B Bis</u>** On week 1, week 2 and week 3	**PO**	**40 mg**	**1# bis**	**X**				**X**		**X**
				**60 mg**	**2 bis**	**X**				**X**		**X**

During dose escalation phase for B bis , different dose levels for selinexor administration will be examined sequentially in each study group (see Table 2):

- ***Dose Level 1 (DL1, <u>starting DL</u>)*****:** 3 doses of selinexor at **40 mg** flat per 3-week cycle will be taken by the patient on D1, D8 and D15 of each cycle
- ***Dose Level 2 (DL2)*****:** 3 doses of selinexor at **60 mg** flat per 3-week cycle will be taken by the patient on D1, D8 and D15 of each cycle

Table 2: Dose levels of selinexor defined for this second dose escalation phase

Dose level	SELINEXOR Flat dose (PO)	Days of administration per 3-week cycle	Number of patients
DL1	40 mg	D1, D8, D15	3-6 / group
DL2	60 mg	D1, D8, D15	3-6 / group

During dose expansion phase, selinexor will be administered according to the RP2D defined by the IDMC at the end of dose escalation phase.

Treatment duration :

Each cycle comprises 21 days and begins with the day 1 of the immunochemotherapy. The cycle 1 day 1 should start at the latest one week after the date of inclusion. Each patient is planned to receive 3 cycles.

Permanent treatment discontinuation is defined as stopping treatment before 3 cycles are completed for any reason. If only selinexor administration is stopped due to toxicities, it will not be considered as permanent treatment discontinuation and the immunochemotherapy administration should continue.

Dose escalation rules:

Patients enrolled in the dose escalation phase will receive R-GDP, with increasing doses of selinexor, depending on the dose level at which they will be enrolled. Dose levels of selinexor defined for this study are detailed in Table 2.

Dose escalation will be performed in Group **B bis** (R-GDP) using a traditional 3+3 design.

Dose escalation will begin at DL1 with the enrolment of an initial cohort of 3 patients at this dose level.

Dose escalation to the next planned dose level will be decided by the SRC based on the rules described in Table 3, depending on the number of DLTs observed during the DLT assessment period (refer to section 8.3.2 for definition of DLT and DLT assessment period).

The maximum tolerated dose (MTD), assessed during Cycle 1 (DLT assessment period)), is defined as the highest dose level of selinexor in each group at which no more than 1 out of 6 patients experiences DLT.

Table 3: Escalation rules for selinexor escalation phase

Number of patients with DLT at a given dose level	Dose escalation rules
0/3 patients	Escalation to the next dose level will be decided.
1/3 patients	Another 3 patients will be enrolled at the same dose level:<br>- If 0 of the 3 new patients experiences DLT (for a total of 1/6 patients with a DLT at this dose level), escalation to the next dose level will be decided.

	– If ≥1 of the 3 new patients experiences DLT (for a total of ≥2/6 patients with a DLT at this dose level), then MTD is exceeded and the dose escalation phase of the trial will be terminated. The dose level directly below the current dose level will be considered the MTD. An additional cohort of 3 patients will be enrolled at the MTD (if only 3 patients were treated at this dose level) to reach a total of 6 patients treated at this dose level, in order to confirm the MTD.
≥ 2/3 patients	MTD is exceeded and the dose escalation phase of the trial will be terminated. The dose level directly below the current dose level will be considered the MTD. An additional cohort of 3 patients will be enrolled at this previous dose level (if only 3 patients were treated at that dose) to reach a total of 6 patients treated at this dose level, in order to confirm the MTD.

Selinexor escalation will continue until the highest dose level to be tested (DL2) is reached or the MTD is exceeded. If no DLT is observed during the DLT assessment period (0/3 patients or ≤1/6 patients experiences DLT) at DL2, then DL2 will be considered the MTD.

The RP2D for the expansion phase will be the MTD, or the highest tolerable dose based on the analysis of adverse events and serious adverse events outside of the DLT assessment period.

<u>DLT definition</u> :

The DLT assessment period begins with the first dose of selinexor and ends immediately prior to the initiation of the second cycle (for a maximum of 35 days after C1D1).

Only toxicities occurring during the DLT period will be used for the purposes of defining MTD. However, toxicities that occur in all cycles could be considered in the overall decisions of the SRC and IDMC. Patients considered non-evaluable for DLT may be replaced, but the safety profile of these patients will be included in the SRC review. Patients considered non-evaluable for DLT are patients who do not complete Cycle 1 for reasons other than toxicity. Patients with a complete cycle 1 are patients who received immunochemotherapies and all planned doses of selinexor during Cycle 1, unless the missed doses were due to study drug related AE(s). The SRC will also review any other toxicities that do not meet the below definitions and assess for DLT determination.

A DLT is defined as any of the following events (toxicity assessed using NCI CTCAE v4.03) that is assessed as related to selinexor or to the combination of selinexor with immunochemotherapies :

	– Any grade ≥ 3 non hematological toxicity, irrespective of the duration, with the following exceptions:<br>o Grade ≥ 3 diarrhea, anorexia/weight loss or nausea/vomiting will be considered as DLT only if lasting more than 7 days (despite optimal standard supportive care) or if such AE is leading to skipping 2 doses of selinexor<br>o Grade ≥ 3 fatigue/asthenia will be considered as DLT only if lasting more than 7 days or if such AE is leading to skipping 2 doses of selinexor<br>o Electrolyte abnormalities correctable with supportive therapy<br>– Any grade 4 hematological toxicity (except lymphocyte count decreased/lymphopenia) lasting more than 7 days or leading to skipping 2 doses of selinexor<br>– Any toxicity resulting in a delay of > 14 days of the initiation of the second cycle<br>Alopecia (of any grade) will not be considered as a DLT.<br><br>**Treatment modifications (refer to section 8.3.3)**<br>**Cycle delay is preferred over reduction of dose** for recovery of hematologic and non-hematologic toxicity.<br>– *Cycle delay*<br>After completion of each 21-day cycle, the start of a new cycle should be delayed for a maximum of 2 weeks in case of toxicity necessitating a delay.<br>In case of toxicity occurrence, temporary interruption/reduction of selinexor dose is allowed as specified in section 8.3.3.2 of the protocol.<br>**During dose escalation phase, dose modifications of selinexor are not allowed during Cycle 1, except in the case of a DLT occurrence.**<br>**During expansion phase, dose modifications of selinexor are allowed whatever cycle according to protocol's rules.**<br><br>– *Dose modifications for R-GDP*<br>During the study conduct, **R-GDP** immunochemotherapy regimen could be modified during the **cycle 1** even during the dose escalation part. Dose modification will be done **only for Gemcitabine** and **could be set up at Cycle 1 Day 8** based on hematological toxicities occurred, as specified in section 8.3.3.3.<br>**And in case of DLT, R-GDP** immunochemotherapy regimen **will be modified irrespectively of the treatment cycle.**<br>Dose modifications (25% dose reduction for each compound, respectively) are advised according to the levels of toxicities observed:<br>• Grade ≥ 2 neuropathy (cisplatin)<br>• Grade ≥ 2 creatinin elevation (cisplatin)<br>In case of worsening of creatinine clearance, cisplatin could be switched to

	carboplatin according to following guidelines: creatinine clearance <50ml/min, carboplatin AUC 4.<br><br>**<u>Selinexor Supportive Care, Contraception Requirements, and Concomitant Medications (refer to section 8.4)</u>**<br><br>The following concomitant medications taken by the patient from the date of inclusion and up to 30 days after the last dose of study drug (or beyond, if treatment taken for SAE/AE management) must be reported in the study eCRF:<br>o Administration of glucocorticoïds<br>o G-CSF received (number of days)<br>o All therapeutic blood component therapy administered (packed cells or platelet transfusion)<br><br>Any concomitant treatment or therapy used to treat a reported AE or SAE, regardless of the time of occurrence<br><br>*<u>Required 5-HT3 Antagonists</u>*<br><br>In order to minimize nausea, unless contraindicated, all patients should receive 5-HT3 antagonists (e.g ondansetron 8 mg or equivalent), starting on C1D1 before the first dose of selinexor and continued 2-3 times daily thereafter, as needed. Alternative treatment may be provided if the patient does not tolerate 5-HT3 antagonists.<br><br>*<u>Supportive Care</u>*<br><br>In addition to the required prophylactic therapy with 5-HT3 antagonists, supportive care including additional anti-nausea/anti-emetic therapy such as neurokinin (nk1) receptor antagonist (e.g aprepitant), acid suppression (proton-pump inhibitors and/or H2-blockers) and other treatments may be administered per institutional guidelines.<br><br>*<u>Infection</u>*<br><br>Patients can receive prophylactic antibiotics (sulfomethoxazole/trimethoprim) and antiviral therapy (valacyclovir, acyclovir or valgancyclovir) according to site local practice.<br><br>Appropriate broad-spectrum intravenous antibiotics and antifungal agents should be started immediately in patients who develop fever or other signs of systemic infection. Selinexor should be suspended in any patient with uncontrolled or suspected infections until the condition is stabilized. Selinexor can then be re-started at the same dose. See also Table 4.<br><br>*<u>Contraception Requirements</u>*<br><br>Female patients of child bearing potential must agree to use dual methods of contraception and have a negative serum pregnancy test at Screening; male patients must use an effective barrier method of contraception if sexually active with a female of child bearing potential.<br><br>The use of two forms of contraception are required, including one highly effective and one effective method of contraception (see Section 9.4.2.2).

	Non-Study drug Related Concomitant Medication and Treatment<br>Concomitant medications include any prescription or over-the-counter preparation, including vitamins, dietary supplements, over-the-counter medications, and oral herbal preparations taken during the study. Medications to treat concomitant diseases like diabetes, hypertension, etc., are allowed. Patients may continue their baseline medication(s).<br>Patients should also receive tumor lysis prophylaxis: If TLS risk factors are identified provide prophylactic IV hydration, regular monitoring of renal function and, if appropriate, administration of hypouricemic agents as you would to reduce the risk of TLS. Per protocol, all patients should maintain normal hydration status and caloric intake<br>Administration of glucocorticoids is allowed during the study but should not exceed 1 mg/kg/day in the 14 days prior to C1D1.<br>*Use of Blood Products*<br>During treatment, patients may receive red blood cell (RBC) or platelet transfusions, if clinically indicated, per institutional guidelines. Patients who require repeated transfusion support should be discussed with the Sponsor.<br>Appropriate anti-coagulation is allowed during the study (e.g., low molecular weight heparin, direct factor Xa inhibitors, etc.). Warfarin is allowed during the study provided patients are monitored for INR twice a week during the first two cycles of therapy, then weekly to biweekly thereafter.<br>Patients may receive supportive care with erythropoietin, darbepoetin, granulocyte-colony stimulating factor (G-CSF), pegylated growth factors, and platelet stimulatory factors, in accordance with clinical practice or institutional guidelines prior to entry and throughout the study.<br>*Restrictions for selinexor*<br>**Medications:** There are no restrictions on the use of acetaminophen or acetaminophen-containing products in combination with selinexor, EXCEPT on days of selinexor dosing, when acetaminophen use must not exceed a total daily dose of 1 gram.<br>Patients should not take GSH-, S-adenosylmethionine (SAM)-, or N-acetylcysteine (NAC)-containing products during their participation in this study as these products may enhance the metabolism of selinexor (refer to appendix 14 for the list of concerned products). However, they are permitted if the patient has elevated liver function tests. Patients should review all concomitant medication with the Investigator to avoid the use of these products.<br>Patients should be carefully instructed to avoid the use of drugs or dietary supplements containing glutathione, S-adenosylmethionine, N-acetylcysteine, or high-dose vitamin C (which may affect the metabolism of glutathione) during their treatment, as they may lower selinexor concentration.<br>**Diet:** There are no dietary restrictions on this study. Patients on selinexor should maintain adequate caloric and fluid intake.

	**Prohibited Medications:** Concurrent therapy with any approved or investigative anticancer therapeutic (including radiotherapy) outside of those included in this study is not allowed. Use of any immunosuppressive agents during the study must be approved by the Sponsor prior to use. Refer to the full prescribing information of drugs composing R-GDP regimen or the most current information on prohibited concurrent medications.<br>Treatment which could increase Selinexor exposition are forbidden.<br>**Stem cell mobilisation and collection (not part of the study):**<br>Stem cell mobilization and collection can be proposed by investigators, but only after the completion of at least cycle 2. Conditionning regimen will be administered according to institutional guidelines. G-CSF (or PEG-G-CSF) may be given from Day 6 (Day 3 for PEG-G-CSF) for 7 days or until neutrophil recovery (ANC ≥ 1000/mm^3) or according to each centre procedure for stem cell mobilization.
**Assessment schedule**	**Treatment period**<br>Physical examinations (including body weight, BSA, performance status) will be obtained prior to drug administration, and before each cycle of treatment, and up to 30 days after the last drug administration.<br>Hematology lab tests with White Blood Cell (WBC) differential and serum chemistries will be obtained at baseline prior to drug administration, then at least at day 1, day 10 and between day 14 and day 21 of each cycle during the 3 cycles of study treatment, and up to 30 days after the last drug administration.<br>**The following parameters must be met on Day 1 of each cycle for the initiation of a new cycle:**<br>- ANC ≥ 1.0 x 10^9/L<br>- Platelet count ≥ 75 x 10^9/L<br>- Non hematologic toxicities of grade ≥ 3 must have recovered to baseline (unless authorization is given by the sponsor)<br>In case of inadequate laboratory values, initiation of a cycle can be delayed, as described in section 8.3.3.1.<br><br>AEs/SAEs type, severity (according NCI-CTCAE V4.03), cycle of occurrence, duration, seriousness, and relationship to study treatment will be assessed up to 30 days after the last drug administration (see sections 13.2, 13.3 and 13.4 for reporting rules). Laboratory abnormalities will be assessed according to the NCI-CTCAE V4.03.<br><br>Tumour assessment (clinical examination, laboratory tests, abdominal and chest CT scan, PET scan, bone marrow biopsy) will be performed at baseline, and in the 10 days after the end of cycle 3 (EoT evaluation). Any patient withdrawing from the study should be assessed within 4 weeks after the last treatment dose for efficacy and toxicities (EoT evaluation).<br>Patients who achieve adequate conditions allowing for autologous stem cell

	transplantation (ASCT) may proceed to ASCT according to institutional protocols.<br>**This EoT evaluation must always be done before the start of any alternate anti-lymphoma therapy or before the high-dose therapy and ASCT procedure.**<br>To ensure comparability, baseline and on-study methods for response assessment will be performed using identical techniques. Response criteria will be assessed using International (Lugano) 2014 criteria.<br>**<u>Post-treatment follow-up period</u>**<br>After the completion of the 3 cycles (or permanent discontinuation) of study treatment, patients will be followed every 3 months during the first year, then every 6 months until the end of the study. During the follow-up period, disease status, adverse events according to adverse events reporting rules, survival status, and completion of high-dose chemotherapy (HDT)/ASCT or subsequent anti-lymphoma therapy will be collected.
**Statistical consideration**	**<u>Sample size calculation</u>**<br>No formal power calculations were performed to pre-determine sample size. The total number of patients enrolled will depend on the outcome of the actual dose escalation process.<br>Seven patients were included in both groups (A and B) in the escalation phase. Three patients were included in group B bis at DL1. Up to 9 patients may be enrolled in the study if dose escalation of selinexor continues up to DL2 in group B bis (R-GDP).<br>For the dose escalation, a minimum of 6 patients and a maximum of 9 patients will be enrolled in group B bis.<br>For the expansion phase, 12 patients will be enrolled at the RP2D in group B bis.<br>Therefore, a minimum of 23 patients and a maximum of 38 patients (or 26 if expansion phase cannot be performed) will be enrolled in this study.<br><br>**<u>Analysis plan</u>**<br><br>*<u>Dose escalation phase :</u>*<br>The primary objective of the study is to determine the RP2D for selinexor in patients with relapsed or refractory B-cell malignancies receiving either R-DHAOx or R-GDP, by assessing the maximum tolerated dose (MTD) observed during the dose escalation part of the study. Assessment of the MTD will be performed by the analysis of the dose-limiting toxicities (DLTs). MTD and RP2D for selinexor will be assessed independently in each group (Group A, B and B bis).<br>During the escalation phase, DLTs and all available safety data will be reviewed on an on-going basis by the Safety Review Committee (SRC, composed by at least one investigator from each site which included a patient in the concerned cohort, the coordinating or co-coordinating Investigator, the Sponsor's safety officer, the Sponsor's statistician and the

	Sponsor's clinical project manager of the study) and summarized in a conclusion for each dose level. The opening of next cohort at a given dose level will be based on this review by the SRC. Complete safety data for the escalation will be summarized at the completion of escalation phase for review by an Independent Data Monitoring Committee (IDMC).<br><br>*<u>Expansion phase:</u>*<br>Group B bis will be analyzed for efficacy and safety.<br>*Efficacy* will be analyzed when all patients of the expansion phase have completed 3 cycles or permanently discontinued treatment prior to the end of cycle 3:<br>– Response rate and Overall response rate according to Lugano 2014 classification criteria<br>- Survival endpoints (DoR, PFS, TTNLT and OS)<br>*Safety* will be analyzed when all patients of the expansion phase have completed 3 cycles or permanently discontinued treatment prior to the end of cycle 3:<br>– Grade ≥ 3 toxicities will be displayed<br>– Grade ≥ 2 renal and neuropathic toxicities will be displayed<br><br>**<u>Time of planned analyses</u>**<br><br>The study design involves 2 different analyses:<br><br>→ *<u>Primary endpoint analysis</u>*<br>The first clinical data cutoff for the primary endpoint analysis will occur after the last patient in the dose escalation phase completes Cycle 3. The aim of this analysis is to identify the maximum tolerated dose (MTD) after 1 cycle of treatment (safety analysis only for all patients) in each group.<br>The primary endpoint analysis will be performed once when the group (B bis) achieved the cut off timepoint. Each group (A, B and B bis) will be analyzed by cohort and dose level.<br>Efficacy and safety analysis based on the 3 cycles after the inclusion of the last patient in the escalation phase will also be performed for each group by dose level and in aggregate (A +B+Bbis).<br><br>→ *Final analysis*<br>The second data cutoff for the final analysis will occur 6 months after the last patient on study has completed the EoT/PTD evaluation.<br>Efficacy and safety analysis based on the 3 cycles after the inclusion of the last patient in the dose escalation/expansion cohorts will be performed.<br>The final analysis will be performed once when the two groups achieved the cutoff timepoint on patients having received the RP2D. Each group will be analyzed in aggregate as well as independently.

1 TABLE OF CONTENTS

SYNOPSIS 3

1 TABLE OF CONTENTS 17

2 LIST OF ABBREVIATIONS AND GLOSSARY OF TERMS 21

3 RESPONSIBILITIES 23
3.1 Sponsor and program coordination site 23
3.1.1 Sponsor 23
3.1.2 Coordinating investigators 23
3.1.3 Program coordination center 23
3.1.4 Biology, anatomopathology and imaging referents 24
3.2 Investigators 24
3.3 Laboratory sites 24

4 BACKGROUND AND STUDY RATIONALE 25
4.1 B-cell lymphoma 25
4.1.1 Treatment options for relapsed/refractory DLBCL 25
4.1.2 Choice of conventional multiple-drug second-line chemotherapies 25
4.2 Selinexor (KPT-330) 26
4.2.1 Introduction and mechanism of action 26
4.2.2 Non clinical studies 27
4.2.3 Clinical studies 27
4.3 Rationale for conducting this study 28
4.3.1 Selinexor in NHL 28
4.3.2 Rationale to administer Selinexor in patients receiving R-DHAOx/R-GDP 29
4.3.3 Rationale for the second dose esclation 31
4.3.4 Rationale for closing the R-DHAOx treatment 31

5 STUDY OBJECTIVES 31
5.1 Primary Objective 31
5.2 Secondary objectives 32
5.3 Exploratory objectives 32

6 STUDY DESIGN 32
6.1 General overview 32
6.2 Anticipated study timelines 34

7 STUDY POPULATION 35
7.1 Inclusion criteria 35
7.2 Exclusion criteria 36

8 TREATMENTS 37
8.1 Administration of conventional immunochemotherapy regimens 37
8.2 Administration of Investigational Medical Product (IMP): Selinexor 37
8.2.1 Description, dosage, packaging and labeling 37
8.2.2 Storage conditions and stability 37
8.2.3 Handlings 37
8.2.4 Dosage and administration of selinexor 38
8.2.5 Missed or vomited doses of selinexor 38

8.2.6 Overdose of selinexor ... 38
8.2.7 Initial drug supply and ordering of re-supply ... 38
8.3 Treatment schedule and duration ... 39
8.3.1 Escalation rules for selinexor during dose escalation phase ... 40
8.3.2 DLT definition and DLT assessment period (dose escalation phase) ... 41
8.3.3 Treatment modifications ... 42
8.4 Selinexor Supportive Care, Contraception Requirements, and Concomitant Medications ... 47
8.4.1 Required 5-HT3 Antagonists ... 47
8.4.2 Supportive Care ... 48
8.4.3 Non-study drug Related Concomitant Medication and Treatment ... 49
8.4.4 Restrictions for Selinexor ... 50
8.4.5 Prohibited Medications ... 50
8.4.6 Stem cell mobilisation and collection (not part of the study) ... 50
8.5 Drug Dispensation and accountability ... 51
8.5.1 Responsibilities ... 51
8.5.2 Accountability and compliance ... 51
8.5.3 Destruction ... 51

9 STUDY FLOW CHART AND SCHEDULE OF ASSESSMENTS ... 52
9.1 Study flow chart ... 52
9.2 Baseline evaluations (Day -28 to Day -1) ... 52
9.3 Evaluations during treatment period ... 54
9.4 End of treatment (EoT) / permanent treatment discontinuation (PTD) evaluations ... 55
9.5 Post-treatment follow-up assessments ... 56
9.6 Progression/relapse ... 56

10 STUDY PROCEDURES ... 57
10.1 Informed consent ... 57
10.2 Registration and inclusion procedure ... 57
10.3 Screen failure ... 57
10.4 Pathological diagnosis ... 58
10.5 PET scan upload ... 58
10.6 Blood sampling for biological studies ... 59

11 STUDY COMMITTEES ... 59
11.1 Safety review Committee (SRC) ... 59
11.2 Independent Data Monitoring Committee (IDMC) ... 60

12 CRITERIA FOR PREMATURE DISCONTINUATION OF THE STUDY ... 60
12.1 Permanent treatment discontinuation (PTD) ... 60
12.2 Withdrawal of Consent ... 61
12.3 Patients Lost to Follow up ... 61
12.4 Premature discontinuation of the study ... 61

13 SAFETY PARAMETERS ... 62
13.1 Definitions ... 62
13.1.1 Adverse Events ... 62
13.1.2 Serious Adverse Events ... 62
13.1.3 Adverse Event Severity/Intensity ... 63

13.2 Adverse Events reporting rules 63
13.3 Serious Adverse Events reporting rules 64
13.3.1 Emergency outpatient treatment or observation that does not result in admission, unless fulfilling other seriousness criteria above (section 13.1.2) Obligations of the investigator 65
13.3.2 Obligations of the Sponsor 66
13.4 Reporting of Adverse Events of special interest 66
13.4.1 Expedited reporting of cerebellar toxicities 66
13.4.2 Reporting of Cataracts 67
13.4.3 Tumor lysis Syndrome 67
13.5 Suspected Unexpected Serious Adverse Reactions 67
13.6 Follow up of AEs and SAEs 67
13.7 Procedures for Handling Special Situations 68
13.7.1 Pregnancy 68
13.7.2 Male patients 68
13.7.3 Overdose 69

14 GENERAL STATISTICAL CONSIDERATIONS 70
14.1 Study design 70
14.2 Study Objectives 70
14.2.1 Primary Objective 70
14.2.2 Secondary Objectives 70
14.2.3 Exploratory Objectives 70
14.3 Endpoints 71
14.3.1 Primary endpoint 71
14.3.2 Secondary endpoints 71
14.3.3 Exploratory endpoints 72
14.4 Analysis sets 72
14.4.1 DLT Set 72
14.4.2 Safety Set 72
14.4.3 Efficacy Set 72
14.5 Statistical methods 73
14.5.1 Patients replacement 73
14.5.2 General Approach 73
14.5.3 Efficacy Analysis 73
14.5.4 Safety Analysis 73
14.6 Sample size 73
14.7 Planned analyses 74
14.7.1 Primary endpoint analysis (Interim analysis) 74
14.7.2 Final analysis 74

15 STUDY MONITORING 74
15.1 Responsibilities of investigators 74
15.2 Responsibilities of the sponsor 74
15.3 Use and completion of electronic case report form (eCRF) 75

16 ETHICAL AND REGULATORY STANDARDS 75
16.1 Ethical principles 75
16.2 Laws and regulations 75

16.3 Informed consent ... 75
16.4 Ethics Review Committee and Competent Authorities submission ... 76

17 ADMINISTRATIVE PROCEDURES ... 76
17.1 Curriculum vitae ... 76
17.2 Confidentiality agreement ... 76
17.3 Record retention in investigating site(s) ... 76
17.4 Ownership of data and use of the study results ... 77
17.5 Publication ... 77
17.6 Insurance compensation ... 77
17.7 Company audits and inspections by regulatory agencies ... 77
17.8 Clinical study report ... 78
17.9 Protocol amendments ... 78

18 REFERENCES ... 79

19 APPENDICES ... 82
19.1 Appendix 1: Study design ... 82
Appendix 2: Schedule of Evaluations (study flow-chart) ... 83
19.2 Appendix 3: Ann Arbor staging ... 85
19.3 Appendix 4: International Prognostic Index (IPI) and age-adjusted International Prognostic Index (aa-IPI) ... 86
19.4 Appendix 5: Follicular Lymphoma International Prognostic Index (FLIPI) ... 87
19.5 Appendix 6: MCL International Prognostic Index (MIPI) ... 88
19.6 Appendix 7: Performance Status Criteria ... 89
19.7 Appendix 8: Body Surface Area calculation ... 90
19.8 Appendix 9: Ophthalmic examination ... 91
19.9 Appendix 10: PET/CT SCANs ... 92
19.9.1 Timing of FDG PET scans ... 92
19.9.2 Patient preparation ... 92
19.9.3 PET scanner technical requirements ... 93
19.9.4 PET acquisition and reconstruction ... 93
19.9.5 PET Review LOGISTICS ... 93
19.10 Appendix 11: Recommendations for Initial Evaluation, Staging, and Response Assessment of Hodgkin and Non-Hodgkin Lymphoma : The Lugano Classification ... 94
19.11 Appendix 12: Pathological Samples Review ... 97
19.12 Appendix 13: Biological samples for further ancillary studies ... 98
19.13 Appendix 14: Representative list of the products containing GLUTATHIONE (GSH), S-ADENOSYLMETHIONINE (SAM) or N-ACETYLCYSTEINE (NAC) ... 100
19.14 Appendix 15 : Group B : R-GDP guidelines ... 102
19.15 Appendix 16 : Group B bis : R-GDP guidelines ... 103

2 LIST OF ABBREVIATIONS AND GLOSSARY OF TERMS

Abbreviation	Term
AE / AESI	Adverse Event / Adverse Event of Special Interest
ALT (SGPT)	ALanine Transaminase (Serum Glutamic Pyruvic Transaminase)
AML	Acute Myeloid Leukaemia
ANC	Absolute Neutrophil Count
ASCT	Autologous Stem Cell Transplantation
AST (SGOT)	ASpartate Transaminase (Serum Glutamic Oxaloacetic Transaminase)
β-HCG	beta-Human Chorionic Gonadotropin
BIW	Twice a week
BSA	Body Surface Area
C1D1	Day 1 of Cycle 1
CD20	antigen expressed on the surface of normal and malignant B lymphocytes
CORAL	Collaborative Trial in Relapsed Aggressive Lymphoma
CR	Complete Response
CrCl	Creatinine Clearance
CT	Computed Tomography
CTCAE	Common Terminology Criteria for Adverse Events
DCR	Disease Control Rate
DL	Dose Level
DLBCL	Diffuse Large B-Cell Lymphoma
DLT	Dose Limiting Toxicity
DNA	Deoxyribonucleic acid
DoR	Duration of Response
ECG	Electrocardiogram
ECOG	Eastern Cooperative Oncology Group
eCRF	Electronic Case Report Form
EDTA	Ethylenediaminetetraacetic acid
EoT	End of Treatment
FDG	Fluorodeoxyglucose (18F)
FL	Follicular cell Lymphoma
FLIPI	Follicular Lymphoma International Prognostic Index
GCB	Germinal Center B
GCP	Good Clinical Practice
G-CSF	Granulocyte Colony-Stimulating Factor
HBV	Hepatitis B Virus
HCV	Hepatitis C Virus
HDT	High Dose Therapy
HIV	Human Immunodeficiency Virus
ICH	International Conference on Harmonization
IDMC	Independant Data Monitoring Committee
IHC	Immunohistochemistry
IMP	Investigational Medical Product
INR	International Normalized Ratio

IPI / aa IPI	International Prognostic Index / age-adjusted International Prognostic Index
IRB	Institutional Review Board
IV	IntraVenous
IWG	International Working Group
LDH	Lactic DeHydrogenase
LYSA	The Lymphoma Study Association
LYSARC	The Lymphoma Academic Research Organisation
MCL	Mantle Cell Lymphoma
MIPI	Mantle Cell Lymphoma International Prognostic Index
MTD	Maximum Tolerated Dose
NCI	National Cancer Institute
NHL	Non-Hodgkin's Lymphoma
ORR	Overall Response Rate
OS	Overall Survival
PBSC	Peripheral Blood Stem Cell
PCR	Polymerase Chain Reaction
PD	Progressive Disease
PET	18F-FDG Positon Emission Tomography
PFS	Progression Free Survival
PO	Per os (oral)
PR	Partial Response
PS	Performance Status
PTD	Permanent Treatment Discontinuation
QW	Once a week
RBC	Red Blood Cells
R-DHAOx	Rituximab-Dexamethasone, high-dose Cytarabine-Oxaliplatin
R-DHAP	Rituximab-Dexamethasone-high-dose Cytarabine and Cisplatin
R-GDP	Rituximab-Gemcitabine-Dexamethasone and Cisplatine
RNA	Ribonucleic acid
RP2D	Recommended Phase II Dose
RPPS	Répertoire Partagé des Professionnels de Santé *(Health Professionals Shared Directory)*
SAE	Serious Adverse Event
SD	Stable Disease
SINE	Selective Inhibitor of Nuclear Export
SUSAR	Suspected Unexpected Serious Adverse Reaction
SRC	Safety review Committee
TTNLT	Time To Next anti-Lymphoma Treatment
TSPs	Tumor Suppressor Proteins
ULN	Upper Limit of Normal
XPO1	Exportin-1
WBC	White Blood Cells
WHO	World Health Organization

3 RESPONSIBILITIES

3.1 Sponsor and program coordination site

3.1.1 Sponsor

LYSARC (the Lymphoma Academic Research Organisation)
🖂 : Centre Hospitalier Lyon Sud – Secteur Sainte Eugénie - Pavillon 6D
F-69495 Pierre Bénite Cedex - France
☎ : +33(0) 4 72 66 93 33 Fax : +33(0) 4 72 66 93 71
Email : selinda@lysarc.org

3.1.2 Coordinating investigators

Coordinating investigator :
Pr. Hervé TILLY
🖂 : Centre Henri Becquerel - Service Hématologie
Rue d'Amiens
76038 Rouen - France
☎ : +33(0) 2 32 08 22 02 Fax : +33(0) 2 32 08 22 83
Email : herve.tilly@chb.unicancer.fr

Co-coordinating investigator:
Dr. Marie MAEREVOET
🖂 : Institut Jules Bordet - Service Hématologie
121 chaussée de Waterloo
B 1000 Bruxelles - Belgium
☎ : +32 25 41 31 11 Fax : +32 25 44 02 57
Email : marie.maerevoet@bordet.be

3.1.3 Program coordination center

LYSARC (the Lymphoma Academic Research Organisation)
🖂 : Centre Hospitalier Lyon Sud – Secteur Sainte Eugénie - Pavillon 6D
F-69495 Pierre Bénite Cedex - France
☎ : +33(0) 4 72 66 93 33 Fax : +33(0) 4 72 66 93 71

Project Management:
Chloé GOURC-BERTHOD, Clinical Project Manager
🖂 : LYSARC - Centre Hospitalier Lyon Sud – Secteur Sainte Eugénie - Pavillon 6E
F-69495 Pierre Bénite Cedex - France
☎ : +33(0)4 72 24 41 78 Fax : +33 (0) 4 72 66 38 57
Email : chloe.gourc-berthod@lysarc.org

Clinical operations (LYSARC):
Jérome Paget, Director of Biometrics Division (jerome.paget@lysarc.org)
Florence BROUSSAIS, Director of the Medical and Scientific division (florence.broussais@lysarc.org)
Delphine GERMAIN, Director of Project Management and Regulatory Affairs Division

(Delphine.germain@lysarc.org)
Anaïs EL HACHEMI-DUMAS, Head of Pharmacovigilance (anais.el-hachemi-dumas@lysarc.org)
Pharmacovigilance : Fax : +33 (0) 3 59 11 01 86 / email: pharmacovigilance@lysarc.org
Emmanuelle ROBERT-EYDOUX, Director of Monitoring and Site Management (emmanuelle.robert-eydoux@lysarc.org)

3.1.4 Biology, anatomopathology and imaging referents

Referent anatomopathologist:
Dr Vanessa SZABLEWSKI
Pr Christiane COPIE

Biology and anatomopathology coordinator:
Nadine VAILHEN, Director of Biological and Histopathological Operations, LYSA-P
✉ : CHU Henri Mondor - 51, av. du Maréchal de Lattre de Tassigny
94010 CRETEIL – France
☎ : +33(0) 1 49 81 37 48 Fax: +33(0) 1 49 81 37 49
Email : nadine.vailhen@lysarc.org

Imaging referent
Dr. Stéphanie BECKER
✉ : Centre Henri Becquerel - Service Hématologie
Rue d'Amiens
76038 Rouen - France
☎ : +33(0) 2 32 08 22 22 Fax: +33(0) 2 32 08 25 50
Email : stephanie.becker@chb.unicancer.fr

Imaging coordinator:
Romain RICCI, Imaging Coordinator (), LYSA-IM
✉ : CHU Henri Mondor - 51, av. du Maréchal de Lattre de Tassigny
94010 CRETEIL – France
☎ : +33(0) 1 49 81 47 86 (LYSA-IM) Fax: +33(0) 1 49 81 27 94 (LYSA-IM)
Email : romain.ricci@lysarc.org

3.2 Investigators

6 to 8 LYSA sites from France and Belgium during the dose escalation phase, then 10 to 15 LYSA sites from France and Belgium during the expansion phase (including sites of dose escalation phase) may include patients in this study. Before any inclusion, each site must be declared to the Ethical Committee and national competent authority according to each country regulations and have a study training delivered by the sponsor or its delegate (i.e. initiation visit/call). To be declared as a participating site, the principal investigator must send to LYSARC all administrative documents required for regulatory submission (e.g. Curriculum vitae, RPPS number for French investigators or registration number to local medical association, etc ...).

3.3 Laboratory sites

Laboratories of each study site must provide their normal values and an updated accreditation for quality control.

4 BACKGROUND AND STUDY RATIONALE

4.1 B-cell lymphoma

Non-Hodgkin lymphomas (NHLs) are of B-cells origin. They can be divided into 2 general prognostic groups: indolent lymphomas and aggressive lymphomas. The first have a good prognosis but are incurable in advanced stage. The latter can be cured in approximately 50% of the cases. In the Western World, Diffuse Large B Cells Lymphomas (DLBCL) comprise one-third of all adult NHL cases and follicular lymphoma (FL) is the second most frequent subtype comprising 22% of NHL. Mantle Cells lymphoma is much rarer, representing about of 6% of all B-cells lymphomas and is associated with a dismal outcome, only 3 to 5 years of median survival (1).

4.1.1 Treatment options for relapsed/refractory DLBCL

Despite overall improvements in outcome of diffuse large B-cell lymphoma (DLBCL), approximately one-third of patients are still refractory to rituximab-based therapies. Relapsed and refractory disease continues to represent the most significant challenge in treating Non Hodgkin Lymphoma (NHL).

Salvage chemotherapy followed by high-dose therapy (HDT) and autologous stem-cell transplantation (ASCT) is the standard treatment of young patients with relapsed diffuse large B-cell lymphoma (DLBCL) and can be cure a proportion of these patients (2).

The achievement of a complete remission before ASCT is the most important factor associated with a better outcome. The patients with an inadequate response to second-line chemotherapy are considered to have a poor recovery rate with subsequent salvage regimens, the prognosis of these patients is poor (less than 10% of 1 year survival).

4.1.2 Choice of conventional multiple-drug second-line chemotherapies

The Collaborative Trial in Relapsed Aggressive Lymphoma (CORAL) compared the patients with refractory or relapsed $CD20^+$ DLBCL who were randomly assigned to either Rituximab, Ifosfamide, Carboplatin, and Etoposide (R-ICE) or Rituximab, Dexamethasone, high-dose Cytarabine, and Cisplatin (R-DHAP). Patients who responded to the chemotherapy were submitted to HDT and ASCT (3). The results revealed no significant difference in outcome between the two regimens but a better response to R-DHAP in GCB-like DLBCL (4).

DHAP-like regimens with an alternate platinum compound, such as DHAOx (Dexamethasone, high-dose Cytarabine-Oxaliplatin) or DHAC (Dexamethasone, high-dose Cytarabine-Carboplatin) might be a preferred option in patients at risk for renal insufficiency. The recommended dose for oxaliplatin is 130 mg/m^2 (5).

In comparison with (R) DHAP, treatment with (R) GDP (Gemcitabine, Dexamethasone and Cisplatine) is associated with a noninferior response rate, similar transplantation rate, event-free survival, and overall survival, less toxicity and hospitalization, and superior quality of life (6).

4.2 **Selinexor (KPT-330)**

4.2.1 Introduction and mechanism of action

Shuttling proteins out of the nucleus is an essential component of the regulation of cell-cycle and proliferation both in the normal and neoplastic tissues. *XPO1* encodes for Exportin-1/XPO1 (also called CRM1), one of the principal mediators of Nuclear Export Signal (NES)-dependent protein transport. It has been known to regulate the retention of key disease tumour suppressor proteins (TSPs) and oncogenes, such as p53 (7) and many NF-κB target genes such as IKB/IKA complex members (8), HSCARG (9), RELA (p65) (10), COMMD1 (11) and many others (12-14). Overexpression of XPO1, *in vitro,* causes an increase export of TSPs and growth regulatory proteins from the nucleus. With TSPs outside the nucleus, cells can resist apoptosis and carry on dividing (15-18).
Selinexor is an orally bioavailable, reversible, highly potent, Selective Inhibitor of Nuclear Export (SINE). Mechanistic studies have shown that SINE compounds inhibit XPO1 mediated nuclear to cytoplasmic transport by transiently binding to XPO1 cargo binding site, thereby inducing nuclear localization and activation of multiple TSPs, along with reduction in oncoprotein levels (e.g., c-Myc and the anti-apoptotic protein BCL-X_L) leading to rapid apoptosis of multiple myeloma (MM) cells (*19*).

All cell types exposed to SINE compounds in vitro undergo G1 ± G2 cell cycle arrest, followed by a ‘genomic fidelity’ review. Cells with damaged genomes are induced to undergo apoptosis. Normal cells, with an intact genome, remain in transient, reversible cell cycle arrest until the nuclear export block is relieved.
Selinexor and other SINE compounds are not intrinsically cytotoxic; rather, they can restore the highly effective endogenous tumor suppressing pathways that lead to selective elimination of genomically damaged (i.e., neoplastic) cells.
Tumours of hematopoietic lineage are particularly susceptible to induction of apoptosis by XPO1 inhibition; normal hematopoietic cells and their functions are largely spared.

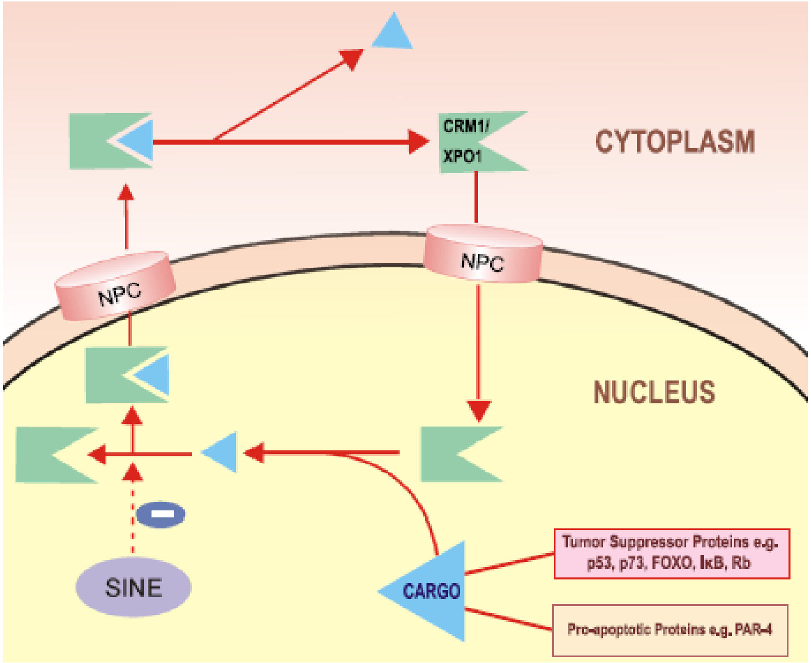

(From Parikh et al. Journal of Hematology & Oncology 2014, 7:78)

4.2.2 Non clinical studies

In vitro experiments with continuous (approximatively 72-hour) exposure to selinexor demonstrated potent pro-apoptotic activity across a broad panel of tumour-derived cell lines and patient samples in culture, including multidrug-resistant cancers. Selinexor and related SINE compounds have shown substantial efficacy with dosing regimens that match those currently under investigation in humans in a variety of mouse models of haematological and solid tumours, including DLBCL, MCL, non-Hodgkin-Lymphoma (20), Acute Myeloid Leukaemia AML (21), CLL, multiple myeloma (MM) (22), T-cell acute lymphoblastic leukaemia (T-ALL), neuroblastoma, melanoma, prostate, breast, lung, and ovarian cancer.

Pharmacokinetic studies conducted in mice, rats and monkeys showed dose proportional exposure for Selinexor with no accumulation.

Detailed non clinical data available on selinexor are summarized in the current selinexor/KPT-330 Investigator's Brochure.

4.2.3 Clinical studies

First-in-human Phase 1 studies with oral selinexor were conducted in advanced haematological malignancies including non-Hodgkin's lymphoma (NHL), chronic lymphocytic leukaemia (CLL), multiple myeloma (MM), and acute myeloid leukaemia (AML) (KCP-330-001), in solid tumours and in soft tissue and bone sarcomas (KCP-330-002 and -003). In addition, Phase 2 studies are underway in glioblastoma, gynaecological malignancies, squamous cell carcinoma, prostate cancer, acute myeloid leukaemia (AML), diffuse large B-cell lymphoma (DLBCL), and Richter's transformation. Over 1800 patients with hematologic or solid tumors have received at least one dose of selinexor to date.

4.2.3.1 Efficacy data in Human

Selinexor has shown single-agent, durable, anti-cancer activity in patients with multiple resistant/refractory (RR) hematologic and solid tumour malignancies, at doses of ≥ 6 mg/m^2 body surface area (BSA) in these initial Phase 1 dose-escalation studies.

In DLBCL, as of 01 June 2015, among 39 evaluable patients with heavily treated DLBCL the ORR was 31% including four CRs and eight PRs. For the 28 evaluable patients who were on study for ≥ 1 month, the DCR was 71% and the ORR 43%, and OS and PFS improved to 6.0 and 3.6 months, respectively, compared with overall OS and PFS of 4.6 and 1.7 months, respectively. There was a strong correlation between overall response and OS, with responding patients (N = 12 of 42) showing a median OS of greater than 10 months compared with patients with stable or progressive disease showing a median OS of 3.5 months. Interestingly, selinexor showed activity across both subtypes (Germinal centre (GC) or non-GC subtype).

4.2.3.2 Potential risks of Selinexor in Human

Selinexor is currently in clinical development and has not been approved by the Food and Drug Administration (FDA) for commercial use. Selinexor has been administered over 1800 patients to date, in both Karyopharm-sponsored and investigator-sponsored trials.

Clinical experience with selinexor has been evaluated in 1175 patients (as of the 31 May 2016 safety data analysis) and the full safety profile is not known at this time.

In ongoing clinical studies, the most common AEs suspected to be related to selinexor (incidences in parentheses) have been low-grade nausea (62%), fatigue (55%), anorexia

(50%), - and mild/moderate thrombocytopenia (43%) and vomiting 38%. Most of these effects can be managed effectively with dose modification and/or supportive care initiated prior to first dosing.
As of 31 May 2016, there are no known cumulative or major organ toxicities associated with selinexor, and several patients with heavily pretreated, relapsed/refractory cancers continue on single agent selinexor for >14 months, with the longest on for nearly two years.
In a previous study, one patient, heavily pre-treated for recurrent pancreatic cancer, developed acute cerebellar syndrome with dysarthria and ataxia following 3 doses of selinexor at 85 mg/m^2 BSA BIW. The patient experienced abnormal speech, loss of coordination, and was unable to walk. Since the date of the initial reported event, Selinexor was permanently discontinued, this patient is recovering, with both speech and mobility recovered to near baseline over ~6 weeks. No other patients have reported similar symptoms to date.

Overall, increased rates of infection associated with selinexor have not been reported; however, greater incidences of sepsis were seen with selinexor versus physician's choice in patients with relapsed/refractory AML who were > 60 years of age and receiving higher doses of selinexor (most patients were receiving 100 mg) in study KCP-330-008. This increased rate of sepsis has not been observed in any other studies conducted with selinexor to date.

Eight tumor lysis syndrome cases have been reported (as of May 2018), including 4 patients in Karyopharm-sponsored studies, 2 patients in Investigator-sponsored studies, and 2 patients in the expanded access program (compassionate use). Further details about these patients are provided in Section 13.4.3.

Please refer to the *Selinexor/KPT-330 IB* for the most current safety information.

Reproductive risks of selinexor

Macroscopic and microscopic changes in reproductive organs were noted during rat and monkey toxicology studies, most of which partially or fully resolved during the recovery period. The long-term effects of these changes on reproductive potential are unknown. Secondary developmental effects due to reduced maternal body weights were also noted during a study on rat embryo/fetal development. It is unknown whether similar effect may occur in humans. Please refer to the *Selinexor/KPT-330 IB* for additional information. As it is unknown whether selinexor might have reproductive toxicity in humans, patients must agree to use effective contraception (see Contraception Requirements in section 8.4.2.2) during the study and for 12 months after the end of treatment.

4.3 Rationale for conducting this study

4.3.1 Selinexor in NHL

Selective inhibitors of nuclear export (SINEs) are a novel class of antineoplastic drugs that specifically block XPO1, leading to the nuclear accumulation and re-activation of tumor suppressor proteins (TSPs) and other growth modulators. In addition, SINE inhibits XPO1

mediated mRNA nuclear export and translation of proteins with short half-lives, leading to down regulation of multiple tumor oncogenic proteins including c-Myc, Cyclin-D, Bcl-Xl and Bcl2/6.
Selinexor (KPT-330) is a new generation orally bioavailable SINE. Selinexor has shown single–agent durable anti-cancer activity with multiple relapsed/refractory hematologic malignancy (23).
Gutierrez *et al.* presented the findings of their phase I open label dose escalation trial of oral selinexor twice weekly conducted in patients with heavily pre-treated hematological malignancies (mainly myeloma, NHL and AML) in 32 pretreated refractory lymphoma patients who have had at least two but no more than four prior multi-agent therapies and are not eligible for high dose chemotherapy with stem cell rescue at the time of study entry. The optimal dosing of KPT-330 is at least 45 mg/m^2 and durable activity of KPT-330 was observed in those NHL patients (24). For DLBCL patients, 15 of the 21 patients (70%) evaluated as of 15 May 2014 have experienced a complete response (CR, one patient), partial response (PR, 5 patients) or stable disease (SD, 8 patients). Responses for the 21 patients who had been evaluated as of 15 May 2014, each of whom received a dose between 3 mg/m2 to 80 mg/m2 per cycle (patients on 80 mg/m2 twice weekly had their doses reduced to 70 mg/m2 twice weekly).

	N	DCR (%)	CR (%)	PR (%)	SD (%)	PD
**DLBCL**	21	15 (70%)	1 (5%)	5 (25%)	9 (40%)	5 (25%)

More recent results from this ongoing phase I were presented in 2015 (25) and suggest that while efficacy is comparable, doses of selinexor from 45-65 mg (median 60 mg) are better tolerated than doses >65 mg, based upon decreased weight loss, incidence of high grade AEs, and greater numbers of days on study. Based on this superior risk-benefit, a flat dose of 60 mg selinexor, twice weekly, is the recommended Phase II dose for patients with hematological cancers.

4.3.2 Rationale to administer Selinexor in patients receiving R-DHAOx/R-GDP

As the overall survival in the population refractory to the second line of chemotherapy is less than 1 year, a clear unmet medical need persists for patients with relapsed/refractory NHL, and particularly with DLBCL.
R-DHAP is a standard regimen used for relapsed B lymphomas (26). More recently, it has been replaced by R-DHAOx in France, due to the lower toxicity of oxaliplatin and the fact that frequent renal toxicity observed with cisplatin could hinder the intensification with HDT.
On the other hand, a randomized study demonstrated that R-GDP was less toxic than R-DHAP, with an equivalent efficacy (6).
These two associations, R-DHAOx and R-GDP, are now considered as reference salvage immunochemotherapies for relapsed/refractory B cell lymphomas.

Non clinical studies performed in multiple human cancer cell lines (from hematological malignancies or solid tumors) have shown a synergy effect between selinexor and various chemotherapy compounds (including dexamethasone, platins, cytarabin…) (section 1.3 of selinexor IB). Indeed, selinexor blocks the expression of DNA Damage Repair (DDR) proteins (MSH2, MSH6, PMS2, MLH1, Rad51, CHK1 and FOXM1) (27). Treatment of cells

with selinexor alone did not induce DNA damage in cancer cells. However, selinexor could sensitize cancer cells to DNA damaging agents by inhibiting DNA repair.

Validation of the synergism between selinexor and DNA damaging agents was done in several preclinical models including AML, colorectal cancer, non-small cell lung cancer and pancreatic cancer.

As an example, the combination of KPT-330 with cisplatin decreased the proliferation and increased the apoptosis of different human NSCLC (non-small cell lung cancer) cell lines *in vitro* (28).

Additionnally, the combination of KPT-330 with gemcitabine leads both to decreased proliferation and increased apoptosis of various human pancreatic cancer cell lines *in vitro* and to a tumor growth inhibition in vivo in nude mice (29).

Furthermore, the combination of dexamethasone and selinexor showed enhanced anti-tumor potency *in vitro* and *in vivo* (30, 31).

At the clinical level, selinexor showed single agent activity in various human clinical trials in hematological malignancies including relapsed refractory B-cell lymphomas (KCP-330-002 [NCT01607905]: Phase 1 study in patients with advanced hematological malignancies, KCP-330-009 [NCT02227251]: Phase 2 study in patients with advanced Diffuse Large B Cell Lymphoma). In addition, selinexor is currently being administered in combination with various chemotherapeutic and targeted therapies as part of investigator sponsored trials (refer to www.clinicaltrials.gov).

Although there is no direct clinical data from combination of selinexor with R-DHAOx or R-GDP in lymphoma patients, there is preliminary clinical data for selinexor being safely and effectively combined with cytarabine based regimens in 6 independent trials (NCT02212561, NCT02403310, NCT02573363, NCT0224909, NCT02299518, NCT02416908) in adult and pediatric hematological malignancies including relapsed refractory B-cell lymphomas. In addition, selinexor was also combined with Rituximab as part of the RICE regimen (Rituximab + Etoposide + Carboplatin + Ifosfomide) in adult population with relapsed refractory agressive B-cell lymphomas (NCT02471911). Although the results are preliminary, selinexor was administered safely and was shown to be fairly well tolerated. There is also growing body of clinical safety and efficacy data of selinexor being combined with various regimens in the solid tumor settings: Capecitabine, radiation and selinexor in neoadjuvant setting in locally advanced rectal cancer patients (NCT02137356) and carboplatin and selinexor, paclitaxel and selinexor, carboplatin, paclitaxel and selinexor, eribulin and selinexor, doxorubicin, cyclophosphamide and selinexor, carboplatin, pemetrexed and selinexor, topotecan and selinexor in advanced or metastatic solid tumor patients (NCT02419495).

We postulate that the addition of selinexor to salvage immunochemotherapy will improve the response rate, without decreasing the mobilization and collection of peripheral blood stem cells (PBSC), and the number of patients who can be cured with ASCT (if performed).

As for the potential additional toxicity of the association of selinexor with R-DHAOx and R-GDP, we do not expect drug interaction between selinexor and the components of these regimens, as they are metabolized by different pathways. Only cisplatin is metabolized through the same mecanism as selinexor (GSH conjugation and glucuronidation), but this is not necessarily indicative of a drug-drug interaction.

We may expect an increased frequency of thrombocytopenia with the associations, due to the specific platelet toxicity of selinexor and chemotherapies (cisplatin particularly).

To limit the potential risks (known or unknown) of the association of selinexor with R-DHAOx or R-GDP, patients will be closely monitored through hematology (complete blood count) and biochemistry testing and clinical examination (including, body weight and vital signs) before the initiation of a new cycle (day 1), with defined criteria for treatment initiation (refer to section 8.3.3.1). Hematology and biochemistry lab tests will also be performed during each cycle on day 10 and between day 14 and 21. Dose adjustment rules have been set for selinexor and chemotherapies in case of toxicity occurrence in patients, with particular care taken regarding thrombocytopenia (refer to section 8.3.3).
Escalation of the dose of selinexor to the next planned level will be decided by the dose escalation committee only after reviewing the toxicities (and particularly DLTs) which occurred in the 3 patients (evaluable for DLT) enrolled in the previous cohort.
The potential increase in the incidence of other common toxicities between selinexor and immunochemotherapies (such as gastro-intestinal troubles or infections) should be manageable through adequate prophylactic and curative measures/medications, as described in section 8.4.
Cerebellar toxicity is described with cytarabine, and suspected with selinexor. Cerebellar toxicities are considered in the protocol as events of special interest and will be suject to expedited reporting (refer to section 13.4). Glaucoma and cataracts have been described with dexamethasone, and suspected with selinexor, and visual troubles can be oserved with oxaliplatin and cytarabine. An ophtalmological examination is planned in the protocol at baseline and in case of visual trouble occurrence during treatment.

Overall, we consider that the benefit-risk balance for this study is favorable.

4.3.3 Rationale for the second dose esclation

In both groups, toxicities appeared as early as the first week of treatment, after the second administration of selinexor. According to the DLT and to all toxicities, are suspected cumulative toxicities.

The proposed new regimen should allow a better tolerance of selinexor combined with chemotherapy and the administration of selinexor 3 weeks / 3 should cover the cycle and thus avoid periods without treatment.

4.3.4 Rationale for closing the R-DHAOx treatment

Group A bis is closed to inclusion due to potential risks of veino occlusive disease with DHAOx treatment associated to ASCT procedure. This risk is independent of this trial and a large retrospective of this combination is ongoing

5 STUDY OBJECTIVES

5.1 Primary Objective

The primary objective of this study is to determine the RP2D for selinexor when administered in adult patients with relapsed or refractory B-cell malignancies receiving either R-DHAOx (rituximab + dexamethasone + oxaliplatin + cytarabine) or R-GDP (rituximab + dexamethasone + gemcitabine + cisplatin), by assessing the maximum tolerated dose (MTD)

observed during the dose escalation part of the study. Assessment of the MTD will be performed by the analysis of the dose-limiting toxicities (DLTs).

5.2 Secondary objectives

- Determine the occurrence of all grade ≥ 3 toxicities observed with the administration of selinexor in patients receiving R-DHAOx or R-GDP.
- Determine the occurrence of grade ≥ 2 renal toxicities and grade ≥ 2 neuropathy observed with the administration of selinexor in patients receiving R-DHAOx or R-GDP.
- To assess the preliminary efficacy of selinexor in patients receiving R-DHAOx or R-GDP as reflected by the overall response rate (ORR) after 3 cycles using Lugano classification criteria. Complete and partial response rates, progression free survival (PFS), response duration (DoR), time to next anti-lymphoma treatment (TTNLT) and overall survival (OS) will be also examined.

5.3 Exploratory objectives

- For patients who proceed to Autologous Stem Cell Transplantation (ASCT), determine the feasibility of hematopoietic progenitor cell mobilization after selinexor administration with RDHAOx or R-GDP (amount of peripheral CD34+ blood cells collected, rapidity and quality of engraftment). Collection and engraftment are optional and are not part of study treatment but data will be collected for exploratory purpose.
- In patients with DLBCL: response rate and PFS will be compared according to the GCB/non-GCB profile as assessed by immunohistochemistry according to Hans algorithm after central review at the LYSA-P.

6 STUDY DESIGN

See study design schema on **Appendix 19.1**.

6.1 General overview

This is an open-label, multicenter, dose escalation, phase IB study to determine the RP2D, MTD, safety and preliminary efficacy of selinexor in adult patients with relapsed/refractory B-cell malignancies receiving R-DHAOx or R-GDP. This dose escalation phase will be followed by an exploratory expansion phase in the same population with 12 patients enrolled in each group, who will receive selinexor at the RP2D.

The choice of the conventional immunotherapy regimen which was administered to each patient, R-DHAOx (Group A) or R-GDP (Group B + Group B bis), was left at the investigator’s decision before patient’s inclusion.

- ***Part 1: Dose escalation phase***

The “3+3” design was applied for dose escalation in groups A and B, and will be applied in group B bis. The escalation will be performed in group B bis as follows: R-GDP + oral Selinexor D1, D8, D15 for 3 cycles (3-week cycles)

Groups A and B were closed to inclusion after enrollment of 7 patients in each group due to new administration regimen of Selinexor. According to observed DLT and toxicities, cumulative toxicities of selinexor were suspected. Consequently, the dose escalation committee had decided to perform a new dose escalation with a new administration regimen of Selinexor in order to avoid cumulative toxicities.

Group A bis is closed to inclusion before any inclusion due to potential risks of veino-occlusive disease with DHAOx treatment associated to ASCT procedure. This risk is independent of this trial and a large retrospective study assessing this combination is ongoing.

Different dose levels for selinexor administration will be examined sequentially group B bis by the Safety Review Committe (SRC, see the composition in section 11.1): 3 doses of Selinexor per 3 weeks cycle at **40 mg** flat (Dose Level 1, DL1) or **60 mg** flat (DL2) will be taken orally by the patient on D1, D8 and D15 of each cycle.

Dose escalation will begin at DL1 and will continue until the MTD is exceeded or until the highest dose level defined in the study (DL2) is reached.

Dose escalation to the next planned dose level will be decided by the SRC based on an overall approach through cumulative safety data (from the first patient enrolled up to the first cycle treatment of the last patient enrolled at the dose level evaluated) including the number of DLTs observed during the DLT assessment period (refer to section 8.3.2 for definition of DLT and DLT assessment period). Ad hoc meeting will take place in case of any safety-related issue or if limiting toxicities appear with an accumulative onset, outside of the DLT assessment period.
The DLT assessment period begins with the first dose of selinexor and ends immediately prior to the initiation of the second cycle (for a maximum of 35 days after C1D1).

The MTD, assessed during Cycle 1 (DLT assessment period) is defined as the highest dose level of selinexor in each group at which no more than 1 out of 6 patients experiences DLT.

The RP2D of selinexor for the expansion phase will be the MTD, or the highest tolerable dose based on the analysis of adverse events and serious adverse events outside of the DLT assessment period. This RP2D will be confirmed by an Independent Data Monitoring Committee (IDMC, see section 11.2) after the review of the results of the primary endpoint analysis.

Refer to section 8.3.1 for detailed dose escalation rules and to section 8.3.2 for definition of DLTs.

- ***Part 2: Expansion phase***

The dose escalation phase will be followed by an exploratory expansion phase in group B bis, depending on the decision of the Independent Data Monitoring Committee (IDMC) after review of safety data at the end of dose escalation part.
Patients enrolled in the expansion phase will receive selinexor at the RP2D defined by the IDMC, with the conventional regimen R-GDP.

For both parts of the study, there will be 3 periods:

- Baseline period: within 28 days before initiation of the study treatment (C1D1).
- Treatment period of 3 cycles (3-week cycles) of selinexor and immunochimiotherapy.
  The occurrence of DLTs will be monitored during cycle 1 in the dose escalation phase only.
  After 3 cycles of treatment, all patients will be evaluated for response (End of Treatment EoT evaluation) in the 10 days following the end of cycle 3. Patients who achieve adequate conditions allowing for ASCT may proceed to ASCT according to institutional protocols. **This EoT evaluation must always be done before the start of any alternate anti-lymphoma therapy or before the high-dose therapy (HDT) and ASCT procedure.**

  Stem cell mobilization and collection may be proposed at the time of hematologic recovery after cycle 2 or cycle 3 according to institutional guidelines. Stem cell mobilization, collection and engraftment are optional and are not part of study treatment but data will be collected for exploratory purpose, if performed.

  Permanent treatment discontinuation is defined as stopping treatment (immunochemotherapy and selinexor) before 3 cycles are completed for any reason.
  These patients must undergo permanent treatment discontinuation (PTD) evaluations within 4 weeks after the last drug administration. If only selinexor administration is stopped due to toxicities, it will not be considered as permanent treatment discontinuation and the immunochemotherapy administration should continue.
- Post-treatment follow-up period: every 3 months from EoT/PTD visit during the first year, then every 6 months until the end of the study. During the follow-up period, disease status, adverse events according to adverse events reporting rules, survival status, and completion of high-dose chemotherapy (HDT)/ASCT or subsequent anti-lymphoma therapy will be collected.

6.2 Anticipated study timelines

Patients will be recruited over approximately 4 years (Escalation part: 3 years; Expansion part: 1 year).
It is expected that a minimum of 23 patients and a maximum of 39 patients (or 26 if expansion phase cannot be performed) will be enrolled in this study (total for the 2 parts of the study):

- Dose escalation part: 27 patients
  - Group A : 7 patients were included
  - Group B : 7 patients were included
  - Group B bis :
    - 6 patients were included at DL1
    - 7 patients were included at DL2
- Expansion phase: 12 patients in group B bis

In both parts of the study, each patient will be treated during about 3 months (complete treatment: 3 cycles of 21 days followed by the EoT evaluation performed in the 10 days following the end of cycle 3) and followed for a minimum of 6 months after the EoT/PTD and until the end of study.

The anticipated study dates (start / end) are the following:

- First Patient enrolled in the dose escalation phase: November 2016
- Last Patient enrolled in the dose escalation phase: September 2019
- Last Patient enrolled in the expansion phase: January 2021
- Last patient followed for final analysis: October 2021

The study will end 6 months after the last patient has completed the EoT/PTD.
The total duration of the study is thus estimated to be approximately 5 years (including patients' follow-up).

7 STUDY POPULATION

Patients eligible in group B bis for both parts of this study (escalation phase and expansion phase) are those patients aged between 18 and 70 years (included), with relapsed or refractory B-cell lymphoma of any type, who are eligible for receiving R-GDP.

7.1 Inclusion criteria

Patients must satisfy all of the following criteria to be enrolled in the study in group B bis:

1. Patients with any type of relapsed or refractory B-cell lymphoma
2. Eligible to receive R-GDP regarding the investigator's opinion
3. Who received prior therapy with at least one but no more than two lines therapies for B-Cell Lymphoma
4. Patient must have measurable disease defined by at least one single node or tumor lesion > 1.5 cm
5. Aged between 18 years and 70 years (included) on date of consent signature
6. Eastern Cooperative Oncology Group (ECOG) performance status of 0, 1 or 2
7. With a life expectancy of ≥ 3 months
8. Having signed a written informed consent
9. Male patients (if sexually active with a woman of childbearing potential*) must agree to use a reliable method of birth control (see **section 8.4.2.2**) during the study treatment and for at least 12 months after the last study drug administration. Male patients must agree to not donate sperm during the study treatment and for at least 12 months after the last study drug administration
10. Female patients of childbearing potential* must agree to use two reliable methods of birth control during study treatment and for 12 months after the last dose and have a negative serum human chorionic gonadotropin (hCG) pregnancy test within 3 days prior to C1D1. Reliable methods of contraception include intrauterine devices, hormonal contraceptives [contraceptive pills, implants, transdermal patches, hormonal vaginal devices or injections with prolonged release], abstinence or sterilization of the partner (see **section 8.4.2.2**).

    * A female patient or woman is considered to have childbearing potential unless she meets at least one of the following criteria:

    → Age > 50 years and naturally amenorrhoeic for > 1 year (amenorrhoea following cancer therapy does not rule out childbearing potential)

→ Previous bilateral salpingo-oophorectomy, or hysterectomy

7.2 Exclusion criteria

Presence of any of the following will exclude a patient from enrolment in group B bis:

1. Previous treatment with selinexor
2. Known central nervous system or meningeal involvement by lymphoma
3. Contraindication to any drug contained in these regimen
4. Subjects with known Human Immunodeficiency Virus (HIV) positivity
5. Subjects with known active hepatitis B infection (positive Ag HBs) or positive serology to hepatitis B (Ag HBs or antibody anti-HBc or positive DNA PCR) or active hepatitis C infection (patients with positive HCV serology are eligible only if PCR is negative for known HCV RNA)
6. Subjects with any uncontrolled active systemic infection requiring intravenous (IV) antibiotics
7. Any of the following laboratory abnormalities within 14 days prior to first administration (C1D1) of study treatment:
    a. Absolute neutrophil count (ANC) < 1,000 cells/mm3 (1.0 x 10^9/L)
    b. Spontaneous (within 7 days of any platelet transfusion) platelet count < 100,000/mm^3 (100 x 10^9/L) (75 x 10^9/L if due to lymphoma)
    c. Serum SGOT/AST or SGPT/ALT > 5.0 x upper limit of normal (ULN)
    d. Serum total bilirubin > 2x Upper Limit of Normal (ULN), or > 5x ULN if due to Gilbert syndrom or lymphoma involvement
8. Creatinine clearance < 70 mL/min (for GDP)
9. Subjects with pre-existing ≥ Grade 2 neuropathy
10. Prior history of malignancies other than lymphoma (except for basal cell or squamous cell carcinoma of the skin or *in situ* carcinoma of the cervix or breast) unless the subject has been free of the disease for ≥ 3 years
11. Any life-threatening illness, serious active disease or co-morbid medical condition, laboratory abnormality, organ system dysfunction or psychiatric illness which, in the investigator’s opinion, could compromise the patient’s safety, interfere with the absorption or metabolism of selinexor, or put the study outcomes at undue risk, or that would prevent the subject from signing the informed consent form
12. Pregnant or breastfeeding women
13. Use of any standard or experimental anti-cancer drug therapy within 28 days of the initiation (Day 1) of study drug therapy (administration of glucocorticoïds should not exceed 1mg/kg/day in the 14 days prior to C1D1)
14. Patients with a circulating lymphocytes > 50,000/ mm3(50G/L) as these patients are at an increased risk of TLS

8 TREATMENTS

8.1 Administration of conventional immunochemotherapy regimens

Eligible patients will be treated R-GDP.

Drugs composing the R-GDP (rituximab + dexamethasone + gemcitabine + cisplatin) immunochemotherapy regimens are conventional (standard) therapies and will not be supplied by the sponsor.
Chemotherapy products are to be used according to their respective summary of product characteristics.
R-GDP will be administered according to local site practice for 3 cycles (3-week cycles, 21 days). The standard administration scheme for this immunochemotherapie is provided for information in section 8.3.

8.2 Administration of Investigational Medical Product (IMP): Selinexor

Selinexor is an investigational agent and will be supplied free-of-charge by Karyopharm Therapeutics, Inc. Product management will be coordinated by LYSARC.
Selinexor is to be used according to the latest version of Investigator's Brochure and latest version of protocol

8.2.1 Description, dosage, packaging and labeling

The Investigational Medical Product (IMP) selinexor (KPT-330) is a Selective Inhibitor of Nuclear Export (SINE) compound. Selinexor specifically blocks nuclear export by binding to the nuclear export protein XPO1.
Selinexor will be supplied and administered as coated, immediate-release tablets for oral administration Selinexor tablets will be supplied in bottles or blister packs. Tablets in blister packs will be provided in a coated tablet strength of 20 mg.
Each package will be labelled in accordance with current International Conference on Harmonization (ICH), Good Clinical Practices (GCP) and all country-specific regulatory agency requirements. Labels will include the medication name, dosage, storage conditions, and batch number, and will comply with language and legal requirements of European Union.

8.2.2 Storage conditions and stability

Selinexor tablets should be stored in a locked and secured area with access restricted to the site staff pharmacist or designee(s) **at or below 30˚C**. Room temperature storage is recommended, refrigerated is acceptable. Tablets should not be stored frozen.

Selinexor tablets are currently in on-going stability studies. The expiry will be based on concurrent stability studies and extended during the course of the study as further stability data becomes available.

8.2.3 Handlings

Qualified personnel, familiar with procedures that minimize undue exposure to themselves and the environment, should undertake the preparation, handling, and safe disposal of the chemotherapeutic agent in a self-contained and protective environment.

Selinexor is to be taken with, or within 30 minutes of, solid food consumption together with at least 120 mL of fluids (water, milk, etc.).

Selinexor will be administrated before immunochemotherapy

Tablets of selinexor should not be crushed because of increased risk of dermatologic toxicity if powder comes in contact with skin.

8.2.4 Dosage and administration of selinexor

Selinexor is supplied as 20 mg tablets.

Selinexor will be taken orally by the patient for 3 cycles (3-week cycles) on D1, D8 and D15 of each cycle.

Refer to section 8.3 for detailed treatment schedule and dosage.

For doses on non-clinic days, the patient will be provided with a supply of selinexor tablets to take home by the hospital pharmacy.

Selinexor is to be taken with, or within 30 minutes of, solid food consumption together with at least 120 mL of fluids (water, milk, etc.). Selinexor tablets should be swallowed whole and should not be crushed to prevent an increased risk of dermatologic toxicity if the powder comes in contact with skin.

8.2.5 Missed or vomited doses of selinexor

8.2.5.1 Missed doses of selinexor

Missed doses of selinexor should be managed as follows:

- If a dose was missed, the schedule of that week should be altered to accommodate two doses in that week with at least 36 hours between two consecutive doses.
- If a dose must be skipped (e.g., due to recommendation of the Investigator), the next dose will be taken as per schedule. Doses should not be administered less than 36 hours apart.

All missed and delayed doses should be documented in the patient's diary and in the eCRF.

8.2.5.2 Vomited doses of selinexor

If a dose of selinexor is vomited within 1 hour of ingestion, it will be replaced. If vomiting occurs more than 1 hour after dosing, it will be considered a complete dose.

8.2.6 Overdose of selinexor

An overdose is defined as a deliberate or accidental administration of study medication to a study patient at a dose above that which is assigned to that individual patient according to the study protocol. In the event of drug overdose, the investigator and the Sponsor should be notified immediately and the patient observed closely for AEs. The patient should be treated symptomatically as appropriate, and the incident of overdose and related AEs and/or treatment documented in the patient's medical record.

8.2.7 Initial drug supply and ordering of re-supply

The site pharmacy will be provided with an initial supply of selinexor in advance, after site initiation visit and after each patient inclusion. Drug order forms for re-supplies will also be provided with all needed contact information, along with a detailed procedure providing delivery timelines. The investigator or pharmacist will inventory and acknowledge receipt of all shipments of the IMP.

8.3 Treatment schedule and duration

Eligible patients enrolled in the study will receive oral selinexor, together with a conventional immunochemotherapy regimen (Group B: R-GDP).

During both escalation and expansion phases, R-GDP will be administered for 3 cycles at 21 days interval, according to sites' local practice (their administration scheme, dose and route are provided in Table 1 for information purposes only).

Table 1: R-DHAOx/R-GDP and selinexor treatment schedule

							3-week cycle (21 days)						
							Week 1				Week 2		Week 3
			**Drug**	**Route**	**Dose**	**Dose Level**	**D 1**	**D 2**	**D 3**	**D 4**	**D 8**	**D 10**	**D 15**
OLD REGIMEN	Group A	R-DHAOx 3 cycles at 21 days interval	Rituximab	IV	375 mg/m²	-	X						
			Dexamethasone	IV or PO	mg	-	40	40	40	40			
			Oxaliplatin	IV	130 mg/m²	-	X						
			Cytarabine (Ara-C)	IV	2 g/m²	-		X X*					
		SELINEXOR	Group A on week 1 and week 2	PO	4[illegible] mg	1#	X		X		X	X	
	Group B	R-GDP 3 cycles at 21 days interval	Rituximab	IV	375 mg/m²	-							
			Dexamethasone	IV or PO	mg	-	40	40	40	40			
			Cisplatin	IV	75 mg/m²	-	X						
			Gemcitabine	IV	1000mg/m²	-	X				X		
		SELINEXOR	Group B on week 1 and week 2	PO	40 mg	1#	X		X		X	X	
	**Group B bis**	**R-GDP** 3 cycles at 21 days interval	Rituximab	IV	375 mg/m²	-	X						
			Dexamethasone	IV or PO	mg	-	40	40	40	40			
			Cisplatin	IV	75 mg/m²	-	X						
			Gemcitabine	IV	1000mg/m²	-	X				X		
		**SELINEXOR**	**SELINEXOR** **Group B Bis** On week 1, week 2 and week 3	PO	**40 mg**	**1# bis**	**X**				**X**		**X**
					**60 mg**	**2 bis**	**X**				**X**		**X**

* 3-hour infusion, administered at a 12-hour interval
# Dose escalation will begin at DL1

For the new group B bis :
Selinexor will be taken orally by the patient for 3 cycles (3-week cycles) on D1, D8 and D15 of each cycle (dosing weeks = week 1 and week 2 of each cycle).

During dose escalation phase, different dose levels for selinexor administration will be examined sequentially in each study group (see Table 2):

- ***Dose Level 1 (DL1, <u>starting DL</u>)*:** 3 doses of selinexor at **40 mg** flat per 3-week cycle will be taken by the patient on D1, , D8 and D15 of each cycle.
- ***Dose Level 2 (DL2)*:** 3 doses of selinexor at **60 mg** flat per 3-week cycle will be taken by the patient on D1, D8 and D15 of each cycle.

Table 2: Dose levels of selinexor defined for <u>new dose escalation phase</u>

Dose level	SELINEXOR Flat dose (PO)	Days of administration per 3-week cycle	Number of patients
DL1	40 mg	D1, D8, D15	3-6 / group
DL2	60 mg	D1, D8, D15	3-6 / group

During dose expansion phase, selinexor will administered according the RP2D defined by the IDMC at the end of dose escalation phase.

Each cycle comprises 21 days and begins with the day 1 of the immunochemotherapy. The cycle 1 day 1 should start at the latest one week after the date of inclusion. Each patient is planned to receive 3 cycles.

Permanent treatment discontinuation is defined as stopping treatment before 3 cycles are completed for any reason. If only selinexor administration is stopped due to toxicities, it will not be considered as permanent treatment discontinuation and the immunochemotherapy administration should continue.

8.3.1 Escalation rules for selinexor during dose escalation phase

Patients enrolled in the dose escalation phase will receive R-GDP (Group B bis) according to the investigator's decision, with increasing doses of selinexor, depending on the dose level at which they will be enrolled. Dose levels of selinexor defined for this study are detailed in Table 2.

Dose escalation will be performed Group B bis (R-GDP) using a traditional 3+3 design.

Dose escalation will begin at dose level 1 (DL1) with the enrolment of an initial cohort of 3 patients at this dose level.

Dose escalation to the next planned dose level will be decided by the SRC based on the rules described in Table 3, depending on the number of DLTs observed during the DLT assessment period (refer to section 8.3.2 for definition of DLT and DLT assessment period).

Table 3: Escalation rules for selinexor escalation phase

Number of patients with DLT at a given dose level	Dose escalation rules
0/3 patients	Escalation to the next dose level will be decided.
1/3 patients	Another 3 patients will be enrolled at the same dose level:<br>- If 0 of the 3 new patients experiences DLT (for a total of 1/6 patients with a DLT at this dose level), escalation to the next dose level will be decided.<br>- If ≥1 of the 3 new patients experiences DLT (for a total of ≥2/6 patients with a DLT at this dose level), then MTD is exceeded and the dose escalation phase of the trial will be terminated. The dose level directly below the current dose level will be considered the MTD. An additional cohort of 3 patients will be enrolled at the MTD (if only 3 patients were treated at this dose level) to reach a total of 6 patients treated at this dose level, in order

	to confirm the MTD.
≥ 2/3 patients	MTD is exceeded and the dose escalation phase of the trial will be terminated. The dose level directly below the current dose level will be considered the MTD. An additional cohort of 3 patients will be enrolled at this previous dose level (if only 3 patients were treated at that dose) to reach a total of 6 patients treated at this dose level, in order to confirm the MTD.

The maximum tolerated dose (MTD), assessed during Cycle 1 (DLT assessment period), is defined as the highest dose level of selinexor in each group at which no more than 1 out of 6 patients experiences DLT.

Selinexor escalation will continue until the highest dose level to be tested (DL2) is reached or the MTD is exceeded. If no DLT is observed during the DLT assessment period (0/3 patients or ≤1/6 patients experiences DLT) at DL2, then DL2 will be considered the MTD.

The RP2D for the expansion phase will be the MTD, or the highest tolerable dose based on the analysis of adverse events and serious adverse events outside of the DLT assessment period.

8.3.2 DLT definition and DLT assessment period (dose escalation phase)

The DLT assessment period begins with the first dose of selinexor and ends immediately prior to the initiation of the second cycle (for a maximum of 35 days after C1D1).
Only toxicities occurring during the DLT period will be used for the purposes of defining MTD. However, toxicities that occur in all cycles could be considered in the overall decisions of the SRC and IDMC.
Patients considered non-evaluable for DLT may be replaced, but the safety profile of these patients will be included in the SRC review.
Patients considered non-evaluable for DLT are patients who do not complete Cycle 1 for reasons other than toxicity. Patients with a complete cycle 1 are patients who received immunochemotherapy and all planned doses of selinexor during the cycle 1, unless the missed doses were due to study drug related AE(s). The SRC will also review any other toxicities that do not meet the below definitions and assess for DLT determination.

A DLT is defined as any of the following events (toxicity assessed using NCI CTCAE v4.03) that is assessed as related to selinexor or to the combination of selinexor with immunochemotherapy:

- Any grade ≥ 3 non hematological toxicity, irrespective of the duration, with the following exceptions:
  - Grade ≥ 3 diarrhea, anorexia/weight loss or nausea/vomiting will be considered as DLT only if lasting more than 7 days (despite optimal standard supportive care) or if such AE is leading to skipping 2 doses of selinexor
  - Grade ≥ 3 fatigue/asthenia will be considered as DLT only if lasting more than 7 days or if such AE is leading to skipping 2 doses of selinexor
  - Electrolyte abnormalities correctable with supportive therapy

- Any grade 4 hematological toxicity (except lymphocyte count decreased/lymphopenia) lasting more than 7 days or such AE is leading to skipping 2 doses of selinexor
- Any toxicity resulting in a delay of > 14 days of the initiation of the second cycle

Alopecia (of any grade) will not be considered as a DLT.

8.3.3 Treatment modifications

Treatment modifications may include dose or schedule adjustment of immunochemotherapies or dose adjustment for selinexor. It is not always easy to assess the causality of events regarding one drug among the others. Therefore, it is left at the investigator's discretion to decide if one or more drugs are causal and to take action as described in the sections that follow.

Cycle delay is preferred over reduction of dose for recovery of hematologic and non-hematologic toxicity (refer to section 8.3.3.1).

8.3.3.1 Cycle delay

After completion of each 21-day cycle, the start of a new cycle should be delayed for a maximum of 2 weeks in case of toxicity necessitating a delay.

A patient whose cycle is delayed should be assessed weekly or more for resolution or recovery of toxicity.

However, the start of a new cycle after more than a 2-week delay may occur only if patient shows a clear clinical benefit and only after formal approval of the sponsor.

The following parameters must be met on Day 1 of each cycle for the initiation of a new cycle:

- ANC ≥ 1.0 x 10^9/L
- Platelet count ≥ 75 x 10^9/L
- Non hematologic toxicities of grade ≥ 3 must have recovered to baseline (unless authorization is given by the sponsor)

For severe hematologic toxicity (Grade ≥ 3), laboratory evaluations may be repeated more frequently as necessary until the requirements for initiation of a new cycle are met. In the event of severe renal toxicity or hepatotoxicity, laboratory tests may be repeated more frequently as necessary until the toxicity resolves.

8.3.3.2 Dose modifications for selinexor

<u>During dose escalation phase,</u> dose modifications of selinexor described below are <u>not allowed during Cycle 1, except in the case of a DLT occurrence :</u> No interruption no diminution during cycle 1. In case of toxicity occurrence, temporary interruption/reduction of selinexor dose is allowed as specified in Table 4.(from cycle 2 D1)

Up to three levels of dose reduction are permitted for selinexor, depending on the starting dose (see Table 5).

During expansion phase, dose modifications of selinexor described below are allowed whatever cycle.

Table 4: Selinexor Dose Modification Guidelines from cycle 2 D1(escalade phase) and from cycle 1 D1 for expansion phase

Toxicity and Intensity	Grade	Supportive treatment guidelines	Selinexor Dose Modification
**Fatigue (common)**			
Grade 2 lasting > 7 days or ≥Grade 3	Grade 2 lasting > 7 days or ≥Grade 3	Rule out other causes. If found to be anemic, consider transfusing for Hemoglobin <8g/dL.	Interrupt selinexor dosing until resolved to Grade 1 or baseline and provided appropriate supportive care<br>For first occurrence, restart selinexor at current dose.<br>For ≥ second occurrence,**stop definitively selinexor**<br><br>Patients with significant fatigue after several doses of selinexor may have an ongoing antitumor response. Consider an unscheduled assessment of tumor response as part of the patient's evaluation.
**Anorexia or weight Loss**			
Grade 2 weight loss<br>≥Grade 3 anorexia and weight loss	Weight loss of 10% to <20% or anorexia associated with significant weight loss or malnutrition	Rule out other causes. Consider a repeat nutritional consultation and utilize nutritional supplements (e.g., Ensure®, Boost®, etc.) Consider appetite stimulants	Interrupt selinexor dosing until resolved to Grade 1 or baseline
**Nausea and vomiting**			
Grade 1 or 2<br>(If intolerable or persistent Grade 2 not responsive to supportive care, follow guidelines for Grade 3)	Grade 1 or 2 nausea<br>OR<br>Grade 1 or 2 Vomiting	Rule out other causes. Utilize additional anti-nausea meds to supplement the protocol-required 5-HT3 antagonists	Maintain Selinexor and initiate additional antinausea medications
Grade 3	Grade 3 nausea OR Grade≥ 3 vomiting	Rule out other causes. Utilize additional anti-nausea meds to supplement the protocol-required 5-HT3 antagonists	Interrupt selinexor dosing until resolved to Grade 1 or baseline<br>If not resolved before next cycle, **stop definitively Selinexor**

Toxicity and Intensity	Grade	Supportive treatment guidelines	Selinexor Dose Modification
**Hyponatremia**			
Grade 1 (sodium levels<Normal to 130mmol/L)	NA	NA	NA
Grade 3 with sodium levels<br><130-120 mmol/L without<br>Symptoms	Sodium ≤ 130 mmol/L	Institute supportive care	First occurence :<br>Interrupt Selinexor and provide appropriate supportive care<br>Monitor until sodium returns to >130 mmol/l<br>Restart Selinexor at same dose<br><br>2nd occurrence<br>**Stop definitively Selinexor and provide appropriate supportive care**
Grade 3 with sodium levels<br><130-120 mmol/L with symptoms<br>or Grade 4 (< 120 mmol/L)		Institute supportive care	**Stop definitively Selinexor and provide appropriate supportive care**
**Diarrhea**			
Grade 2		Rule out other causes (including Infectious diarhhea)<br>institute supportive care	Interrupt Selinexor dosing until resolved to Grade 1 or baseline.<br>For first occurrence, restart Selinexor at current dose.<br>For ≥ second occurrence, **stop definitively Selinexor**
Grade 3 - 4		Rule out other causes (including Infectious diarhhea)<br>institute supportive care	Interrupt Selinexor dosing until resolved to Grade 1 or baseline.<br>**If not resolved at next cycle, Stop definitively Selinexor**
**Other Non Hematologic Adverse Events**			
Grade 3 et 4		Rule out other causes	Interrupt Selinexor dosing until resolved to ≤Grade 2.<br><br>And at the discretion of the Investigator :<br>For first occurrence, restart Selinexor at current dose.<br>**For ≥ second occurrence, stop definitively Selinexor**

Toxicity and Intensity	Grade	Supportive treatment guidelines	Selinexor Dose Modification
**Thrombocytopenia**			
Grade 1 or 2	Platelet count 25 ×109/L to <75 × 109/L		Continue dosing without interruption;<br>**if at next cycle D1, platelets count didn't recovered 75 G/L, Stop Selinexor**
Grade 3 without bleeding			
Grade 4 without bleeding	Platelet count <25 × 109/L		Interrupt Selinexor until resolution at grade 1 at D1 next Cycle<br>**If not resolved, stop Selinexor**
≥ Grade 3 with bleeding	Platelet count <50 × 109/L with concurrent bleeding		Interrupt Selinexor until resolution at grade 1 at D1 next Cycle<br>**If not resolved, stop Selinexor**
**Neutropenia**			
ANC 0.5 to 1.0 × 109/L without fever			Initiate growth factor support
ANC <0.5 × 109/L OR febrile<br>neutropenia		Initiate growth factor support	Interrupt Selinexor dosing until resolved to to ≤Grade 2 at next D1<br><br>For first occurrence, restart Selinexor at current dose.<br>For ≥ second occurrence, **stop definitively Selinexor**
**Anemia**			
Hb<8,0g/dL			Administer blood transfusions and/or other treatments per clinical guidelines
Life-threatening consequences (urgent intervention indicated)		• Administer blood transfusions and/or other treatments per clinical guidelines	**Stop definitively Selinexor**

Toxicity and Intensity	Grade	Supportive treatment guidelines	Selinexor Dose Modification
**Tumor Lysis Syndrome**			
**The presence of two or more of the following abnormalities in a patient with cancer or undergoing treatment for cancer within3 days prior to and up to 7 days after initiation of treatment :**<br>• Uric acid ≥476 lmol/l or 25% increase from baseline<br>• Potassium ≥6,0 mmol/l or 25% increase from baseline<br>• Phosphate ≥1,45 mmol/l or 25% increase from baseline (Adults)<br>• Calcium ≤1,75 mmol/l or 25% decrease from baseline<br>**Clinical tumor lysis syndrome :**<br>A patient with laboratory tumor lysis syndrome and at least one of<br>• Creatinine ≥ 1,5 x ULN (age >12 years or age-adjusted)<br>• Cardiac arrhythmia<br>• Sudden death<br>• Seizure	The presence of two or more of the following abnormalities in a patient with cancer or undergoing treatment for cancer within3 days prior to and up to 7 days after initiation of treatment :<br>• Uric acid ≥476 lmol/l or 25% increase from baseline<br>• Potassium ≥6,0 mmol/l or 25% increase from baseline<br>• Phosphate ≥1,45 mmol/l or 25% increase from baseline (Adults)<br>• Calcium ≤1,75 mmol/l or 25% decrease from baseline<br>Clinical tumor lysis syndrome<br>A patient with laboratory tumor lysis syndrome and at least one of<br>• Creatinine ≥ 1,5 x ULN (age >12 years or age-adjusted)<br>• Cardiac arrhythmia<br>• Sudden death<br>• Seizure	Initiate IV hydration<br>• Management of hypourecimia :<br>Consider hypouricemic agent :<br>- allopurinol,<br>- rasburicase at 0.2mg/kg/day as a 30 min infusion if no contraindication due to G6PD deficiency for 3-7 days with careful monitoring of electrolytes<br><br>• Management of hyperphosphatemia and hypocalcemia :<br>- Dialysis if necessary<br>- Symptomatic calcemia should be treated with calcium gluconate in the standard doses for adults<br><br>• Hyperkaliemia :<br>- Cardiac monitoring<br>- If K≥7mmol/L : medical emergency and dialysis<br>- Acute cardiotoxicity treated with short infusion of calcium gluconate with cardia monitoring<br>- Nebulized or IV salbutamol<br>- Insulin or glucose	Stop Selinexor until levels return to normal<br>Reintroduce Selinexor at normal level

Table 5: Levels authorized for reduction of selinexor

Starting dose	Dose reduction 1	Dose reduction 2
40 mg	Discontinue selinexor	
60 mg	40 mg*	Discontinue selinexor

** Reduction below this dose is not permitted. Selinexor should be discontinued if not tolerated at this dose.*

8.3.3.3 Dose modifications for R-GDP

During the study conduct, R-GDP immunochemotherapy regimen could be modified during the cycle 1 even during the dose escalation part. Dose modification will be done **only for Gemcitabine** and could be set up at **Cyle 1 Day 8** based on hematological toxicities occurred, as specified in Table 6.

Table 6: Gemcitabine dose adjustment

	Absolute Granulocyte Count (x10^9/L)	Platelets (x10^9/L)	Action This cycle
Day 8	≥1.0	AND ≥75	Give 100% dose of gemcitabine
	≥0.5 and <1.0	AND ≥75	Give 100% dose of gemcitabine and initiate GCSF*<br>Or<br>Reduce gemcitabine dose 25% from this cycle 's day 1 dose
	≥0.5 and <1.0	<75 and ≥50	Reduce gemcitabine dose 25% from this cycle's day 1 dose
	<0.5	OR <50	Omit gemcitabine dose this cycle and initiate GCSF**
*If counts presumed to be low due to marrow, treat after 1-week delay (ie at 4 weeks or day 28) despite counts<br>** GCSF should be given prophylactically for all future cycles			

And in case of DLT/ Toxicities, R-GDP immunochemotherapy regimen will be modified irrespectively of the treatment cycle.

Dose modifications (25% dose reduction for each compound, respectively) are advised according to the levels of toxicities observed:

- Grade ≥ 2 neuropathy (cisplatin)
- Grade ≥ 2 creatinine elevation (cisplatin)

In case of worsening of creatinine clearance, cisplatin could be switched to carboplatin according to following guidelines: creatinine clearance <50ml/min and carboplatin AUC 4.

8.4 Selinexor Supportive Care, Contraception Requirements, and Concomitant Medications

The following concomitant medications taken by the patient from the date of inclusion and up to 30 days after the last dose of study drug (or beyond, if treatment taken for SAE/AE management) must be reported in the study eCRF:

- Administration of glucocorticoïds
- G-CSF received (number of days)
- All therapeutic blood component therapy administered (packed cells or platelet transfusion)
- Any concomitant treatment or therapy used to treat a reported AE or SAE, regardless of the time of occurrence

8.4.1 Required 5-HT3 Antagonists

In order to minimize nausea, unless contraindicated, all patients should receive 5-HT3 antagonists (e.g ondansetron 8 mg or equivalent), starting on C1D1 before the first dose of selinexor and continued 2-3 times daily thereafter, as needed. Alternative treatment may be provided if the patient does not tolerate 5-HT3 antagonists.

8.4.2 Supportive Care

Supportive measures for optimal medical care should be provided to patients during participation in this study. Based on clinical observations in 730 adult patients analyzed for safety as of 31 May 2015, the main side effects are primarily related to anorexia with poor caloric and fluid intake leading to weight loss, fatigue, and nausea. Thrombocytopenia also occurs, although it is rarely associated with bleeding.

In addition to the required prophylactic therapy with 5-HT3 antagonists (Section 8.4.1), supportive care including additional anti-nausea/anti-emetic therapy such as neurokinin (nk1) receptor antagonists (e.g aprepitant), acid suppression (proton-pump inhibitors and/or H2-blockers) and other treatments may be administered per institutional guidelines.

Additional supportive care recommendations for managing AEs are included in Table 4.

8.4.2.1 Infection

Patients can receive prophylactic antibiotics (sulfomethoxazole/trimethoprim) and antiviral therapy (valacyclovir, acyclovir or valgancyclovir) according to site local practice.

Appropriate broad-spectrum intravenous antibiotics and antifungal agents should be started immediately in patients who develop fever or other signs of systemic infection. Selinexor should be suspended in any patient with Grade 4 infection or clinical sepsis (in the absence of documented infection) until the condition is stabilized. Selinexor can then be re-started at the same dose. See also Table 4.

8.4.2.2 Contraception Requirements

Patients should not become pregnant or father a child while on this study because it is unknown whether selinexor can affect an unborn baby. Women should not breastfeed a baby while on this study. It is important that patients understand the need to use birth control while on this study. Female patients of child bearing potential must agree to use dual methods of contraception and have a negative serum pregnancy test at Screening; male patients must use an effective barrier method of contraception if sexually active with a female of child bearing potential.

The use of two forms of contraception are required, including one highly effective and one effective method of contraception, from the following lists.

- **Highly effective methods include:**
    - Hormonal contraceptives (e.g., combined oral contraceptives, patch, vaginal ring, injectables, and implants)
    - Intrauterine device or intrauterine system
    - Vasectomy and tubal ligation
- **Effective methods include:**
    - Barrier methods of contraception (e.g., male condom, female condom, cervical cap, diaphragm, contraceptive sponge)

Notes:

- No barrier method by itself achieves a highly effective standard of contraception
- The proper use of diaphragm or cervical cap includes use of spermicide and is considered one barrier method.
- The cervical cap and contraceptive sponge are less effective in parous women.
- The use of spermicide alone is not considered a suitable barrier method for contraception.

  - When used consistently and correctly, “double barrier” methods of contraception (e.g., male condom with diaphragm, male condom with cervical cap) can be used as an effective alternative to the highly effective contraception methods described above.
  - Male and female condoms should not be used together as they can tear or become damaged.

Acceptable methods of contraception also include:

- A sexual partner who is surgically sterilized or post-menopausal.
- Total (true) abstinence (when this is in line with the preferred and usual lifestyle of the patient), is an acceptable method of contraception. Periodic abstinence (e.g., calendar, ovulation, symptothermal, post-ovulation methods) and withdrawal are not acceptable methods of contraception.

For both male and female patients, effective methods of contraception must be used throughout the study and for 12 months following the last dose.

Please see Section 13.7.1 for additional safety information related to pregnancy.

8.4.3 Non-study drug Related Concomitant Medication and Treatment

Concomitant medications include any prescription or over-the-counter preparation, including vitamins, dietary supplements, over-the-counter medications, and oral herbal preparations taken during the study. Medications to treat concomitant diseases like diabetes, hypertension, etc., are allowed. Patients may continue their baseline medication(s).

8.4.3.1 Study Related Concomitant Medication

For supportive care and dose adjustment guidance for /R-DGDP immunochemotherapy regimens, please refer to guidelines in the prescribing information provided by the manufacturer (see also section 8.3.3.3). In addition, patients will receive concomitant medications to treat symptoms, AEs, and intercurrent illnesses that are medically necessary as part of standard care. Patients should also receive tumor lysis prophylaxis.

Administration of glucocorticoïds is allowed during the study but should not exceed 1 mg/kg/day in the 14 days prior to C1D1.

Patients should also receive tumor lysis prophylaxis: If TLS risk factors are identified provide prophylactic IV hydration, regular monitoring of renal function (at least sodium, potassium, chlorid, calcium, Bicarbonate, BUN, uric acid, LDH, Phosphorous, creatinine) and, if appropriate, administration of hypouricemic agents as you would to reduce the risk of TLS.

Per protocol, all patients should maintain normal hydration status and caloric intake.

8.4.3.2 Use of Blood Products

During treatment, patients may receive red blood cell (RBC) or platelet transfusions, if clinically indicated, per institutional guidelines. Patients who require repeated transfusion support should be discussed with the Sponsor.

Appropriate anti-coagulation is allowed during the study (e.g., low molecular weight heparin, direct factor Xa inhibitors, etc.). Warfarin is allowed during the study provided patients are monitored for INR twice a week during the first two cycles of therapy, then weekly to biweekly thereafter.

Patients may receive supportive care with erythropoietin, darbepoetin, granulocyte-colony stimulating factor (G-CSF), pegylated growth factors, and platelet stimulatory factors, in accordance with clinical practice or institutional guidelines prior to entry and throughout the study.

8.4.4 Restrictions for Selinexor

Medications: There are no restrictions on the use of acetaminophen or acetaminophen-containing products in combination with selinexor, EXCEPT on days of selinexor dosing, when acetaminophen use must not exceed a total daily dose of 1 gram.

Patients should not take GSH-, S-adenosylmethionine (SAM)-, or N-acetylcysteine (NAC)-containing products during their participation in this study as these products may enhance the metabolism of selinexor (refer to appendix 14 for the list of concerned products). However, they are permitted if the patient has elevated liver function tests. Patients should review all concomitant medication with the Investigator to avoid the use of these products. Patients should be carefully instructed to avoid the use of drugs or dietary supplements containing glutathione, S-adenosylmethionine, N-acetylcysteine, or high-dose vitamin C (which may affect the metabolism of glutathione) during their treatment, as they may lower selinexor concentration.

Diet: There are no dietary restrictions on this study. Patients on selinexor should maintain adequate caloric and fluid intake.

8.4.5 Prohibited Medications

Concurrent therapy with any approved or investigative anticancer therapeutic (including radiotherapy) outside of those included in this study is not allowed. Use of any immunosuppressive agents during the study must be approved by the Sponsor prior to use. Refer to the full prescribing information of drugs composing R-GDP regimen or the most current information on prohibited concurrent medications.

If a patient's clinical status requires administration of a prohibited medication, then administration of study drug should be discontinued.

The change in clinical status mandating the use of the medication in question must be reported as the reason for study drug discontinuation.

Treatment which could increase Selinexor exposition is forbidden.

8.4.6 Stem cell mobilisation and collection (not part of the study)

Stem cell mobilization and collection can be proposed by investigators, but only after the completion of at least cycle 2. Conditionning regimen will be administered according to institutionnal guidelines. G-CSF (or PEG-G-CSF) should be given from Day 6 (Day 3 for Peg-G-CSF) for 7 days or until neutrophil recovery (ANC ≥ 1000/mm^3) or according to each centre procedure for stem cell mobilization. These treatments will be collected in the eCRF for exploratory purpose.

8.5 Drug Dispensation and accountability

8.5.1 Responsibilities

In accordance with the requirements of national regulatory agencies, the investigator or the pharmacist attached to the investigator's department will sign a receipt for the IMP received and will be held accountable for proper storage, dispensation, and disposal of IMP.

All IMP supplies that will be used in the study must be maintained securely, and stored in accordance with labeling, under the responsibility of the hospital pharmacist or the investigator or other personnel allowed to store and dispense drugs, as specified by the Sponsor and in accordance with the applicable national regulatory requirements.

All drugs shall be dispensed in accordance with the investigator's prescription. It is the investigator's responsibility to ensure that an accurate record of IMP received, dispensed, returned and destroyed is maintained. The IMP traceability at site must be available as it could be asked by the sponsor at any moment during the study.

Any quality issue noticed with the receipt or use of an IMP (default in condition, appearance, pertaining documentation, labeling, expiry date, etc.) should be promptly notified to the Sponsor, who will initiate a complaint procedure.

Under no circumstances the investigator will supply study drug to a third party, allow the study drug to be used other than as directed by this protocol or destroy or dispose of drug in any other manner.

8.5.2 Accountability and compliance

Administration of the study treatment will be supervised by the investigator or sub-investigator.

The investigator or pharmacist will keep accurate records of the quantities of the IMP dispensed and used for each patient, to assess the patient treatment compliance.

Patient's compliance to selinexor dosing and administration schedule will be assessed by the investigator or its designee at each patient's visit and recorded in source documents after review of patient's diary and discussion with the patient. The investigator or its designee will account for the number of tablets dispensed against those returned by the patient. Any deviations and missed doses will be recorded in the eCRF and drug accountability logs for verification with the reasons. The investigator / designee will attempt to ensure complete compliance with the dosing schedule by providing timely instructions to the patients. In case of non-compliance, the patients will be instructed again.

The sponsor representative will periodically check the study drug accountability records held by the investigator or pharmacist to verify accountability of all IMP used. The Sponsor will verify that a final report of drug accountability at the unit dose level is maintained and archived in the investigator study file.

Selinexor should not be used for any purpose outside the scope of this protocol, nor can selinexor be transferred or licensed to any party not participating in the clinical study.

8.5.3 Destruction

All unused and undelivered IMP will be destroyed by the Investigator or the pharmacist after the Sponsor provides a written authorization. All IMP returned by the patients will be destroyed by the Investigator (or the pharmacist) after the Sponsor validates the

accountability log. All partially used disposable IMP will be immediately destroyed by the investigator (or the pharmacist). **All destroyed IMP have to be documented by the pharmacist on a Certificate of Destruction.**

In case of a potential defect in the quality of IMP, the Sponsor may initiate a recall procedure. In this case, the investigator will be responsible for promptly addressing any request made by the Sponsor, in order to recall IMP and eliminate potential hazards.

9 STUDY FLOW CHART AND SCHEDULE OF ASSESSMENTS

9.1 Study flow chart

See on **Appendix 0**.

9.2 Baseline evaluations (Day -28 to Day -1)

The patients will be required to give written informed consent to participate in this study before any non-routine baseline evaluations are conducted (see section 10.1).

The subject's eligibility has to be evaluated during the baseline period prior to the first administration of the treatment. The assessments described below are to be conducted within 28 days (unless otherwise specified) prior to the start of study therapy (C1D1). The investigator should not repeat procedures completed as standard of care (SOC) if they are within the screening window. Data from SOC procedures are part of the medical history and may be used for study purposes.

Baseline assessments include:

- Patient's characteristics and medical history: Age, gender, height, relevant medical history, complete medical history for NHL including prior lymphoma therapies
- Tumor Lysis syndrome :
  a. Per protocol, evaluate the tumor mass by radiological examination (e.g. PET/CT, PET/MRI) for patients with DLBCL

  b. Evaluate the risk of TLS based on a clinical evaluation of comorbidity (such as presence of renal impairment or cardiac insufficiency)

  c. Correct any clinically significant hyperuricaemia, hyperkalemia, hyperphosphatemia, or pre-existing hypocalcemia

  d. If TLS risk factors are identified provide prophylactic IV hydration, regular monitoring of renal function and, if appropriate, administration of hypouricemic agents as you would to reduce the risk of TLS. Per protocol, all patients should maintain normal hydration status and caloric intake.
- Ann Arbor staging at inclusion **(refer to Appendix 19.2)** and extent of the disease with IPI, aaIPI, FLIPI or MIPI score (taking histology and group into account) at inclusion **(refer to Appendix 19.3 for IPI/aaIPI, Appendix 19.4 for FLIPI and Appendix 19.5 for MIPI)**
- Documentation of HIV, HBV (Ag HBs, Ac anti-HBs and Ac anti-HBc) and HCV serologies
- Concomitant treatments at inclusion
- Complete clinical examination (including tumor assessment) and Performance Status (refer to **Appendix 19.6**) within 14 days prior to C1D1
- Presence of B symptoms within 14 days prior to C1D1

- Body weight and BSA (Body Surface Area, calculated according to Mosteller formula, refer to **Appendix 19.7**) within 14 days prior to C1D1
- Hematology lab tests within 14 days prior to C1D1: hemoglobin, platelet count, white blood cell count (WBC) with differential (neutrophils, lymphocytes, monocytes, and when appropriate, lymphoma cells)
- Serum chemistry within 14 days prior to C1D1: ASAT/SGOT, ALAT/SGPT, Total bilirubin, gamma-GT, LDH, alcaline phosphatase, ionogram (sodium, potassium, calcium), serum creatinine, creatinine clearance
- 12-lead electrocardiogram (ECG)
- Ophthalmic examination (refer to **Appendix 19.8**)
  An ophthalmic examination will be conducted on all patients by an optometrist or ophthalmologist and is to include the following:
  Prior to dilation:
    - *best corrected visual acuity*
    - *slit lamp examination*
    - *tonometry*

  Following dilation:
    - *fundoscopy*
    - *slit lamp examination to document lens clarity*

  If a cataract/lens opacity is seen during the examination, the cataract/lens opacity will be graded according to a Grade 1-4 system (modified from Optometric Clinical Practice Guideline: Care of the Adult Patient with Cataracts: available on the American Optometric Association website: www.aoa.org) (refer to ***Appendix 19.8****).*
- For females of childbearing potential, a negative serum hCG pregnancy test must be obtained within 3 days before C1D1. Test sensitivity for hCG must be ≥ 25 mIU/mL.
- Baseline PET/CT scan (PET0) performed before administration of the study treatment (refer to **Appendix 19.9**). *Patients with non FDG-avid lymphoma can be followed within this study with CT scan (with IV contrast), at the investigator's discretion.*
- Baseline CT scan with IV contrast (in absence of contraindication) performed before administration of the study treatment is **not mandatory** and could be done at the investigator's discretion
- Mandatory bone marrow biopsy
- Any other evaluations or procedures performed at baseline needed for evaluation of disease
- Blood samples for biological studies will be collected (see **Appendix 19.12**)

The investigator will be responsible for checking that the patient fulfills all the inclusion criteria and none of the exclusion criteria based on these baseline evaluations.

A tumor sample must be made available for confirmation of the diagnosis by central pathological review (refer to section 10.4).

9.3 Evaluations during treatment period

During the 3 cycles of study treatment, the following procedures will be performed on the indicated time points for all patients enrolled in the study:

	When
Clinical examination, PS (refer to **Appendix 19.6)**, body weight and BSA (see **Appendix 19.7**)	On Day 1 (D1) of each cycle, before drug administration
Hematology [Hemoglobin, platelets, WBC with differential (neutrophils, lymphocytes*, monocytes*)]<br>** required only at D1 of each cycle*	On D1 (- 1 day), D8(± 1 day), and at least once between D15 of each cycle<br>*Results must be available and reviewed by the investigator before treatment on Day 1, D8 and 15 of each cycle.*
Serum chemistry: Ionogram* (sodium, potassium, calcium,), serum creatinine, creatinine clearance<br>** required only at D1 for cycle 2 and Cycle 3*	On D1 (- 1 day), D8 (± 1 day), and at least once between D15 of each cycle<br>*Results must be available and reviewed by the investigator before treatment on Day 1, D8 and D15 of each cycle.*
In case of TLS risks : according to local site practices: - Monitor Hydration and Chlorid, Bicarbonate, BUN, uric acid, LDH, Phosphorous,	In case of TLS risks ; according to local site practices before or/and after Selinexor + immuno-chemotherapy
Ophthalmic examination (refer to **Appendix 19.8**)	Only if clinically indicated
Reporting of DLTs and AE/SAE/AESI	- DLTs: during cycle 1 in the dose escalation phase only (see section 8.3.2)<br>- AE/SAE/AESI: Continuously, from signature of informed consent until 30 days after the last drug administration (refer to sections 13.2, 13.3 and 13.4)
Reporting of concomitant treatments taken (refer to section 8.4)	Continuously, until 30 days after the last drug administration (or after, if administered to treat AE/SAE related to selinexor)<br>*Patients will be instructed to report all concomitant treatments taken at home on their diary, and to bring it at each study visit, for review by study site personnel.*
Check patient's diary for treatment compliance	On D1 of each cycle (except for cycle 1)<br>*On C1D1, patients will be given a diary where they will be instructed to complete each dose of selinexor taken during the study. Site staff will instruct patients to bring their diary at each site visit so that it can be checked by study site personnel for treatment compliance.*

Serum hCG pregnancy test (for females of childbearing potential only; Test sensitivity for hCG must be ≥ 25 mIU/mL)	If clinically indicated

The following parameters must be met on Day 1 of each cycle for the initiation of a new cycle:

- ANC ≥ 1.0 x 10^9/L
- Platelet count ≥ 75 x 10^9/L
- Non hematologic toxicities of grade ≥ 3 must have recovered to baseline (unless authorization is given by the sponsor)

In case of inadequate laboratory values, initiation of a cycle can be delayed, as described in section 8.3.3.1.

Stem cell mobilization and collection can be proposed at the time of hematologic recovery after cycle 2 or cycle 3 according to institutional guidelines (see section 8.4.6).

Permanent treatment discontinuation is defined as stopping treatment (immunochemotherapy and selinexor) before 3 cycles are completed for any reason.

If only selinexor administration is stopped due to toxicities, it will not be considered as permanent treatment discontinuation and the immunochemotherapy administration should continue.

9.4 End of treatment (EoT) / permanent treatment discontinuation (PTD) evaluations

All patients will be evaluated in the 10 days following the end of cycle 3 (EoT), or within 4 weeks after the last drug administration in case of permanent treatment discontinuation (PTD):

- Clinical examination, PS (refer to **Appendix 19.6)** and body weight
- Presence of B symptoms
- Hematology [Hemogloblin, platelets, WBC with differential (neutrophils, lymphocytes, monocytes)]
- Serum chemistry: ASAT/SGOT, ALAT/SGPT, Total bilirubin, gamma-GT, LDH, alcaline phosphatase, ionogram (sodium, potassium, calcium), serum creatinine, creatinine clearance
- Bone marrow biopsy, only to confirm an initial documentation of Complete Response on PET-CT in subjects with bone marrow infiltration by lymphoma at baseline
- PET/CT scan (refer to **Appendix 19.9**) (PET3). *Patients with non FDG-avid lymphoma for whom injected CT scan was the prefered method of evaluation at baseline should be evaluated with injected CT scan.*
- CT scan (with IV contrast) is **not mandatory** and could be done at the investigator's discretion.
- Evaluation of disease response: response criteria will be assessed using Lugano classification criteria (see **Appendix 19.10**)
- Any other evaluations or procedures performed at baseline needed for evaluation of disease response
- Check patient's diary for treatment compliance
- Ophthalmic examination (refer to **Appendix 19.8**), only if clinically indicated

- Reporting of AE/SAE/AESI (refer to sections 13.2, 13.3 and 13.4)
- Reporting of concomitant treatments taken (refer to section 8.4)
- Serum hCG pregnancy test (for females of childbearing potential only; Test sensitivity for hCG must be ≥ 25 mIU/mL)

Patients who achieve adequate conditions allowing for ASCT may proceed to ASCT according to institutional protocols.
Stem cell mobilization, collection and engraftment are not part of study treatment but data will be collected for exploratory purpose.

This EoT/PTD evaluation must always be done before the start of any alternate anti-lymphoma therapy or before the high-dose therapy (HDT) and ASCT procedure, if performed.

To ensure comparability, baseline and EoT/PTD methods for response assessment will be performed using identical techniques.

9.5 Post-treatment follow-up assessments

After the completion of EoT/PTD evaluations, the following assessments will be performed every 3 months during the first year, then every 6 months until the end of the study (EOS, defined 6 months after the last patient has completed the EoT/PTD visit):

- Clinical examination, PS (refer to **Appendix 19.6)** and body weight
- International Working Group (IWG) assessment of progression for NHL (according to Lugano classification criteria), based on CT-scans (frequency of CT-scans is left at the discretion of investigators)
- Reporting of AE/SAE/AESI related to selinexor according to reporting rules described in sections 13.2, 13.3 and 13.4
- Collection in the eCRF of subsequent anti-lymphoma therapy administered
- Survival status

Data concerning the completion of high-dose chemotherapy (HDT)/ASCT will also be collected in the eCRF for exploratory purposes, though not part of the study.

All patients enrolled in the study should be followed according to the above requirements even if they permanently discontinued study treatment before completing cycle 3, with the exception of patients who withdrew their consent and patients enrolled but who withdrew before study treatment start.

9.6 Progression/relapse

Relapse/progression will be determined as per International Working Group (IWG) assessment of progression for NHL (according to Lugano classification criteria) (see **Appendix 19.10**).

Progressive disease should be based on clinical examination, laboratory tests, CT scan or PET/CT scan.

A pathological confirmation by biopsy of the lesion should be done locally, if possible (no central review).

10 STUDY PROCEDURES

10.1 Informed consent

To participate in this study and before any baseline or screening study-specific (non-routine) evaluation, each patient must be informed and have signed a written consent. A written informed consent for genetics studies must be signed before performing genetic analyses. The genetic consent is optional. All these consents are signed after investigator gave all required information to the patient and the patient asked all his questions.

For each informed consent form, two original copies will be completed, signed and dated by both the patient and the investigator. A copy will be provided to the patient; the other copy will be maintained in the investigator's study file.

The investigator will attest on eCRF that the patient has signed and dated the informed consent and indicate if the patient has signed the genetic consent. The participation to the clinical trial will be tracked in patient medical dossier.

10.2 Registration and inclusion procedure

Upon signature of the informed consent form, the patient will be registered directly on the data capture system by the investigator through the internet network with the address below. To access the interactive registration program, the investigator needs to enter the study name (SELINDA), a username and a password.

Internet: http://study.lysarc.info

The study site will receive back the registration number for the registered patient. Each patient will be assigned a unique registration number and will keep this number for the duration of the study. Patients will be identified to the Sponsor only by their assigned number, initials, date of birth, and sex.

Registration and completion of baseline data in the eCRF must be done before any request to the Sponsor for patient's inclusion in the study.
The investigator should fax **(+33 (0) 4 72 66 38 57**) or email (**phasesprecoces@lysarc.org**) at the same time the following documents: anonymized copy of the histopathological report (see section 10.4 and **Appendix 19.11**). **LYSARC coordination site (Tel: +33 (0) 4 72 66 93 33)** will be the contact for any request.

After verification of eligibility criteria, the Sponsor will validate, or not, the inclusion of the patient directly in the eCRF. The investigator will receive back the inclusion number and dose level (selinexor dose) to be administered to the patient enrolled.
Only patients for whom inclusion has been validated by Sponsor are authorized to receive study treatment.
The cycle 1 Day 1 should start at the latest one week after the date of inclusion.

10.3 Screen failure

Patients who sign an informed consent form and do not receive study treatment are defined as screen failures. Screen failures will be replaced. Patients in screen failure may be re-screened.

10.4 Pathological diagnosis

The pathological diagnosis of B cell lymphoma will have been performed locally before the inclusion for each patient.

In the last years, histopathological central review process has become a common and prerequisite procedure for clinical trials in the field of lymphomas. A mandatory pathological review will therefore be organized for all patients included in the trial. The goal of this central review will be to confirm the diagnosis and to precise its classification according to the WHO classification 2008.

The central pathological review will be performed at the LYSA-Pathology institute (LYSA-P):

LYSA-P

SELINDA study

Hôpital Henri Mondor - RDC Haut

51, avenue de Lattre de Tassigny, 94010 Créteil, France

For each patient, the investigator will be requested to fax **+33 (0) 4 72 66 38 57 or email (phasesprecoces@lysarc.org)** at patient's inclusion a copy of the anonymized histopathological report used to include the patient in this trial where the name and address of the pathologist having diagnosed the lymphoma will be easily identified.

After patient's inclusion, the tumor paraffin embedded blocks from the formalin fixed (FFPE) sample that have been used for diagnosis (or 10 unstained slides if block is not available) will be sent to the LYSA-P according to the process described in **Appendix 19.11**. At reception, routinely stained sections will be performed and an appropriate panel of antibodies according to morphological aspects will be applied.

Each case of DLBCL will be subclassified immunohistochemically in Germinal center B (GCB) and non-GCB subtypes according to the Hans algorithm (CD10, BCL6, MUM-1) by the LYSA reviewers. For standardization, these immunohistochemistry analyses will be centralized at LYSA-P as well as other immunostains having shown prognostic value such as C-MYC, BCL-2, IgM.

The central pathological review will be performed by at least two expert hematopathologists and will be organized at LYSA-P. A consensus diagnosis will be established and communicated to the clinical coordinator and to the initial pathologist.
Tumor block will also be used to make tissue microarray (TMA).

For the need of the ancillary study, blocks will be kept temporarily to avoid a second request. Meanwhile, the block will be at the entire disposition of the initial anatomopathology laboratory under request if they need it.

10.5 PET scan upload

The local nuclear medicine physician will be asked to upload PET0 and PET3 reports on the Imagys platform: https://lysarc.imagys.com.

For each patient when applicable, the following data and images will be reviewed by a panel of PET experts:

- PET0 : PET scan performed before administration of the study treatment
- PET3 : PET scan performed after the end of cycle 3 or at permanent discontinuation of treatment

The review processing is described in the **Appendix 19.9**.

10.6 Blood sampling for biological studies

Plasma banking

Proteomic analysis appears to be a powerful tool to identify new biomarkers known to influence disease outcome and response to therapy.

A plasma sample (10 mL of blood in EDTA tubes) will be collected at individual centers under a standardized procedure (**Appendix 19.12**) at baseline or on C1D1 before any treatment administration for all patients enrolled who signed the study informed consent form. All the plasma samples will be collected at the end of the trial by LYSARC.

Tubes and labels for identification will be provided by LYSARC within the investigator file, then supply for each new patient inclusion. A traceability form “LYSARC SELINDA Study – Traceability form for proteomic analyses (storage on site)” must be completed after plasma storage, **and fax to LYSARC (+33(0)4 72 66 93 71).**

Genomic DNA

Signature of informed consent for genomic analysis is required for constitutional DNA analysis.
It is highly recommended to collect blood samples (8,5ml of blood on Paxgene DNA tube) at diagnosis for all patients participating to the study.

A specific isolation tube (PAXgene DNA tube), its label and a dedicated shipment kit will be provided by LYSARC within the investigator file, and then will be sent for each new patient inclusion of patient. The tube will be shipped by Chronopost (transport cost charged to LYSARC), at room temperature. A traceability form will have to be completed after blood sample, and joined with tube when sending to Lyon Sud Hospital. All the procedure is described in **Appendix 19.12**.

11 STUDY COMMITTEES

11.1 Safety review Committee (SRC)

During the dose escalation phase, a safety review committee (SRC) will meet after the completion of each cohort of 3 patients enrolled at a given dose level.

This SRC is composed of at least one investigator of each participating site having included at least one patient in the concerned cohort, at least one of the study coordinating investigators, the sponsor’s safety officer, the sponsor’s study statistican and the sponsor’s study project manager.

SRC meetings will be organized (by phone) for each dose level after the 3rd patient of a cohort have completed cycle 1 to decide if it is appropriate to escalate to the next dose level or to extend the cohort to 6 patients for this dose level. In this later case, the SRC will meet again after all 3 additional patients have completed cycle 1.

Additionnal meetings of the SRC may also be requested by any of the SRC members for review of additionnal data or any safety-related issue.
Decisions of the SRC on DLT determination, dose escalation or de-escalation, exploration of alternative schedules or any other recommendation on study conduct will be made based on all available data at the time of decision-making and will be documented in SRC minutes and communicated to participating sites.

11.2 Independent Data Monitoring Committee (IDMC)

An Independent Data Monitoring Committee (IDMC), including at least three independent members (2 experts in NHL, and one statistician) will be established.

The IDMC will meet after the end of the dose escalation phase to review the study safety data and results of the primary endpoint analysis. If the dose escalation phase for one of the 2 groups ends well before that of the other one group, an IDMC can be organized for each of the 2 groups.

Based on these data, the IDMC will confirm or not the RP2D defined by the SRC for selinexor at the end of the escalation phase of the study.

Patient enrolment in expansion phase of the study is conditioned upon the decision of the IDMC.

More details about IDMC operation will be provided in the IDMC Charter.

12 CRITERIA FOR PREMATURE DISCONTINUATION OF THE STUDY

12.1 Permanent treatment discontinuation (PTD)

Permanent treatment discontinuation is defined as stopping treatment before 3 cycles are completed for any reason.

If only selinexor administration is stopped due to toxicities, it will not be considered as permanent treatment discontinuation and the immunochemotherapy administration should continue.

Circumstances that lead to permanent treatment discontinuation of a patient must be reported by the investigator on patient's source documents and on the appropriate eCRF page.

Criteria for subject permanent treatment discontinuation include (but are not limited to):

- Death,
- Unacceptable toxicity, that would be, in the investigator's opinion, detrimental to the patient's well-being
- lymphoma progression/relapse,
- Pregnancy of female patient
- concomitant disease or any other condition that would, in the investigator's opinion, preclude patient from receiving study treatment,
- noncompliance (including loss of subject to follow-up),
- patient's refusal to continue treatment,
- major protocol violation, including initiation of alternate anti-neoplasic therapy.

Patients who permanently discontinue study treatment should however remain in the trial for the purpose of follow-up and data analysis (disease progression/relapse, other antineoplastic

therapy administered, symptoms and survival status) according to the post-treatment follow-up schedule described in section 9.5, with the exception of patients who withdrew their consent.

Any patient who discontinues before completing the 3 cycles of study treatment will be encouraged to return to the study center to have at least an assessment of their tumor and a safety follow-up visit within 4 weeks after the last drug administration.

The Investigator must determine the primary reason for a patient's discontinuation of study treatment and record this information on the appropriate page of the electronic case report form (eCRF). Patients who permanently discontinue study treatment are not eligible to re-initiate study treatment on this protocol at a later date.

Patients who permanently discontinue the study before completing Cycle 1 may be replaced if considered non-evaluable for DLT.

12.2 Withdrawal of Consent

Patients are free to withdraw from the study at any time without prejudice to their treatment. When a patient decides to withdraw from the study, he/she should always be contacted in order to obtain information about the reason for withdrawal and to record any adverse events. When patient agrees, he/she should return for a study visit at the time of, or soon after withdrawal, and the relevant assessments should be performed.

If the patient explicitly states his/her wish not to contribute further to the study data, the relevant LYSARC contact should be informed and the withdrawal of consent should be documented by the investigator in the patient's case report form. However, data up to the time of consent withdrawal will be included in the data reported for the study.

12.3 Patients Lost to Follow up

Every effort will be made to contact patients who fail to return for scheduled visits. A patient is considered lost to follow-up if no information has been obtained when the last patient enrolled in the study has completed the treatment phase of the study. During this time site investigator must document at least 3 attempts to contact the patient either by phone or letter.

12.4 Premature discontinuation of the study

The sponsor reserves the right to stop the trial at any time for any reason, including medical or ethical reasons affecting the continued performance of the study, or difficulties in the recruitment of patients. The investigators will be informed of this decision in writing. Study discontinuation will also be declared to Competent Authorities and Ethics Committees according to local regulation.

The same applies to any investigator wanting to discontinue his/her participation to the trial. The investigator must immediately inform the sponsor in writing of this decision.

13 SAFETY PARAMETERS

13.1 Definitions

13.1.1 Adverse Events

An **adverse event** (AE) is any untoward medical occurrence in a patient or clinical investigation patient administered a pharmaceutical product and which does not necessarily have to have a causal relationship with this treatment.
An AE can therefore be any unfavorable and unintended sign (including an abnormal laboratory finding, for example), symptom, or disease temporally associated with the use of a medicinal product, whether or not considered related to the medicinal product. This includes any occurrence that is new in onset or aggravated in severity or frequency from the baseline condition.

13.1.2 Serious Adverse Events

A **serious adverse event** (SAE) is any untoward medical occurrence that at any dose:

- Results in death
- Is life-threatening (the term "life-threatening" in the definition of "serious" refers to an event in which the patient was at risk of death at the time of the event ; it does not refer to an event which hypothetically might have caused death if it were more severe)
- Requires inpatient hospitalization (hospitalization is defined as an inpatient admission, regardless of length of stay) or prolongation of existing hospitalization
- Results in persistent or significant disability/incapacity (the term “persistent or significant disability or incapacity” means that there is a substantial disruption of person’s ability to carry out normal life functions.)
- Is a congenital anomaly/birth defect
- Is a medically significant event.

Medical and scientific judgment should be exercised in deciding whether expedited reporting is appropriated in other situations, such as important medical events that may not be immediately life-threatening or result in death or hospitalization but may jeopardize the patient or may require intervention to prevent one of the outcomes listed in the definition above.
The term “severe” is a measure of intensity, thus a severe AE is not necessarily serious. For example, “nausea of several hours duration” may be severe but may not be clinically serious.

Hospitalizations for elective surgery or other medical procedures that are not related to an AE are not considered to be SAEs.

“Hospitalization” is the official admission to a hospital. Hospitalization or prolongation of a hospitalization constitutes criteria for an AE to be serious. A hospitalization that was planned prior to the start of the study, for a preexisting condition that has not worsened during the study, does not constitute an SAE. An emergency room visit is not considered to be a hospitalization unless it results in an official admission to the hospital.

Sudden and unexplained death should be reported as an SAE. If there is any uncertainty about a finding being due solely to progression of disease, the finding should be reported as an AE or SAE, as appropriate.

13.1.3 Adverse Event Severity/Intensity

The intensity of the AE or SAE will be graded by the investigator according to the **Common Terminology Criteria for Adverse Events (CTCAE) grading system v4.03** in the toxicity categories that have recommended grading (refer to CTCAE **v4.03** available online at http://ctep.cancer.gov/protocolDevelopment/electronic_applications/ctc.htm).
AEs not listed on this grading system will be graded according to the five-point system below:

- Mild (grade 1): Discomfort noticed but no disruption of normal daily activity
- Moderate (grade 2): Discomfort sufficient to reduce or affect normal daily activity
- Severe (grade 3): Incapacitating with inability to work or perform normal daily activity
- Life-threatening (grade 4): Substantial risk of dying at time of event
- Death (grade 5)

13.2 Adverse Events reporting rules

Due to the expected toxicity of standard immunochemotherapy regimens, all AEs of grade ≥ 3 (hematological and non hematological toxicities) and all AEs of Grade ≥2 for renal toxicities and neuropathic toxicities (CTCAE – version 4.03), as well as all toxicities, regardless of the grade, resulting in a delay >2 weeks of the initiation of a cycle or in skipping 1-2 doses (or more) of selinexor or in a reduction of selinexor dose, regardless of the relationship to investigational product, occurring **from the date of informed consent signature and up to 30 days after the last drug administration will be recorded in the AE pages of the eCRF**.

All AEs of Grade ≥2 related or potentially related to selinexor <u>such as diarrhea, anorexia/weight loss or nausea/vomiting and fatigue/asthenia</u> have to be reported in order to evaluate the safety.

When associated to a SAE and regardless of the time of occurrence and the grade, the AE must be reported as “Adverse Event” in the appropriate eCRF pages.

Whenever possible, symptoms should be grouped as a single syndrome or diagnosis. The investigator should specify the date of onset, intensity, action taken regarding trial medication, corrective therapy given, outcome of all AEs and his opinion as to whether the AE can be related to the study drug.
The assessment of whether there is a reasonable possibility of a causal relationship of AE to the study drug can be one of two possibilities:

- Unrelated (no reasonable possibility)
- Related (reasonable possibility)

All events that meet one or more criteria of seriousness (see Section 13.1.2) will be reported as SAE (see Section 13.3).
<u>General AE reporting rules:</u>

- Non-serious AE will be reported through eCRF
- Any episode of any grade of toxicity, related to a SAE must be reported as “Adverse Event” in the appropriate eCRF pages regardless of the time of occurrence
- Signs, symptoms and physical findings indicative of lymphoma or progression of lymphoma are not to be reported as “Adverse Event”

- “Alopecia” toxicity (any grade) will never be reported as “Adverse event”
- AEs reported will be considered ended (recovered without sequelae) when recovered to a grade 0 or baseline
- In case of screening failure, at least AEs corresponding to SAEs will be reported in the AEs pages of eCRF.
- When a medical history resolves or decreases at a grade lower than baseline, the new grade will be the new reference grade for following AEs
- For laboratory abnormalities, the laboratory test to be taken as reference will be the one performed nearest to the Cycle 1 Day 1.

<u>Abnormal laboratory values reporting rules:</u>

If a laboratory abnormality is one component of a diagnosis or syndrome (e.g., alkaline phosphatase and bilirubin 5 × ULN associated with cholecystitis), only the diagnosis or syndrome (e.g., cholecystitis) should be recorded on the AE page of the eCRF. If the abnormality was not a part of a diagnosis or syndrome, then the laboratory abnormality should be recorded as the AE.

An abnormal laboratory value which is not a component of a diagnosis or syndrome is considered as an AE if the abnormality:

- results in discontinuation from the study treatment; or
- requires treatment, modification/ interruption of study treatment, or any other therapeutic intervention; or
- is judged to be of significant clinical importance.

The investigator has to notify in the patient medical file all the abnormal laboratory values considered as clinically significant (write next to each abnormal laboratory value assessed as clinically significant “CS” or precise it in the medical report).

Regardless of severity grade, only laboratory abnormalities that fulfill a seriousness criterion need to be documented as a serious adverse event.

13.3 Serious Adverse Events reporting rules

All events that meet one or more criteria of seriousness (see Section 13.1.2) occurring **after the informed consent signature and up to 30 days after the last drug administration,** will be reported as SAE regardless of:

- the relationship to the investigational product
- the administration of new lymphoma therapy,
- disease progression.

A SAE that occurs after this time, including during the follow-up period, will be reported **if considered related to the investigational product**.

Whenever possible, symptoms should be grouped as a single syndrome or diagnosis. The investigator should specify the date of onset, intensity, action taken regarding trial medication, corrective therapy given, outcome of all SAEs and his/her opinion as to whether the SAE can be related to the study drugs.

General SAE reporting rules:

- Any episode of any grade of toxicities, which meets one of the seriousness criteria (see Section 13.1.2) must be reported as "Serious Adverse Event" in the appropriate SAE form, and recorded as an AE in the AE pages of the eCRF
- The following events are not to be reported as SAE if requiring hospitalization of less than 8 days:
  - Haematological toxicities (anemia, thrombocytopenia, leucopenia, neutropenia), febrile neutropenia, nausea, vomiting
  - Life-threatening and fatal events should be reported as SAE regardless the duration of hospitalization
- Signs, symptoms and physical findings indicative of lymphoma or progression of lymphoma are not to be reported as "Serious Adverse Event"
- "Alopecia" toxicity (any grade) will never be reported as "Serious Adverse Event"
- Hospitalizations not to be considered as SAEs are :
    - Planned hospital admissions or surgical procedures for an illness or disease which existed before the patient was enrolled in the study or before study drug was given are not to be considered SAEs unless the condition deteriorated in an unexpected manner during the study (eg surgery was performed earlier than planned).
    - A procedure for protocol therapy administration or protocol/disease-related investigations. Hospitalization or prolonged hospitalization for a complication will be reported as an SAE
    - Routine treatment or monitoring of the studied indication (e.g. administration of blood or platelet transfusion) not associated with any deterioration in condition. Hospitalization or prolonged hospitalization for a complication remains a reportable SAE
    - Hospitalization or prolongation of hospitalization for technical, practical, or social reasons, in absence of an AE

13.3.1 Emergency outpatient treatment or observation that does not result in admission, unless fulfilling other seriousness criteria above (section 13.1.2) Obligations of the investigator

In a case of SAE the investigator must immediately (within 24 hours of first knowledge of the event by study personnel):

- **Complete SAE form with all relevant information regarding SAE**

All SAE forms must be dated and signed by the responsible Investigator or one of his/her authorized staff Members.

- **SEND the SAE pages to:**

LYSARC Pharmacovigilance department
FAX: +33 (0) 3 59 11 01 86
Email: pharmacovigilance@lysarc.org

- Attach the anonymized copy of all examinations carried out and the dates on which these examinations were performed. Care should be taken to ensure that the patient's identity is protected and the patient's identifiers in the Clinical study are

properly mentioned on any copy of source document. For laboratory results, include the laboratory normal ranges.

- Follow up of any SAE that is fatal or life-threatening should be provided within one calendar week.

For SAEs, the following must be assessed: relationship to each study drug, action taken, and outcome to date. The assessment of whether there is a reasonable possibility of a causal relationship is usually made by the investigator; it can be one of two possibilities:

- Unrelated (no reasonable possibility)
- Related (reasonable possibility)

Items to be considered when assessing the relationship of a SAE to the study drug are:

- Temporal relationship of the onset of the event to the initiation of the study drug
- The course of the event, considering especially the effect of discontinuation of study drug or reintroduction of study drug, as applicable
- Whether the event is known to be associated with the study drug or with other similar treatments
- The presence of risk factors in the study subject known to increase the occurrence of the event
- The presence of non-study drug-related factors which are known to be associated with the occurrence of the event.

13.3.2 Obligations of the Sponsor

The completed SAE forms received by the Sponsor must be e-mailed to: pvg@karyopharm.com

During the course of the study, the Sponsor will report in an expedited manner all SAEs that are both unexpected and at least reasonably related to the investigational product, to the EMA, Health Authorities, Ethic Committees in each country in accordance with international and local regulations, and to the Investigators. The causality assessment given by the investigator should not be downgraded by the sponsor. If the sponsor disagrees with the investigator's causality assessment, the opinion of both the investigator and the sponsor should be provided with the report.

The expectedness of a serious adverse reaction will be determined by the Sponsor according to the reference safety information (Investigator's Brochure) of the investigational product.

LYSARC Pharmacovigilance department will report all safety information from the trial in the Development Safety Update Reports and will notify the reports to the Health Authorities and Ethics Committees in accordance with international and local regulations.

13.4 Reporting of Adverse Events of special interest

13.4.1 Expedited reporting of cerebellar toxicities

Cerebellar toxicities are considered as AEs of special interest (AESI) by regulatory authorities for this study and require close attention from investigator if occurring.

All cases of Grade 3 or higher cerebellar toxicities occuring in patients enrolled in the study from the informed consent signature and up to 30 days after the last drug administration, or after if related to study drug, **must be immediately reported as SAE**

(even if not meeting the definition of a SAE) to the Sponsor by the investigator **within 24 hours of first knowledge of the event by study personnel**, using the SAE form provided by the Sponsor (follow reporting rules for SAE in section 13.3).

13.4.2 Reporting of Cataracts

Sponsor is closely monitoring the occurrence of cataracts during treatment with selinexor as adverse events of special interest. Ophathmic examinations are planned on regular basis to identify cataracts or worsening of existing cataracts. Any cataracts or worsening of existing cataracts has to be reported as an AE irrespectively of grade.

13.4.3 Tumor lysis Syndrome

As of May 2018, approximately 2600 patients had been treated with at least 1 dose of selinexor. Among these 2600 patients, there were 8 reports of TLS: 4 patients in Karyopharm-sponsored studies, 2 patients in Investigator-sponsored studies, and 2 patients in the expanded access program (compassionate use). Of the 8 patients, 5 had multiple myeloma reported as their underlying cancer and 3 had hematological malignancies (including AML and acute lymphoblastic leukemia). The event onset latency ranged from 3 to 8 days (median 4 days). The total selinexor dose prior to event onset ranged from 40 to 320 mg (median 160 mg). The outcome was reported as recovered in 4 patients, not recovered in 2 patients, and the outcome was not reported in 2 patients. The Investigators assessed 7 of the events as related to selinexor. Of the 8 cases summarized above, there were 3 cases with Grade 5 TEAEs reported. The cause of death in each of these cases was reported as: Respiratory failure secondary to advanced multiple myeloma (Grade 5), Sepsis (Grade 5), and Respiratory failure, chemotherapy induced cardiomyopathy and acute lymphoid leukemia (Grade 5). No fatal outcomes due to TLS have been reported in any studies with selinexor, or in the ongoing expanded access program. Although the incidence of TLS is low (~0.3%), the causal relationship between selinexor treatment and TLS cannot be completely excluded. Early recognition of signs and symptoms in patients at risk for TLS, including identification of abnormal clinical and laboratory values, is key and Investigators must ensure that patients being treated with selinexor maintain adequate caloric and fluid intake. Close monitoring and management of patients with hematological malignancies, including multiple myeloma, for potential signs and symptoms of TLS are most relevant.

See **Table 4** for supportive care and for selinexor dose modification guidance.

13.5 Suspected Unexpected Serious Adverse Reactions

Suspected, unexpected serious adverse reactions (SUSARs) will be collected and reported to the competent authorities and relevant ethics committees in accordance with Directive 2001/20/EC or as per national regulatory requirements in participating countries.
In addition, the Sponsor will communicate all cases of cerebellar toxicities, Grade 3 or higher, to the regulatory authorities, ethics committees (e.g., IRBs), and Investigators, in the format of an expedited Safety Report, within 7 days of awareness of the event.

13.6 Follow up of AEs and SAEs

Any SAE should be monitored until it is resolved or is clearly determined to be due to a patient’s stable or chronic condition or underlying condition. Any additional information known

after the event has been initially reported should be sent to LYSARC as soon as information becomes available.

All AEs as defined in section 13.2 must be documented and the outcome must be followed-up until the return to grade 0 or grade at baseline or consolidation of the patient’s condition.

Subjects who permanently discontinued study treatment due to any AE will be followed at least until the outcome is determined even if it implies that the follow-up continues after the patient has left the trial.

13.7 Procedures for Handling Special Situations

13.7.1 Pregnancy

A female patient or woman is considered to have childbearing potential unless she meets at least one of the following criteria:

→Age > 50 years and naturally amenorrhoeic for > 1 year (amenorrhoea following cancer therapy does not rule out childbearing potential)

→Previous bilateral salpingo-oophorectomy, or hysterectomy

Pregnancies and suspected pregnancies (including a positive pregnancy test regardless of age or disease state) of a female patient occurring while the patient is on study drug, or within 12 months of the patient’s last dose of study drug, are considered events to be reported immediately to LYSARC Pharmacovigilance on the appropriate Pregnancy Form:

LYSARC fax number +33 (3) 59 11 01 86

Email: Pharmacovigilance@lysarc.org

If the female patient is on study drug, the study drug is to be discontinued immediately and the patient instructed to return any unused portion of the study drug to the Investigator.

The exposure of any pregnant female (e.g. caregiver or pharmacist) to study drug is also an immediately reportable event.

The female should be referred to an obstetrician/gynecologist preferably one experienced in reproductive toxicity for further evaluation and counseling.

The Investigator will follow the female subject until completion of the pregnancy, and must notify LYSARC immediately about the outcome of the pregnancy (either normal or abnormal outcome).

If the outcome of the pregnancy is abnormal (i.e., spontaneous or therapeutic abortion) the Investigator should report the abnormal outcome as an AE. If the abnormal outcome meets any of the serious criteria, it must be reported as an SAE within 24 hours of the Investigator’s knowledge of the event using the SAE Report Form, or approved equivalent form.

All neonatal deaths that occur within 28 days of birth should be reported, regardless of causality, as SAEs. In addition, any infant death after 28 days that the Investigator(s) suspects to be related to the in utero exposure to the study drug should also be reported to LYSARC within 24 hours of the Investigator’s knowledge of the event using SAE form.

13.7.2 Male patients

If a female partner of a male patient taking study drug becomes pregnant, the male patient taking study drug should notify the Investigator, and the pregnant female partner should be advised to call her healthcare provider immediately.

If a pregnancy related event is reported in a female partner of a male patient, the investigator should determine whether the female partner is willing to release her medical information to LYSARC Pharmacovigilance and allow the pregnancy related event to be followed-up to completion.

13.7.3 Overdose

An overdose is a deliberate or accidental administration of study treatment to a study patient at a dose greater than that which is assigned to that patient per the study protocol. If an overdose occurs, the Investigator and the Sponsor should be notified immediately and the patient should be observed closely for AEs. Resulting symptoms should be treated, as appropriate, and the incident of overdose and related AEs and/or treatment documented in the patient's medical record and the eCRF. Any AE or SAE resulting from the overdose will be reported as described in Section 13.2 or Section 13.3 as appropriate.

14 GENERAL STATISTICAL CONSIDERATIONS

14.1 Study design

This is an open-label, multicenter, dose escalation, phase IB study of selinexor administered in adult patients with relapsed/refractory B-cell malignancies receiving either R-DHAOx (Group A) or R-GDP (Group B and B bis).
The dose escalation phase of the study will be conducted independently in these 3 groups (A, B and B bis), using a traditionnal 3+3 design.
This dose escalation phase will be followed by an exploratory expansion phase with 12 patients enrolled in group B bis, who will receive selinexor at the RP2D established by the IDMC at the end of the dose escalation phase.

14.2 Study Objectives

14.2.1 Primary Objective

The primary objective of the study is to determine the RP2D for selinexor when administered in adults patients with relapsed or refractory B-cell malignacies receiving R-DHAOX (rituximab + dexamethasone + oxaliplatin + cytarabine) or R-GDP (rituximab + dexamethasone + gemcitabine + cisplatin), by assessing the maximum tolerated dose (MTD) observed during the dose escalation part of the study. Assessment of the MTD will be performed by the analysis of the dose-limiting toxicities (DLTs).

14.2.2 Secondary Objectives

The secondary objectives are the following:

- Determine the occurrence of all grade ≥ 3 toxicities observed with the administration of selinexor in patients receiving R-DHAOx or R-GDP.
- Determine the occurrence of grade ≥ 2 renal toxicities and grade ≥ 2 neuropathy observed with the administration of selinexor in patients receiving R-DHAOx or R-GDP.
- Assess the preliminary efficacy of selinexor in patients receiving R-DHAOx or R-GDP as reflected by the overall response rate (ORR) after 3 cycles using Lugano classification criteria. Complete and partial response rates, progression free survival (PFS), response duration (DoR), time to next anti-lymphoma treatment (TTNLT) and overall survival (OS) will be also examined.

14.2.3 Exploratory Objectives

The exploratory objectives of the study are:

- For patients who proceed to Autologous Stem Cell Transplantation (ASCT), determine the feasibility of hematopoietic progenitor cell mobilization after selinexor administration with R-DHAOx or R-GDP (amount of peripheral CD34+ blood cells collected, rapidity and quality of engraftment). Collection and engraftment are optional and are not part of study treatment but data will be collected for exploratory purpose.
- In patients with DLBCL: response rate and PFS will be compared according to the GCB/non-GCB profile as assessed by immunohistochemistry according to Hans algorithm after central review at the LYSA-P.

14.3 Endpoints

14.3.1 *Primary endpoint*

The primary endpoint is the incidence rate of dose-limiting toxicities (DLTs) observed during the DLT assessment period (cycle 1) at each dose level examined (refer to section 8.3.2 for definition of DLTs and DLT assessment period).

14.3.2 *Secondary endpoints*

14.3.2.1 Secondary Efficacy endpoints

Secondary efficacy endpoints are:

- **Response Rates assessed at EoT/PTD evaluation according to the Lugano 2014 response criteria**

Response rates will be evaluated based on disease response assessment on PET-CT performed after completion of the 3 cycles of treatment (for patients who received all 3 cycles) or at permanent discontinuation of treatment (PTD evaluation, within 4 weeks after the last drug administration).

Assessment of response will be based on the Lugano classification criteria response assessment in Hodgkin and Non-Hodgkin lymphoma (Cheson, 2014). Response (CR, PR, SD, PD) and overall response (CR+PR) will be presented. Patient without response assessment (due to whatever reason) will be considered as non-responder.

- **Progression Free Survival (PFS)**

PFS is defined as the time from inclusion into the study to the first observation of documented disease progression or death due to any cause. If a subject has not progressed or died, PFS will be censored at the time of last visit with adequate assessment. Patients without documented event at the time of analysis will be censored at their last follow-up date.

- **Duration of response (DoR)**

Duration of response is defined as the time of attainment of CR or PR to the date of first documented disease progression, relapse or death from any cause. Patients alive and free of progression will be censored at their last visit with adequate tumor assessment indicating no disease progression.

- **Time to Next Anti-Lymphoma Treatment (TTNLT)**

TTNLT is defined as the time from the date of inclusion to the date of first documented administration of any new anti-lymphoma treatment (chemotherapy, radiotherapy, radio-immunotherapy or immunotherapy, with the exception of HDT/ASCT). Patients continuing in response or who are lost to follow-up will be censored on their last visit date. Patients who died (due to any cause) before having received a new anti-lymphoma treatment will be included in the statistical analysis with death being counted as an event.

- **Overall Survival (OS)**

Overall survival is defined as the time from the date of inclusion to the date of death from any cause. Alive patients will be censored at their last contact.

14.3.2.2 Secondary Safety endpoints

Summary of study drug administration including treatment duration, average dose, dose interruption/reduction will be displayed by treatment group.

Number, frequency and reasons for permanent treatment discontinuation, study discontinuation will be summarized by treatment group.

Adverse events, vital sign measurements, clinical laboratory measurements, and concomitant medications will be described according to treatment group.

AEs will be classified using the latest version of Medical Dictionary for Drug Regulatory Activities (MedDRA) coding system at the time of database lock. The severity of the toxicities will be graded according to the National Cancer Institute (NCI) Common Terminology Criteria for Adverse Events (CTCAE) whenever possible. Subsets of AEs to be summarized include serious, all NCI CTCAE grade severities, suspected treatment-related, and events that resulted in withdrawal of investigational product. The most severe grade of each preferred term for a patient will be utilized for summaries of adverse events by NCI CTCAE grade.

All AEs will be described by system organ class and preferred term according to treatment group (a patient having the same event more than once will be counted only once). AEs of special interest and SAEs will also be displayed in separate tables.

All AEs will be displayed in a by-patient listing and AEs of special interest also in a separate by-patient listings.

All deaths will be listed and also summarized by cause of death.

Clinical laboratory tests and their change from baseline will be summarized in terms of mean, standard deviation, median, minimum and maximum values by visit.

14.3.3 Exploratory endpoints

Exploratory endpoints are the following:

- ASCT will be mainly characterized by the number of collections, total of peripheral blood cells collected and time to engraftment.
- The impact of germinal center (GCB) versus non-germinal center (non-GCB) on the response rate and on the progression free survival will also be explored.

14.4 Analysis sets

14.4.1 DLT Set

The DLT Set includes all patients who completed at least one cycle of treatment.
A cycle will be considered as completed if R-DHAOx or R-GDP immunochemotherapies and all planned doses of selinexor were received during the cycle 1 unless the missed doses were due to study drug related AE(s), in which case they remain evaluable for DLT and therefore in the DLT set.

14.4.2 Safety Set

Safety set includes all patients having signed the informed consent and who took at least one dose of selinexor.

Patients will be analyzed on the actual treatment received ("as treated").

14.4.3 Efficacy Set

The efficacy set includes all patients of the safety set with baseline tumor assessment and at least one post baseline tumor assessment.

14.5 Statistical methods

14.5.1 Patients replacement

All patients who do not fulfill the DLT Set definition can be replaced. Patients with DLT will never be replaced.

14.5.2 General Approach

Continuous variables will be summarized in tables displaying sample size, mean, standard deviation, median and range; quartiles will also be presented when considered relevant.

Categorical variables will be described in counts and percentages (of non-missing data). Response rates will be expressed as percentages with their 95% Exact Clopper Pearson Confidence Interval limits.

Time to event analysis will be performed using Kaplan-Meier method. Survival probabilities, median survival and quartiles will be estimated with their 95% CI. Survival curves will be provided.

For exploratory purpose, identification of baseline prognostic factors on PFS/OS may be assessed by a two-sided log-rank test and estimates will be expressed as risk ratios based on the Cox regression analysis with 95% confidence intervals. To account for potential differences in the sensitivity of the different groups to the study drugs, each group (A and B) will be analyzed for efficacy and safety in aggregate as well as independently.

14.5.3 Efficacy Analysis

The efficacy analysis will be based on the Efficacy Set.

All efficacy measurements are based on investigator assessment. Due to the limited number of subjects and the nature of the study, all efficacy analyses will be considered descriptive. No statistical comparisons on efficacy will be performed.

Secondary efficacy endpoints will be analyzed when all subjects have completed 3 cycles or permanently discontinued treatment prior to Cycle 3 of the study:

- Response rate and Overall Response Rate (ORR)
- Survival endpoints (PFS, DoR, TTNLT and OS)

14.5.4 Safety Analysis

All safety analyses will be performed on Safety set on the actual treatment received ("as treated").

Assessment of the MTD will be performed by the analysis of the dose-limiting toxicities (DLTs). MTD and RP2D will be assessed independently in each group (Group A, B and B bis) according to Cycle 1 informations.

During the escalation phase, DLTs and all available safety data will be reviewed on an on-going basis by the SRC (see composition in section 11.1) and summarized in a conclusion for each dose level. The opening of next cohort at a given dose level will be based on this review by the Dose Escalation Committee. Complete safety data for the escalation will be summarized at the completion of escalation phase for review by the IDMC (see section 11.2).

14.6 Sample size

No formal power calculations were performed to pre-determine sample size. The total number of patients enrolled will depend on the outcome of the actual dose escalation process. Seven patients were included in both groups (A and B) in the escalation phase. Three patients were included in group

B bis at DL1. Up to 9 patients may be enrolled in the study if dose escalation of selinexor continues up to DL 2 in group B bis (R-GDP).

For the dose escalation, a minimum of 6 patients and a maximum of 9 patients will be enrolled in group B bis.

For the expansion phase, 12 patients will be enrolled at the RP2D in group B bis.

Therefore, a minimum of 23 patients and a maximum of 38 patients (or 26 if expansion phase cannot be performed) will be enrolled in this study.

14.7 Planned analyses

14.7.1 Primary endpoint analysis (Interim analysis)

The first clinical data cutoff for the primary endpoint analysis will occur after the last patient in the escalation phase completes Cycle 3. The aim of this analysis is to identify the maximum tolerated dose after 1 cycle of treatment (safety only for all patients) by group.

The primary endpoint analysis will be performed once when the group B bis achieved this first cutoff timepoint. Each group will be analyzed by cohort and dose level.

Efficacy and safety analysis based on the 3 cycles after the inclusion of the last patient in the escalation phase will also be performed for each group by dose level and in aggregate (A+B+Bbis).

14.7.2 Final analysis

The second data cutoff for the final analysis will occur 6 months after the last patient on study has completed the EoT/PTD evaluation.

Efficacy and safety analysis based on the 3 cycles after the inclusion of the last patient in the dose escalation/expansion cohorts will be performed.

The final analysis will be performed once when the two groups achieved the cutoff timepoint on patients having received the RP2D. Each group will be analyzed in aggregate as well as independently.

15 STUDY MONITORING

15.1 Responsibilities of investigators

The investigator(s) undertake(s) to perform the study in accordance with Good Clinical Practice and specifically either European 2001/20/CE and 2005/28/CE directives and ICH E6 and guidelines for the monitoring of clinical investigations.

The investigators ensure compliance with respect to the investigational drug schedule, visit schedule and procedures required by the study. The investigators agree to provide all information requested in the case report form in an accurate and legible manner according to instructions provided.

As may be required by the local legislation, the investigators will check that the patients are directly or indirectly affiliated to the national health insurance or coverage system if there is any.

15.2 Responsibilities of the sponsor

The sponsor (LYSARC) of this study has responsibilities to health authorities to take all reasonable steps to ensure the proper conduct of the study as regards ethics, study adherence, integrity and validity of the data recorded on the case report forms. Thus, the main duty of the sponsor project leader and of the Sponsor clinical research support team (LYSARC) is to help the investigator maintaining a high level of ethical, scientific, technical and regulatory quality in all aspects of the study.

At regular intervals during the study, the site will be contacted, through site visits, letters or telephone calls, by a representative of the monitoring team (LYSARC) to review study progress, investigator and subject adherence to study requirements and any emergent problems.

The frequency of site contact/visits, and data monitored are defined in the monitoring plan developed specifically for the study.

According to the guidelines on Good Clinical Practice, the sponsor representative will check the case report form entries against the source documents following the study monitoring plan. These personnel, bound by professional secrecy, will not disclose any personal identity or personal medical information.

15.3 Use and completion of electronic case report form (eCRF)

An electronic Case Report Form (eCRF) will be completed for each study subject. It is the investigator's responsibility to ensure the accuracy, completeness, legibility and timeliness of the data reported in the subject's eCRF available at the following website:

http://study.lysarc.info .

Source documentation supporting the eCRF data should indicate the subject's participation in the study and should document the dates and details of study procedures, adverse events and subject status.

The investigator and study site staff will receive system documentation, training and support for the use of the eCRF.

Use and completion of eCRF will be carried out according to the instructions provided in the data entry and monitoring guidelines...

The system will be secured to prevent unauthorized access to the data or the system. This will include the requirement of a user ID and password to enter or change data. These user ID and password transmitted by LYSARC to study sites staff are personal and confidential. The investigator has to maintain a list of individuals who are authorized to enter or correct data. All data entry and corrections are recorded in the audit trail (date of data entry/correction, name of person, type of action).

16 ETHICAL AND REGULATORY STANDARDS

16.1 Ethical principles

This study is in accordance with the principles laid down by the 18th World Medical Assembly (Helsinki, 1964) and subsequent amendments and will be conducted according to ICH/GCP guidelines.

16.2 Laws and regulations

This study is performed also in accordance with applicable laws and regulations of each country involved in the trial, as well as any applicable guidelines.

All data of the patients collected by the sponsor will be anonymized.

16.3 Informed consent

It is the responsibility of the investigator to obtain informed consent in compliance with national requirements from each subject prior to entering the trial or, where relevant, prior to evaluating the patient's suitability for the study.

The informed consent document used by the investigator for obtaining subject's informed consent must be reviewed and approved by a local Ethics Review Committee.

As LYSARC participates to the “Plan Cancer”, the informed consent document will be reviewed by a patient committee (*Ligue contre le cancer*).

The investigator must explain to potentially eligible patients the aims, methods, reasonable anticipated benefits and potential hazards of the trial and any discomfort it may entail. Patients will be informed that they are free not to participate in the trial and that they may withdraw consent to participate at any time. They will be told which alternative treatments are available if they refuse to take part and that such refusal will not prejudice future treatment.

The consent form will include a statement by which the patients allow the sponsor's duly authorized personnel (trial monitoring team) to have direct access to source data which supports data on the case report forms (e.g. patient's medical file, appointment books, original laboratory records, etc.).

The patient should receive a signed and dated copy of the informed consent form and patient information leaflet. The inclusion process will be documented in each patient’s medical records.

For genetic studies, a specific informed consent form will be signed and dated by patients

16.4 Ethics Review Committee and Competent Authorities submission

The Sponsor must submit this study to country Ethics Review Committee(s), and Competent Authorities. It is required to forward a copy of written signed opinions / approvals to investigators.

17 ADMINISTRATIVE PROCEDURES

17.1 Curriculum vitae

An updated signed copy of the curriculum vitae of each investigator and sub-investigator will be provided to LYSARC prior to their involvement in the study.

17.2 Confidentiality agreement

All goods, materials, information (oral or written) and unpublished documentation provided to the investigators (or any company acting on their behalf), inclusive of this study, the patient case report forms are the exclusive property of LYSARC.

They may not be given or disclosed by the investigator or by any person within his authority either in part or in totality to any unauthorized person without the prior written formal consent of LYSARC.

It is specified that the submission of this study and other necessary documentation to the Ethics Review Committee or a like body is expressly permitted, the Ethics Committee members having the same obligation of confidentiality.

The investigator shall consider as confidential and shall take all necessary measures to ensure that there is no breach of confidentiality in respect of all information accumulated, acquired or deduced in the course of the trial, other than that information to be disclosed by law.

17.3 Record retention in investigating site(s)

The investigator must maintain all study records, patient files and other source data for the maximum period of time permitted by the hospital, institution or private practice. The investigators will maintain a personal patient identification list (patient numbers with the corresponding patient names) to enable records to be identified.

However national regulations should be taken into account, the longest time having to be considered.

For trials performed in the European Community, the investigator is required to arrange for the retention of the patient identification codes for at least 20 years after the completion or discontinuation of the trial.

Any site will notify the sponsor before destroying any data or records.

17.4 Ownership of data and use of the study results

The sponsor has the ownership of all data and results collected during this study. In consequence, the sponsor or any third Party either appointed by the Sponsor or having concluded a specific agreement with the Sponsor, reserves the right to use the data of the present study, either in the form of case report forms (or copies of these), or in the form of a report, with or without comments and with or without analysis, in order to submit them to the health authorities of any country.

The Investigator is committed to give his support to any requests for a patent or any property title based on, or illustrated with the results of the present Study for any country.

17.5 Publication

The results of the trial will be published after complete data collection and evaluation. Partial or preliminary results can be published beforehand. Publication is to be initiated by the two coordinating investigators in charge of the study with approval of partner if applicable.

Any publication in the form of a lecture, poster or article must be basically approved by the Scientific Committee of LYSA.

The authors will be proposed (according to the updated LYSA publication rules) by the coordinating investigators in charge of the study, and finally endorsed by the Steering Committee of LYSA.

All study data and publications are the property of LYSA/LYSARC.

17.6 Insurance compensation

The sponsor certifies having taken out appropriate liability insurance policy which covers the Sponsor, the investigator and his co-workers and which is in accordance with the local laws and requirements. Specific statements will be contained in appendix where needed.

A certificate of insurance will be provided to the investigator in countries in which this document is required.

The Investigator(s) will remain responsible towards the Sponsor of any fault or misconduct regarding the performance of the Study.

17.7 Company audits and inspections by regulatory agencies

For the purpose of ensuring compliance with good clinical practice and regulatory agency guidelines it may be necessary to conduct a site audit or an inspection.

By signing this study, the investigator agrees to allow LYSARC and its representative, and drug regulatory agencies to have direct access to his study records for review. These personnel, bound by professional secrecy, will not disclose any personal identity or personal medical information.

These audits involve review of source documents supporting the adequacy and accuracy of data gathered in CRF, review of documentation required to be maintained, and checks on drug accountability.

LYSARC will in all cases help the investigator prepare for an inspection by any regulatory agency.

17.8 Clinical study report

The sponsor will declare the trial end to Competent Authorities and Ethics Committees according to local regulations.

A summary of the study results will be prepared under the responsibility of the sponsor, within one year after the end of the study and will be forwarded to Competent Authorities and Ethics Committees and posted on Authorities' website if required by local regulations.

A suitable study report will be also prepared under the responsibility of the sponsor, within one year after the end of the study if required by local regulations.

17.9 Protocol amendments

It is specified that the appendices attached to this study and referred to in the main text of this study, form an integral part of the study.

No changes or amendments to this study may be made by the investigator or by the sponsor after the study has been agreed to and signed by both parties unless such change(s) or amendment(s) have been fully discussed and agreed upon by the coordinating investigator, LYSARC and study partner.

Approval / opinion of amendments by Ethics Review Committee(s) and Competent Authorities are required prior to their implementation, unless there are overriding safety reasons.

If the change or deviation increases risk to the study population, or adversely affects the validity of the clinical investigation or the subject's rights, full approval / advice must be obtained prior to implementation. For changes that do not involve increased risk or affect the validity of the investigation or the subject's rights, approval / advice may be obtained by expedited review, where applicable.

Any change agreed upon will be recorded in writing, the written amendment will be signed by the investigator and by the sponsor and the signed amendment will be appended in the Investigator Study File.

In some instances, an amendment may require a change to a consent form. The investigator must receive approval / advice of the revised consent form prior to implementation of the change. In addition, changes to the case report forms, if required, will be incorporated in the amendment.

Modifications done between different versions of the protocol are listed below:

Version	Modifications
V1.0	Initial version
V1.1	Following requests from CPP and ANSM :<br>- Duration of contraception extended 12 months (instead of 6 months) after last dose of study treatment, to follow recommendations for rituximab<br>- Precisions added concerning concomitant treatments to avoid and addition of a representative list in appendix 14<br>- Detailed rationale added in section 4.3.2 regarding the association of selinexor with R-DHAOx/R-GDP<br>- Precision regarding selinexor intake added in the synopsis
V2.0	Amendment 1<br>- The protocol was amended consequently to the update of Investigator’s Brochure for Selinexor (IB V6.0; date 14 November 2016). Additional safety data are leading us to modify the dose modification rules for Selinexor from cycle 2 during the dose escalation phase in order to limit the potential toxicities of combination of Selinexor and immunochemotherapy regimens (R-DHAOX or R-GDP)<br>- The dose modification rules of R-GDP immunochimiotherapyare also modified in regards with the potential toxicities of the combination of Selinexor and

	Gemcitabine especially the hematological toxicities such as thrombocytopenia and neutropenia. Gemcitabine dose adaptation could be done at cycle 1 day 8 in case of hematological toxicities<br>- R-GDP scheme administration was corrected in order to meet the standard of care<br>- The DLT definition was modified In order to enable the implementation of the new recommendations for gemcitabine dose adjustement in R-GDP cyle 1<br>- Catarct is an AESI<br>- Switch between cisplatin and carboplatine is allowed with guidelines
V2.1	Non substantial modification<br>-Annexe 15 : R-GDP guigelines were corrected
V3.0	Amendment 4<br>- New dose escalation :<br>- new administration regimen of Selinexor : once weekly at D1, D8 and D15<br>- only 2 level doses : 40 mg and 60mg<br>- Selinexor dose guidelines are modified for thrombocytopenia
V4.0	Amendment 5<br>- Tumor Lysis Syndrom
V4.1	Following request from ANSM : add in table 8 TLS symptoms and supportive treatment guidelines
V5.0	Amendment 6 : R-DHAOX arm closure
V6.0	Amendment : Selinexor dose adjustment modified (IB V9.0)

18 REFERENCES

1. Swerdlow SH, Campo E, Harris NL et al. WHO Classification of Tumors of Haematopoietic and Lymphoid Tissues; 2008.
2. Philip T, Guglielmi C, Hagenbeek A et al. Autologous bone marrow transplantation as compared with salvage chemotherapy in relapses of chemotherapy-sensitive non Hodgkin's lymphoma. N Engl J Med 1995; 333: 1540–1545.
3. Hasberg H et Gisselbrecht C ,Randomised phase III study of R-ICE versus R-DHAP in relapsed patients with CD20 diffuse large B-cell lymphoma (DLBCL) followed by high-dose therapy and a second randomisation to maintenance treatment with rituximab or not: an update of the CORAL study.Ann Oncol. 2006 May; 17 Suppl 4:iv31-2.
4. Thieblemont C et al, The germinal center/activated B-cell subclassification has a prognostic impact for response to salvage therapy in relapsed/refractory diffuse large B-cell lymphoma: a bio-CORAL study.J Clin Oncol. 2011 Nov 1;29 (31):4079-87.
5. Lignon J, Sibon D, Madelaine I, Brice P, Franchi P, Briere J, Mounier N, Gisselbrecht C, Faure P, Thieblemont C. Rituximab, dexamethasone, cytarabine, and oxaliplatin (R-DHAX) is an effective and safe salvage regimen in relapsed/refractory B-cell non-Hodgkin lymphoma. Clin Lymphoma Myeloma Leuk. 2010 Aug;10(4):262-9
6. Crump et al, Randomized comparison of gemcitabine, dexamethasone, and cisplatin versus dexamethasone, cytarabine, and cisplatin chemotherapy before autologous stem-cell transplantation for relapsed and refractory aggressive lymphomas: NCIC-CTG LY.12.J Clin Oncol. 2014 Nov 1;32(31):3490-6.
7. Yang J, Bill MA, Young GS, La Perle K, Landesman Y, Shacham S, et al. Novel small molecule XPO1/CRM1 inhibitors induce nuclear accumulation of TP53, phosphorylated MAPK and apoptosis in human melanoma cells. PloS One. 2014;9(7):e102983.
8. Huang TT, Kudo N, Yoshida M, Miyamoto S. A nuclear export signal in the N-terminal regulatory domain of IkappaBalpha controls cytoplasmic localization of inactive NF-kappaB/IkappaBalpha complexes. Proc Natl Acad Sci U S A. 2000 Feb 1;97(3):1014–9.

9. Zhang M, Hu B, Li T, Peng Y, Guan J, Lai S, et al. A CRM1-dependent nuclear export signal controls nucleocytoplasmic translocation of HSCARG, which regulates NF-κB activity. Traffic Cph Den. 2012 Jun;13(6):790–9.
10. Zerfaoui M, Errami Y, Naura AS, Suzuki Y, Kim H, Ju J, et al. Poly(ADP-ribose) polymerase-1 is a determining factor in Crm1-mediated nuclear export and retention of p65 NF-kappa B upon TLR4 stimulation. J Immunol Baltim Md 1950. 2010 Aug 1;185(3):1894–902.
11. Muller PAJ, van de Sluis B, Groot AJ, Verbeek D, Vonk WIM, Maine GN, et al. Nuclear-cytosolic transport of COMMD1 regulates NF-kappaB and HIF-1 activity. Traffic Cph Den. 2009 May;10(5):514–27.
12. Mao L, Yang Y. Targeting the nuclear transport machinery by rational drug design. Curr Pharm Des. 2013;19(12):2318–25.
13. Lee S-H, Hannink M. Characterization of the nuclear import and export functions of Ikappa B(epsilon). J Biol Chem. 2002 Jun 28;277(26):23358–66.
14. Beck IME, Vanden Berghe W, Vermeulen L, Bougarne N, Vander Cruyssen B, Haegeman G, et al. Altered subcellular distribution of MSK1 induced by glucocorticoids contributes to NF-kappaB inhibition. EMBO J. 2008 Jun 18;27(12):1682–93.
15. Neggers JE, Vercruysse T, Jacquemyn M, Vanstreels E, Baloglu E, Shacham S, et al. Identifying Drug-Target Selectivity of Small-Molecule CRM1/XPO1 Inhibitors by CRISPR/Cas9 Genome Editing. Chem Biol. 2015 Jan 22;22(1):107–16.
16. Gravina G, Senapedis W, McCauley D, Baloglu E, Shacham S, Festuccia C. Nucleo-cytoplasmic transport as a therapeutic target of cancer. J Hematol OncolJ Hematol Oncol. 2014 Dec 5;7(1):85.
17. Parikh K, Cang S, Sekhri A, Liu D. Selective inhibitors of nuclear export (SINE)--a novel class of anti-cancer agents. J Hematol OncolJ Hematol Oncol. 2014;7:78.
18. Zhong Y, El-Gamal D, Dubovsky JA, Beckwith KA, Harrington BK, Williams KE, et al. Selinexor suppresses downstream effectors of B-cell activation, proliferation and migration in chronic lymphocytic leukemia cells. Leukemia. 2014 May;28(5):1158–63.
19. Tai YT, Landesman Y, Acharya C, Calle Y, Zhong MY, Cea M, Tannenbaum D, Cagnetta A, Reagan M, Munshi AA, Senapedis W, Saint-Martin JR, Kashyap T, Shacham S, Kauffman M, Gu Y, Wu L, Ghobrial I, Zhan F, Kung AL, Schey SA, Richardson P, Munshi NC, Anderson KC. CRM1 inhibition induces tumor cell cytotoxicity and impairs osteoclastogenesis in multiple myeloma: molecular mechanisms and therapeutic implications. Leukemia. 2014 Jan;28(1):155-65
20. Kuruvilla, John, Byrd, John, Flynn, Joseph, Garzon, Ramiro, Shacham, Sharon. The Oral Selective Inhibitor of Nuclear Export (SINE) Selinexor (KPT-330) Demonstrates Broad and Durable Clinical Activity in Relapsed / Refractory Non Hodgkin's Lymphoma (NHL). ASH Annu Meet Abstr. 2014;
21. Julia Etchin, Thi Le, Bonnie, Look AT (last). Selective Inhibitor of Nuclear Export (SINE), Selinexor (KPT-330), Shows Remarkable Activity Against AML Leukemia-Initiating Cells S. ASH Annual Meeting Abstracts [Internet]. 2014; Available from: https://ash.confex.com/ash/2014/webprogram/Paper74818.html
22. Chen, Christine I, Reece, Donna E. Selinexor Demonstrates Marked Synergy with Dexamethasone (Sel-Dex) in Preclinical Models and in Patients with Heavily Pretreated Refractory Multiple Myeloma (MM). ASH Annu Meet Abstr. 2014;
23. Das A, Wei G, Parikh K, Liu D Selective inhibitors of nuclear export (SINE) in hematological malignancies. Exp Hematol Oncol. 2015 Mar 1;4:7.
24. Gutierrez M, Goy A, Byrd JC, Flynn JM, Sorensen M, Brown P, et al. A phase 1 dose-escalation study of the oral selective inhibitor of nuclear export (SINE) KPT-330 (selinexor) in patients (pts) with heavily pretreated non-Hodgkin lymphoma (NHL) ASCO Meeting Abstracts. 2014;32(15):8518.
25. Chen C, Garzon R, Gutierrez M, Jacoby MA, Brown P, et al. Safety, Efficacy, and Determination of the Recommended Phase 2 Dose for the Oral Selective Inhibitor of Nuclear Export (SINE) Selinexor (KPT-330). ASH Annual Meeting Abstract 2015, available at https://ash.confex.com/ash/2015/webprogram/Paper85694.html
26. Gisselbrecht C, Glass B, Mounier N, Singh Gill D, Linch DC, Trneny M, Bosly A, Ketterer N, Shpilberg O, Hagberg H, Ma D, Brière J, Moskowitz CH, Schmitz N. Salvage regimens with autologous transplantation for relapsed large B-cell lymphoma in the rituximab era. J Clin Oncol. 2010 Sep 20;28(27):4184-90
27. Selective Inhibitors of Nuclear Export (SINE) Block the Expression of DNA Damage Repair Proteins and Sensitize Cancer Cells to DNA Damage (Inducing) Therapeutic Agents., Kashyap et al., Poster presented at EORTC conference 2014

28. Sun H, Hattori N, Chien W, Sun Q, Sudo M, E-Ling GL, Ding L, Lim SL, Shacham S, Kauffman M, Nakamaki T, Koeffler HP. KPT-330 has antitumour activity against non-small cell lung cancer. Br J Cancer. 2014 Jul 15;111(2):281-91)
29. Kazim S, Malafa MP, Coppola D, Husain K, Zibadi S, Kashyap T, Crochiere M, Landesman Y, Rashal T, Sullivan DM, Mahipal A. Selective Nuclear Export Inhibitor KPT-330 Enhances the Antitumor Activity of Gemcitabine in Human Pancreatic Cancer. Mol Cancer Ther. 2015 Jul;14(7):1570-81
30. Landesman Y, Kashyap T, Klebanov B, Elloul S, Crochiere M, Friedlander S, Senapedis W, Carlson R, Kauffman M, Shacham S. Selective inhibitor of nuclear export (SINE) compounds show synergistic anti-tumor activity in combination with dexamethasone in multiple myeloma. AACR 106th Annual Meeting 2015 (asbtract 2074); April 18-22, 2015
31. Kashyap T, Klebanov B, Lee MS, Landesman Y. Selinexor, a Selective Inhibitor of Nuclear Export (SINE) Compound, Shows Synergistic Anti-Tumor Activity in Combination with Dexamethasone Characterized By Specific Pattern of Gene Expression in Multiple Myeloma (MM). Blood Dec 2015, 126 (23) 3683

19 APPENDICES

19.1 Appendix 1: Study design

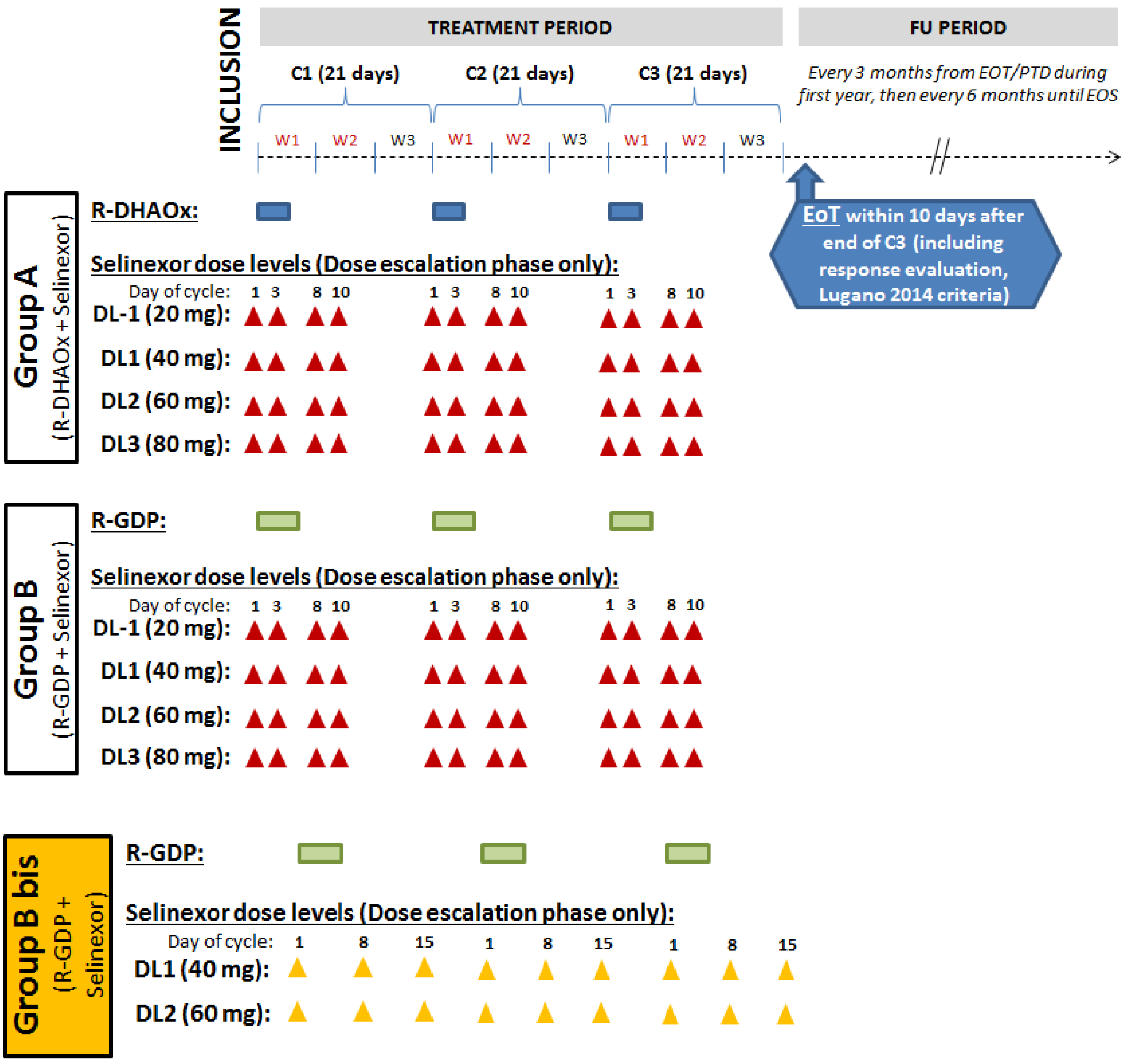

R-DHAOx (refer to Table 1) R-GDP (refer to Table 1)

▲ Selinexor dosing day (PO; twice weekly, dosing weeks= week 1 and week 2 of each cycle; refer to Table 1)

⬟ Selinexor dosing day (PO; once weekly, docing weeks = week 1, week 2 and week 3 of each cycle; refer to Table 1)

EoT: End of Treatment FU: Follow-up PTD: Permanent Treatment Discontinuation EOS: End Of Study

Appendix 2: Schedule of Evaluations (study flow-chart)

	**Baseline Period** within 28 days prior to C1D1	**Treatment Period** (1 cycle = 21 days)			**EoT/PTD evaluation** (EoT: in the 10 days following end of cycle 3; PTD: within 4 weeks after the last drug administration and before the start of any alternate anti-lymphoma therapy or before the HDT/ASCT procedure)	**Post-treatment follow-up** (Every 3 months during the first year, then every 6 months until the end of study)
		**Cycle 1**	**Cycle 2**	**Cycle 3**		
Signed written informed consent form	X					
Patient's characteristics/Medical History [a]	X					
Check inclusion/exclusion criteria	X					
Documentation of HIV, HCV, HBV serologies	X					
Inclusion (CSOnline)	X					
Presence of B symptoms	X(1)				X	
Clinical examination, PS, weight	X(1)	D1 [c]	D1 [c]	D1 [c]	X	X
BSA [b]	X(1)	D1 [c]	D1 [c]	D1 [c]		
12-lead ECG	X					
Ophthalmic examination [d]	X	If clinically indicated				
Reporting of concomitant treatments (see section 8.4)	At inclusion	Continuously				X [e]
Reporting of DLT/AE/SAE /AESI (see sections 8.3.2 and 13)		Continuously				X [f]
**Hematology** *[hemoglobin, platelet count, white blood cell count with differential (neutrophils, lymphocytes, monocytes)]*	X(1)	D1 [c] + D8+ D15	D1 [c] + D8 + D15	D1 [c] + D8 + D15	X	
**Serum chemistries – Full panel** *[ASAT/SGOT, ALAT/SGPT, Total bilirubin, gamma-GT, alcaline phosphatase, ionogram (sodium, potassium, calcium), serum creatinine, creatinine clearance]*	X(1)				X	
**Serum chemistries – Limited panel** *[Ionogram (sodium, potassium, calcium, chloride, bicarbonate, BUN, uric acid, phosphorous), serum creatinine, creatinine clearance]*		D1 [c] + D8 + D15	D1 [c] + D8 + D15	D1 [c] + D8 + D15		

LDH	X(1)	X	X	X	X	
Serum pregnancy testing (β-hCG)	X(2)	If clinically indicated			X	
Check diary cards for treatment compliance		X	X	X	X	
PET/CT scan (see Appendix 19.9)	X				X	
CT-scan (not mandatory, at the investigator's discretion)	X				X	X
Bone Marrow biopsy [g]	X				X	
Evaluation of the disease status [h]					X	X
Blood sampling for biological studies (refer to section 10.6 and Appendix 19.12)	X [i]					

(1) Within 14 days prior to C1D1
(2) For females of childbearing potential only: negative serum hCG pregnancy test must be obtained within 3 days before C1D1. Test sensitivity for hCG must be ≥ 25 mIU/mL.
(a) Age, gender, height, relevant medical history, complete medical history for NHL including prior lymphoma therapies, Ann Arbor staging and extent of disease (IPI, aaIPI, FLIPI or MIPI scores) at inclusion
(b) Body Surface Area (BSA) will be calculated by Mosteller method (refer to Appendix 19.7)
(c) Before drug administration on Day 1 of each cycle
(d) Ophthalmic examination (refer to Appendix 19.8) at baseline, then if clinically indicated
(e) If administered to treat a reported AE/SAE related to selinexor
(f) If related to selinexor
(g) Mandatory bone marrow biopsy at baseline; bone marrow biopsy mandatory at EoT/PTD only to confirm an initial complete response on PET scan in patients with bone marrow involvment at baseline
(h) Evaluation of disease response according to Lugano 2014 classification at EoT/PTD (refer to appendix 19.10); progression will be assessed as per IWG assessment of progression for NHL (according to Lugano classification criteria)
(i) Blood samples for biological studies will be collected after signature of informed consent form at baseline or on C1D1 before any treatment administration

C1, C2…: Cycle 1, Cycle 2… EoT: End Of Treatment PTD: Permanent Treatment Discontinuation

19.2 Appendix 3: Ann Arbor staging

Stage I:

- I: Involvement of a single lymph node region
- IE: Localized involvement of a single extralymphatic organ or site.

Stage II:

- II: Involvement of 2 or more lymph node regions on the same side of the diaphragm
- IIE: Localized involvement of a single associated extralymphatic organ or site and its regional lymph nodes with or without other lymph node regions on the same side of the diaphragm

Stage III:

- III: Involvement of lymph node regions on both sides of the diaphragm
- IIIE: Involvement of lymph node regions on both sides of the diaphragm accompanied by localized involvement of an extralymphatic organ or site
- IIIS: Involvement of lymph node regions on both sides of the diaphragm accompanied by involvement of the spleen
- IIIS+E: Both IIIS+IIIE

Stage IV:

- IV: Disseminated (multifocal) involvement of 1 or more extralymphatic sites with or without associated lymph node involvement or isolated extralymphatic organ involvement with distant (non regional) nodal involvement
- IVE: Extranodal lymphoid malignancies arise in tissues separate from, but near, the major lymphatic aggregates.

Source: *American Joint Committee on Cancer. Non Hodgkin's lymphoma. In: AJCC Staging Manual. 5th ed. Philadelphia, PA: Lippincott-Raven;1997:289-294.*

19.3 Appendix 4: International Prognostic Index (IPI) and age-adjusted International Prognostic Index (aa-IPI)

Source: The international Non Hodgkin Lymphoma prognostic factor project. A predictive model for aggressive non Hodgkin lymphoma. New England Journal of Medicine 1993;329:987-994.

<u>For IPI, score 1 point for each of the following risk factors:</u>

Age	> 60
Lactate deshydrogenase (LDH) level	> normal
Ann Arbor stage	III-IV
Performance status (PS)	2-4
Extra-nodal involvement	more than 1 site

RISK GROUPS	Number of Factors
Low	0-1
Low intermediate	2
High intermediate	3
High	4-5

<u>For age-adjusted IPI, score 1 point for each of the following risk factors:</u>

Lactate deshydrogenase (LDH) level	> normal
Ann Arbor stage	III-IV
Performance status (PS)	2-4

19.4 Appendix 5: Follicular Lymphoma International Prognostic Index (FLIPI)

Source: Solal-Celigny et al. Follicular lymphoma international prognostic index. Blood 2004;104(5):1258-65.

<u>Score 1 point for each of the following risk factors:</u>

Hemoglobin	< 12 g/dL
Number of nodal areas	>4
(The spleen is considered as an extranodal site and not a nodal area)	
Age, years	> 60
LDH level	> normal
Ann Arbor Stage	III/IV

<u>RISK GROUPS</u>	<u>Number of Factors</u>
Low	0-1
Intermediate	2
High	3-5

19.5 Appendix 6: MCL International Prognostic Index (MIPI)

Source: Hoster E et al. A new prognostic index (MIPI) for patients with advanced-stage mantle cell lymphoma. Blood 2008; 111:558-565. Erratum in: Blood 2008;111(12):5761.

MIPI Score = 0.03535 X age (years)

+ 0.6978 (if ECOG PS > 1, otherwise 0)

+ 1.367 X $\log_{10}$(LDH/ULN)

+ 0.9393 X $\log_{10}$(WBC count per 10^{-6} L)

ECOG: ECOG performance status (see Appendix E), LDH: lactate dehydrogenase, $\log_{10}$: logarithm with respect to base 10, ULN: upper limit of the normal range, LDH/ULN: LDH divided by ULN, WBC: white blood cell.

Risk groups are defined by:

**MIPI risk group**	**MIPI score**
Low risk	< 5.7
Intermediate risk	≥ 5.7 and < 6.2
High risk	≥ 6.2

19.6 Appendix 7: Performance Status Criteria

The following table presents the Eastern Cooperative Oncology Group (ECOG) performance status scale:

ECOG Performance Status Scale	
**Grade**	**Description**
0	Normal activity. Fully active, able to carry on all pre-disease performance without restriction.
1	Symptoms, but ambulatory. Restricted in physically strenuous activity, but ambulatory and able to carry out work of a light or sedentary nature (e.g., light housework, office work).
2	In bed < 50% of the time. Ambulatory and capable of all self-care, but unable to carry out any work activities. Up and about more than 50% of waking hours.
3	In bed > 50% of the time. Capable of only limited self-care, confined to bed or chair more than 50% of waking hours.
4	100% bedridden. Completely disabled. Cannot carry on any self-care. Totally confined to bed or chair.
5	Dead.

Source: *Oken MM, Creech RH, Tormey DC, Horton J, Davis TE, McFadden ET et al. Toxicity and response criteria of the Eastern Cooperative Oncology Group. Am J Clin Oncol 1982; 5 (6):649-55.*

19.7 Appendix 8: Body Surface Area calculation

The algorithm to be used in this study is Mosteller formula (1987):

BSA = √ [(Height (cm) x Weight (kg))/3600]

19.8 Appendix 9: Ophthalmic examination

An ophthalmic examination by an optometrist or ophthalmologist is required at screening and if clinically indicated during the study (e.g., monitoring of pre-existing cataracts, visual disturbances).

The examination is to include the following:
Prior to dilation:

- best corrected visual acuity
- slit lamp examination
- tonometry

Following dilation:

- fundoscopy
- slit lamp examination to document lens clarity

If a cataract/lens opacity is seen during the examination, the cataract/lens opacity will be graded according to a Grade 1-4 system (modified from Optometric Clinical Practice Guideline: Care of the Adult Patient with Cataracts: available on the American Optometric Association website: www.aoa.org).

Grading of Cataracts*				
Cataract Type	Grade 1	Grade 2	Grade 3	Grade 4
Nuclear<br>Yellowing and sclerosis of the lens nucleus	Mild	Moderate	Pronounced	Severe
Cortical<br>Measured as aggregate percentage of the intrapupillary space occupied by the opacity	Obscures 10% of intrapupillary space	Obscures 10% -50% of intra-pupillary space	Obscures 50% -90% of intra-pupillary space	Obscures >90% of intrapupillary space
Posterior subcapsular<br>Measured as the aggregate percentage of the posterior capsular area occupied by the opacitiy	Obscures 10% of the area of the posterior capsule	Obscures 30% of the area of the posterior capsule	Obscures 50% of the area of the posterior capsule	Obscures >50% of the area of the posterior capsule
*Designation of cataract severity that falls between grade levels can be made by addition of a + sign (e.g., 1+, 2+). Grading of cataracts is usually done when pupil is dilated.				

19.9 Appendix 10: PET/CT SCANs

FDG PET/CT imaging should follow the standardized protocol elaborated by EANM organization (*).

In particular, careful attention should be paid to the scheduled protocol (1 hour between FDG administration and PET acquisition), the glycemic status and, for each patient, unchanged technical parameters of acquisition.

**** FDG PET and PET/CT: EANM procedure guidelines for tumour PET imaging: version 1.0, Ronald Boellaard & Mike J. O'Doherty &Wolfgang A. Weber & Felix M. Mottaghy., November 2009 Eur J Nucl Med Mol Imaging DOI 10.1007/s00259-009-1297-4*** (available on http://www.eanm.org/publications/guidelines/index.php?navId=37)

19.9.1 Timing of FDG PET scans

- A baseline FDG PET/CT (PET0) is mandatory before treatment (within 28 days before C1D1), and the result form indicating at least one hypermetabolic lesion has to be faxed for allowing patient registration.
- A FDG PET/CT (PET3) has to be performed in the 10 days following the end of cycle 3 (for patients completing treatment) or within 4 weeks after the last drug administration for patients who permanently discontinue treatment for any reason before completing cycle 3.

19.9.2 Patient preparation

- Patients are not allowed to consume any food or sugar for at least 6 h prior to the start of the PET study (i.e. with respect to time of injection of FDG).
- Adequate pre-hydration is important to ensure a sufficiently low FDG concentration of FDG in urine (fewer artifacts) and for radiation safety reasons (for example, 1 l of water in the 2 h prior to injection).
- Parental nutrition and intravenous fluids containing glucose should be discontinued at least 4 h before the PET/CT examination. In addition, the infusion used to administer intravenous pre-hydration must not contain any glucose.
- During the injection of FDG and the subsequent uptake phase the patient should remain seated or recumbent and silent to minimize FDG uptake in muscles.
- Blood glucose level must be measured prior to administering FDG:
- If plasma glucose level is <7 mmol/l (or <120 mg/dl) the FDG PET study can be performed
- If plasma glucose level is ≥7 mmol/l (or >120 mg/dl) the FDG PET study must be rescheduled or the patient excluded depending on the patient circumstances.
- The following recommendations apply to patients with diabetes mellitus:
- type II diabetes mellitus (controlled by oral medication)
    - the PET study should preferably be performed in the late morning
    - patients must comply with the fasting rules indicated above
    - patients continue to take oral medication to control their blood sugar.
- type I diabetes mellitus and insulin-dependent type II diabetes mellitus
    - ideally, an attempt should be made to achieve normal glycemic values prior to the PET study, in consultation with the patient and his/her attending medical doctor
    - the PET study should be scheduled for late morning
    - the patient should eat a normal breakfast at 7.00 a.m. and inject the normal amount of insulin.
- Height and body weight must be determined at first scan and weight must be measured directly prior to each PET study because body weight often changes during course of disease.

19.9.3 PET scanner technical requirements

- FDG-PET scanning should be performed with a combined PET/CT for an improved data interpretation. Unless specifically excluded for particular protocols.
- Each patient is preferably scanned on the same camera for baseline and final evaluation.

19.9.4 PET acquisition and reconstruction

- The 18F-FDG injected activity will be defined according to on-site rules but should be >3.5 MBq/kg (or recommended activity for more recent PET-CT technology (TOF).

It is especially important to ensure that the time between tracer administration and starting of PET acquisition will be the same (± 5 min) at each PET scan:

- The patient should be positioned with the arm elevated over the head and PET acquisition should cover at least from the mid-femora to the external auditory meatus.
- A whole body acquisition with attenuation correction (non contrast-enhanced CT) and with emission scans of at least 2 minutes per bed position (or less for more recent PET-CT technology (TOF)) is started 60+/-10 minutes after FDG injection, starting from groin up to the head.
- FDG PET/CT imaging should follow the standardized protocol elaborated by EANM organization. In particular, a careful attention should be paid to maintain unchanged technical parameters of reconstruction within patient.
- A standard diagnostic CT scan with (i.v.) contrast agent may, if appropriate, be carried out according to standard radiological methods **after** the low-dose CT without contrast agent and PET acquisition.

19.9.5 PET Review LOGISTICS

19.9.5.1 Local FDG-PET reports

As soon as the inclusion of the patient is effective, the local nuclear medicine physician will be asked to upload PET reports on the Imagys platform: http://lysarc.imagys.com.

19.9.5.2 PET review Board

Local and the central analysis of the PETs should be done according to Lugano 2014 Criteria for PET.

The reviewer panel is composed by 3 nuclear physicians for review the PETs according to the following rules:

- 2 reviewers will analyze the PET scans independently.
- The local analysis will be taken into account if the 2 reviewers do not agree.

19.10 Appendix 11: Recommendations for Initial Evaluation, Staging, and Response Assessment of Hodgkin and Non-Hodgkin Lymphoma : The Lugano Classification

Bruce D. Cheson, Richard I. Fisher, Sally F. Barrington, Franco Cavalli, Lawrence H. Schwartz, Emanuele Zucca, and T. Andrew Lister. J Clin Oncol 2014;32(27):3059-68.

Revised Criteria for Response Assessment		
**Response and Site**	**PET-CT–Based Response**	**CT-Based Response**
**Complete**	**Complete metabolic response**	**Complete radiologic response (requires all of the following)**
Lymph nodes and extralymphatic sites	Score 1, 2, or 3 with or without a residual mass on 5 Point Scale**†**<br>It is recognized that in Waldeyer's ring or extranodal sites with high physiologic uptake or with activation within spleen or marrow (eg, with chemotherapy or myeloid colony-stimulating factors), uptake may be greater than normal mediastinum and/or liver. In this circumstance, complete metabolic response may be inferred if uptake at sites of initial involvement is no greater than surrounding normal tissue even if the tissue has high physiologic uptake	Target nodes/nodal masses must regress to ≤ 1.5 cm in LDi<br><br>No extralymphatic sites of disease
Nonmeasured lesion	Not applicable	Not applicable
Organ enlargement	Not applicable	Regress to normal
New lesions	None	None
Bone marrow	No evidence of FDG-avid disease in marrow	Normal by morphology; if indeterminate, IHC negative
**Partial**	**Partial metabolic response**	**Partial remission (requires all of the following)**
Lymph nodes and extralymphatic sites	Score 4 or 5**†** with reduced uptake compared with baseline and residual mass(es) of any size<br>At interim, these findings suggest responding disease<br>At end of treatment, these findings indicate residual disease	≥ 50% decrease in SPD of up to 6 target measurable nodes and extranodal sites<br>When a lesion is too small to measure on CT, assign 5 mm x 5 mm as the default value<br>When no longer visible, 0 x 0 mm<br>For a node > 5 mm x 5 mm, but smaller than normal, use actual measurement for calculation
Nonmeasured lesion	Not applicable	Absent/normal, regressed, but no increase
Organ enlargement	Not applicable	Spleen must have regressed by > 50% in length beyond normal
New lesions	None	None
Bone marrow	Residual uptake higher than uptake in normal marrow but reduced compared with baseline (diffuse uptake compatible with reactive changes from chemotherapy	Not Applicable

	allowed). If there are persistent focal changes in the marrow in the context of a nodal response, consideration should be given to further evaluation with MRI or biopsy or an interval scan	
**No Response or stable disease**	**No metabolic response**	**Stable Disease**
Target nodes/nodal masses, extranodal lesions	Score 4 or 5 with no significant change in FDG uptake from baseline at interim or end of treatment	< 50% decrease from baseline in SPD of up to 6 dominant, measurable nodes and extranodal sites; no criteria for progressive disease are met
Nonmeasured lesion	Not applicable	No increase consistent with progression
Organ enlargement	Not applicable	No increase consistent with progression
New lesions	None	None
Bone marrow	No change from baseline	Not applicable
**Progressive disease**	**Progressive Metabolic Response**	**Progressive disease requires at least 1 of the following:**
Individual target nodes/nodal masses<br><br>Extranodal lesions	Score 4 or 5 with an increase in intensity of uptake from baseline<br>and/or<br>New FDG-avid foci consistent with lymphoma at interim or end-of-treatment assessment	PPD progression<br><br>An individual node/lesion must be abnormal with:<br>LDi > 1.5 cm and<br>Increase by ≥ 50% from PPD nadir and<br>An increase in LDi or SDi from nadir<br>0.5 cm for lesions ≤ 2 cm<br>1.0 cm for lesions > 2 cm<br>In the setting of splenomegaly, the splenic length must increase by > 50% of the extent of its prior increase beyond baseline (eg, a 15-cm spleen must increase to > 16 cm). If no prior splenomegaly, must increase by at least 2 cm from baseline<br>New or recurrent splenomegaly
Nonmeasured lesion	None	New or clear progression of preexisting nonmeasured lesions
New lesions	New FDG-avid foci consistent with lymphoma rather than another etiology (eg, infection, inflammation). If uncertain regarding etiology of new lesions, biopsy or interval scan may be considered	Regrowth of previously resolved lesions<br>A new node > 1.5 cm in any axis<br>A new extranodal site >1.0 cm in any axis; if < 1.0 cm in any axis, its presence must be unequivocal and must be attributable to lymphoma<br>Assessable disease of any size unequivocally attributable to lymphoma
Bone marrow	New or recurrent FDG-avid foci	New or recurrent involvement

LDi: Longest transverse diameter of a lesion; MRI: Magnetic Resonance Imaging; PPD: cross product of the LDi and perpendicular diameter; SDi: shortest axis perpendicular to the LDi; SPD: Sum of the product of the perpendicular diameters for multiple lesions.

† Deauville criteria is a scoring system and it will be used for evaluation of tumor metabolic response on the F18FDG PET/CT performed at the EoT/PTD evaluation.

We will use a 5 points scale (adapted from the Deauville workshop in Leukemia & Lymphoma, August 2009; 50(8): 1257–1260), with new modifications (mainly 4 and 5 scales) (from Sally F. Barrington, N. George Mikhaeel, Lale Kostakoglu, et al. - Role of Imaging in the Staging and Response Assessment of Lymphoma: Consensus of the International Conference on Malignant Lymphomas Imaging Working Group - J Clin Oncol - Volume 32 - number 27 - September 20 2014).

It includes visual and quantitative analysis.

- 1. No uptake.
- 2. Uptake < mediastinum.
- 3. Uptake > mediastinum but < liver.
- 4. Uptake moderately more than liver uptake, at any site.
- 5. Markedly increased uptake at any site and/or new sites of disease.

Using these Deauville criteria:

TEP positive is defined by scale level 4 and 5 (as described above)

TEP negative is defined by scale level 1, 2 and 3.

19.11 Appendix 12: Pathological Samples Review

General principles and organization of the pathological review:

The SELINDA study requires a histological review of all cases included in the trial at diagnosis. The aims of the centralized histopathological review will be to **confirm the diagnosis of B-cell lymphoma,** according to the criteria of the updated WHO classification 2008 (1) for each patient in the SELINDA study. Histological criteria of inclusion and exclusion have been detailed in the current protocol.

The review process will be organized by the LYSA-Pathology institute, Hopital Henri Mondor, Créteil (LYSA-P).

Therefore for each randomized/included patient, tumor tissue blocks - or only when not possible - unstained slides will have to be sent for analysis and confirmation of diagnosis to LYSA-P.

Practical aspects of the LYSA review:

1. *Information on patient inclusion*

At patient's inclusion, the investigator will be requested to fax to LYSARC registration centre with the inclusion form a copy of the anonymized histopathological report used to include the patient in this trial on which the name and address of the pathologist having diagnosed the lymphoma will be easily identified.
LYSARC registration centre will then fax/mail these documents to LYSA-P.

2. *Sample request*

At reception of the pathological report and inclusion form, LYSA-P will sent to the initial pathologist a letter requesting :

- The paraffin block from the formalin fixed sample that was used to set the diagnosis and/ or 10 unstained Superfrost+ slides
- To notify LYSA-P of the presence of frozen tissue

3. *Sample centralisation at LYSA-P*

All these requirements (excluding frozen tissue) will be sent in prepaid envelope and centralized by LYSA-P at the following address:

LYSA-P, LYSA – SELINDA study,
Hôpital Henri Mondor - RDC Haut
51, avenue de Lattre de Tassigny, 94010 Créteil France

4. *Sample review*

At sample reception, routinely stained sections will be performed and an appropriate panel of antibodies according to morphological aspects will be applied. When sufficient slides are available, a pathological review will be organized at LYSA-P with the designated panel of pathologists for this study. All the cases will be reviewed by at least 2 expert hematopathologists and a consensus diagnosis will be set and registered in LYSA-P data base. This consensus diagnosis will then be sent to the clinical investigator and to the initial pathologist.
Initial tumor block will also be used to make tissue microarray (TMA), to study the expression of markers known to influence the prognosis of B-cell lymphoma.
For the need of the ancillary study, blocks will be kept temporarily to avoid a second request. Meanwhile, the block will be at the entire disposition of the initial anatomopathology laboratory under request if they need it.

19.12 Appendix 13: Biological samples for further ancillary studies

A) Rationale

The SELINDA study represents a unique opportunity to collect biological samples from patients with lymphoma that can be used to improve comprehension of the disease, better define the prognostic criteria and identify new factors that influence treatments results and outcome. These scientific studies will be performed as ancillary studies based on the SELINDA protocol.

B) Tumor biopsy

Further ancillary studies on tumor biopsies may also be conducted by the team in Rouen, under the supervision of Prof Herve Tilly. Rearrangements of MYC, BCL2 and BCL6 genes may be analysed using fluorescent in situ hybridization (FISH). GCB/ABC profile may also be determined by RT-MLPA, using unstained slides taken from the initial tumor block, provided by the LYSA-P.

The expression of markers known to influence the prognosis of B-cell lymphoma may also be analysed on TMA.

C) Plasma Samples

Proteomic plasma analysis appears to be a powerful tool to identify new markers of disease and new markers that could predict response to therapy. Plasma samples will be collected at individual centres under a standardized procedure and collected at the end of the trial by LYSARC.

A plasma sample will be collected from all patients who agreed to participate in the study.

A plasma sample should be drawn during screening or after patient's inclusion on the study on C1D1 before any treatment administration

Sample processing

LYSARC provided to centres tubes and labels for the plasma conservation. A traceability form for the conservation of plasma on site must be completed after each blood sampling, and faxed to LYSARC the day of blood collection (fax : +33 (0) 4 72 66 93 71)

10 mL of EDTA-anticoagulated blood should be processed as follows:

- EDTA tubes will be centrifuged **quickly after blood sampling** (3500 revolutions a minute for 10 minutes)
- Using a pipette, take the plasma and distribute it into 3 cryotubes (1.5 ml of plasma per cryotube maximum). Identify the cryotubes with the SELINDA - Plasma labels
- The aliquots will be stored at -80°C **within 3 hours following the blood sampling**.

All the samples will be gathered in Lyon after collection organized by LYSARC at the end of study (special carrier respecting cold storage). They will be stored at -80°C for exploratory analyses

All the samples will be collected in:

Centre Hospitalier Lyon Sud
Centre de Ressources Biologiques – SudBiothèque / Immunobiothèque
Bâtiment 3D
Chemin du Grand Revoyet – 69310 PIERRE-BENITE - FRANCE

D) Genomic DNA

The signature of informed consent form is required for the collection and sending of PAXgene DNA tube, the DNA banking and the analyses on the TUMORAL DNA. For the analysis of the CONSTITUTIONAL DNA, a signed consent for genetic analyse is mandatory.

DNA will be used to analyze markers known to influence disease outcome and response to therapy. Blood sample (8,5 ml on paxgene tube) will be collected in all patients of each site participating to the study at screening or after inclusion in the study on C1D1, before any treatment is initiated.

Sample processing

LYSARC provided to centres PAXgene DNA tube, labels and transport kits. A traceability form for the the sending of PAXgene DNA tube to Lyon Sud Hospital must be completed and joind at the sending (transport cost charged to LYSARC)

- Take a 8,5mL blood sample on a PAXgene DNA tube (Keep the tube vertically below donor's arm while sampling) Homogenise the tube with slow reversals
- Identify the tube with completed label provided by LYSARC
- Fill the traceability form for « PAXgene sending »
- Put the tube in the plastic bag and seal it. Then put the bag in the envelope and seal it
- Place the envelope and the traceability form together in the box and seal it
- Sending the tube by CHRONOPOST at :

Centre Hospitalier Lyon Sud
Laboratoire de biologie moléculaire et Hématologie
Centre de Biologie Sud – Espace Jacques Monot – Etage 1
A l'attention de Carole CHARLOT
Chemin du Grand Revoyet – 69310 PIERRE-BENITE - FRANCE

Alternative procedure for cell storage on site

If your center can't participate in this manner, cell preservation can be performed with the method of your choice.Assuring that your method will allow further DNA extraction and analysis.

Please refer to the traceability form for « Biological sample storage », fill in and fax it to LYSARC.

All the samples will be stored at:

Faculté de médecine Lyon Sud
Laboratoire Gille Salles
Chemin du Grand Revoyet – 69310 PIERRE-BENITE - FRANCE

19.13 Appendix 14: Representative list of the products containing GLUTATHIONE (GSH), S-ADENOSYLMETHIONINE (SAM) or N-ACETYLCYSTEINE (NAC)

As this list may not be exhaustive, the investigator should instruct patient to be vigilant on the composition of drugs and dietary supplements.

GSH-containing products		NAC-containing products		SAM-containing products	
**Product name**	**Ingredient**	**Product name**	**Ingredient**	**Product name**	**Ingredient**
Glutathione	glutathione	Antidote for acetaminophen overdose	acetylcysteine	SAM-e Complete	S-adenosyl-methionine
L-Glutathione	L-glutathione	Cerefolin NAC: medical food for age-related memory loss	L-methylfolate vitamin B12 N-acetyl cysteine	SAM-e	S-adenosyl-L-methionine
Glutathione reduced	glutathione	NAC	N-acetyl cysteine	Double Strenght SAMe 400	S-adenosyl-methionine
Reduced glutathione with alpha lipoic acid	Setria L-glutathione	N-A-C Sustain	N-acetyl L-cysteine		
Glutathione, Cysteine & C	glutathione L-cysteine Vitamin C	Best NAC Detox Regulators	N-acetyl cysteine		
(Mega-) Liposomal Glutathione	glutathione	N-acetyl-cysteine	N-acetyl cysteine		
Lypospheric GSH	glutathione	Fluimucil / Hidonac	N-acetyl cysteine		
Ivory Caps Skin Enhancement Formula	glutathione	Euronac	acetylcysteine		
Striagen-Ds	L-glutathione Vitamin C	Exomuc	N-acetyl cysteine		
Ophta'plex	L-glutathione vitamin C	Genac	acetylcysteine		
Anti-Age Cellules Femmes	L-glutathione vitamin C	Mycomystendo	acetylcysteine		
Farmagenetica - Antiox Premium	L-glutathione	Mucomyst	acetylcysteine		
Anti-Age Visage Femmes	L-glutathione vitamin C	Rhinofluimucil	acetylcysteine		
Immune Impulse Premium 30	L-glutathione	Solmucol	acetylcysteine		
Viteadetox	glutathione	Acetylcysteine	acetylcysteine		
Total Cleanse Foie	L-glutathione Vitamin C N-acetyl cysteine	Numetah G16 / Numetah G19	cysteine		
Vital Matrix Premium	L-glutathione N-acetyl	Visioplex Myrtille-	N-acetyl L-cysteine		

	cysteine	Ginkgo-Euphraise			
Protector Nutrients	L-glutathione L-cysteine vitamin C	Gold Specifics Antioxydant support	N-acetyl cysteine vitamin C		
Earth Source Multi-Nutriments	L-glutathione vitamin C	Actirub	N-acetyl cysteine vitamin C		
Compagnon Super anti-oxydant	L-glutathione L-cysteine vitamin C	Hepato calm'	N-acetyl cysteine		
Commando 3000	L-glutathione vitamin C	Pediaven (G15/G20/G25/ NN S/OE/NN1/ NN2)	acetylcysteine		
Ultra Cheveux Plus	L-glutathione vitamin C	Humex 100 mg	acetylcysteine		
L'âge d'or	L-glutathione vitamin C				
Advate	glutathione				
Advanced Antioxydant Formula	L-glutathione L-cysteine vitamin C				
Spectro Energy Multi-Vit-Min	L-glutathione vitamin C				
Multinature bien être et vitalité	L-glutathione vitamin C N-acetyl cysteine				

19.14 **Appendix 15 : Group B : R-GDP guidelines**

Day	Treatment	Dose		Administration modalities
**Day 1**	Hydratation	To begin the day before		
	Lasilix protocol	According to prescription		
	Tumor lysis prophylaxis	According to prescription		
	Anti-emetic therapy	Ondansetron 8mg	IV	
	Dexamethasone	40 mg	IV or PO	Maximum 1 hour before Rituximab
	Selinexor KPT-330	According to the determined dose level	PO	
	Premedication Rituximab	Paracetamol 1g<br>Polaramine 5mg	IV<br>IV	
	Rituximab	375 mg/m²	IV perfusion 1H30 ou 4H30	250 mL ou 500mL NaCl 0,9%
	Gemcitabine	1000 mg/m²	IV perfusion 30 min	250 mL ou 500mL NaCl 0,9%
	Cisplatine	75 mg/m²	IV perfusion 60 min	500 mL NaCl 0,9%
**Day 2**	Hydratation	According to prescription		
	Lasilix protocol	According to prescription		
	Dexamethasone	40 mg	IV or PO	
**Day 3**	Dexamethasone	40 mg	IV or PO	
	Lasilix protocol	According to prescription		
	Selinexor KPT-330	According to the determined dose level	PO	
**Day 4**	Dexamethasone	40 mg	IV or PO	
**Day 8**	Anti-emetic therapy	Ondansetron 8 mg	IV or PO	
	Selinexor KPT-330	According to the determined dose level	PO	
	Gemcitabine	1000 mg/m²	IV perfusion 30 min	250 mL ou 500mL NaCl 0,9%
**Day 10**	Anti-emetic therapy	Ondansetron 8 mg	IV or PO	
	Selinexor KPT-330	According to the determined dose level	PO	

19.15 Appendix 16 : Group B bis : R-GDP guidelines

Day	Treatment	Dose		Administration modalities
Day 1	Hydratation	To begin the day before		
	Lasilix protocol	According to prescription		
	Tumor lysis prophylaxis	According to prescription		
	Anti-emetic therapy	Ondansetron 8mg	IV	
	Dexamethasone	40 mg	IV or PO	Maximum 1 hour before Rituximab
	Selinexor KPT-330	According to the determined dose level	PO	
	Premedication Rituximab	Paracetamol 1g<br>Polaramine 5mg	IV<br>IV	
	Rituximab	375 mg/m²	IV perfusion 1H30 ou 4H30	250 mL ou 500mL NaCl 0,9%
	Gemcitabine	1000 mg/m²	IV perfusion 30 min	250 mL ou 500mL NaCl 0,9%
	Cisplatine	75 mg/m²	IV perfusion 60 min	500 mL NaCl 0,9%
Day 2	Hydratation	According to prescription		
	Lasilix protocol	According to prescription		
	Dexamethasone	40 mg	IV or PO	
Day 3	Dexamethasone	40 mg	IV or PO	
	Lasilix protocol	According to prescription		
Day 4	Dexamethasone	40 mg	IV or PO	
Day 8	Anti-emetic therapy	Ondansetron 8 mg	IV or PO	
	Selinexor KPT-330	According to the determined dose level	PO	
	Gemcitabine	1000 mg/m²	IV perfusion 30 min	250 mL ou 500mL NaCl 0,9%
Day 15	Anti-emetic therapy	Ondansetron 8 mg	IV or PO	
	Selinexor KPT-330	According to the determined dose level	PO	
